# An Assemblable, Multi-Angle Fluorescence and Ellipsometric Microscope

**DOI:** 10.1371/journal.pone.0166735

**Published:** 2016-12-01

**Authors:** Victoria Nguyen, John Rizzo, Babak Sanii

**Affiliations:** 1 Keck Science Department, Scripps College, Claremont, CA, United States of America; 2 Keck Science Department, Claremont McKenna College, Claremont, CA, United States of America; 3 Keck Science Department, Pitzer College, Claremont, CA, United States of America; Pennsylvania State Hershey College of Medicine, UNITED STATES

## Abstract

We introduce a multi-functional microscope for research laboratories that have significant cost and space limitations. The microscope pivots around the sample, operating in upright, inverted, side-on and oblique geometries. At these geometries it is able to perform bright-field, fluorescence and qualitative ellipsometric imaging. It is the first single instrument in the literature to be able to perform all of these functionalities. The system can be assembled by two undergraduate students from a provided manual in less than a day, from off-the-shelf and 3D printed components, which together cost approximately $16k at 2016 market prices. We include a highly specified assembly manual, a summary of design methodologies, and all associated 3D-printing files in hopes that the utility of the design outlives the current component market. This open design approach prepares readers to customize the instrument to specific needs and applications. We also discuss how to select household LEDs as low-cost light sources for fluorescence microscopy. We demonstrate the utility of the microscope in varied geometries and functionalities, with particular emphasis on studying hydrated, solid-supported lipid films and wet biological samples.

## Introduction

The specificity of a microscope’s design can limit its buildability. The more specifically a design is prescribed (e.g., part numbers from particular vendors), the less useful it will be as component markets change. The more generally a design is prescribed (e.g., a pure optical drawing) the more expertise and infrastructure is required in assembling the system. A successful middle ground is to supplement a component-specified assemblable design with associated design-methodologies to enable readers to adapt the system to their needs and markets [1, 2].

The principle benefit of an assemblable approach is that it requires little manufacturing expertise. Its applicability is enhanced by three recent developments. First, rapid prototyping instruments and services (e.g., additive manufacturing or “3D printing”) enable the ready transfer of fabrication expertise [3]. This transfer takes the form of design files that can be physically produced with 3D printers, which are now broadly available as part of institutional Maker-spaces [4, 5] or through services where the file is uploaded and a physical product is mailed to the customer [6]. Second, non-printable optical components can be sourced from multiple vendors as roughly comparable off-the-shelf parts, at a fraction of the cost of purchasing complete turn-key microscopes [7]. Finally, there are free open-source alternatives to otherwise expensive/manufacturer-specific microscope control software packages. These open source projects (e.g., *μManager* [8]) enable low cost integration across multiple hardware platforms.

Here we present an assemblable design for a multi-functional optical microscope with upright, side-on and inverted fluorescence, as well as ellipsometric imaging capabilities—a design we name the SwingScope. The multi-functionality of the device enables capabilities beyond cost/space savings. For example, samples can be imaged from multiple angles without being perturbed, and complementary techniques such as fluorescence and label-free ellipsometry simultaneously measure thin-films.

In addition to a specific plan, we explain the customizable design methodology, and demonstrate multi-functionality. Combined fluorescence, imaging ellipsometry and multi-angle imaging implementations are not currently in the literature, as far as we are aware. While markets are subject to change, we demonstrate that the parts required for the combined upright and inverted fluorescence microscope and imaging ellipsometer currently costs less than $16k, and can be assembled from a detailed and provided manual (see Supporting Information, S1 Appendix) by two undergraduate students in less than a day. This is at least an order of magnitude cheaper than purchasing the three separate comparable commercial systems [7], and is of sufficient quality to produce research publications [9]. In this paper we discuss how to customize components in anticipation of rapidly evolving markets of available components (e.g., fluorescence filters, light sources, and cameras). We also include demonstrations of the various functionalities, with samples ranging from molecular films to cells and tissues.

## Methods and Methodology

### Design Overview

**Fig 1:**
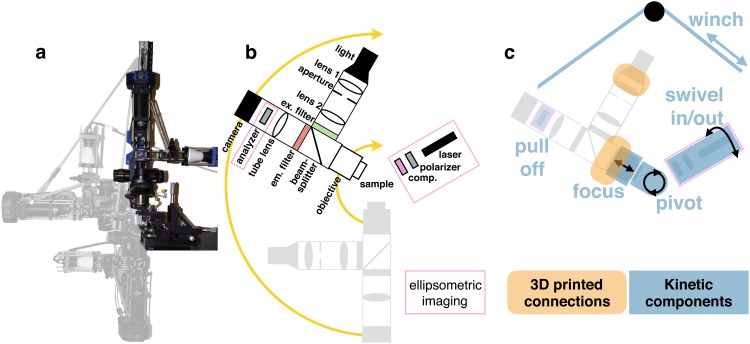
Swingscope. **a:** Superimposed images of the microscope in three geometries, with the imaging ellipsometry components removed for visibility. **b:** Schematic of optical components in the inverted (gray) and oblique configurations. Components inserted for imaging ellipsometry are outlined in magenta. **c:** Schematic overlaid with moving and 3D-printed components.

The salient mechanical feature of the SwingScope design is that the microscope can pivot 180° vertically around the sample, between upright and inverted geometries. This is achieved by mounting the microscope optics to a vibrationally damped rod that has a pivot point roughly aligned with the sample (see Fig 1). An inverted geometry is preferable when imaging samples submerged under water [10], while an upright geometry is useful when studying an air/water interface [11], or non-invertible biological samples. Additionally, side-on imaging enables determining the contact angle and capillary flow of microscopic droplets on surfaces [12, 13], and oblique incident imaging is useful in ellipsometric applications such as thin films [14]. The microscope freely swings through the range of angles.

At any pivot geometry, the SwingScope converts from a brightfield microscope to a fluorescence microscope by manually swapping filter cubes. When imaging ellipsometry capabilities are desired, a laser-illumination arm manually swings into the beampath and a rotating polarizer slides onto the microscope posts. The system as designed is largely manual, with the exception of camera control and acquisition which are controlled by readily extensible *μManager* open-source software.

We include a detailed manual (S1 Appendix) on the assembly of the instrument. However, the microscope includes several components which strongly impact price and performance, and whose specification is likely to evolve quickly with current markets. To that end, their selection rationale is detailed below. In particular, we discuss how the light source could be a consumer LED bulb, the fluorescence filters could be multi-spectral and swappable, and cameras has several lower-cost options. All parts in the microscope are either off-the-shelf components or rapidly prototyped (i.e., 3D printing/laser-cutting), to reduce required manufacturing expertise.

### Mechanical Pivot

Our design methodology includes a pivot aligned with the anticipated sample position, which allows changes in angle with only a minimal change in sample focus position. The base of the pivot and the base of the sample-holder cannot attach to the table at the same place, and so must be offset in the direction transverse to the rotation, while the optical elements must still align at the sample. We chose to place the microscope mount further away from user, to keep the sample more accessible.

We chose a freely-swinging, sturdy pivot with attached platforms on both ends. This is convenient for easy imaging with 40X objectives or below, however a damped pivot would have enable higher magnification objectives to be used with less concern about table vibrations.

Concerns about safety and a desire to have a continuous range of available angles led us to introduce a secondary angle-stabilization mechanism. A post taller than the microscope is mounted on the same table and affixed with a pulley. Steel cable runs from the top of the microscope post through the pulley to a manual winch, which allows us to continuously set the angle of the microscope, and provides a measure of safety should the single pivot fail.

### 3D Printed Stabilization and Adapter

Necessary mechanical components that cannot be sourced commercially must be fabricated. Our design includes two such components: an alignment piece between the focus block and the rest of the assembly, and a mechanical adapter for our light source. These components were designed procedurally using an open-source software package called OpenSCAD [15] and printed on a consumer additive manufacturing (3D printing) system (Afinia 3D, Chanhassen, MN). The OpenSCAD design files and printable stereolithograph (.stl) files are included in S2 File.

### Fluorescence and Brightfield Microscopy Lightpaths

A broad-band white light source is mounted on a pathway perpendicular to the microscope post, and passes through two lenses that focus it on the back focal plane of the objective, in a configuration (Köhler [16]) that evenly illuminates the sample. In between the lenses is an aperture that is imaged onto the sample so that the illumination is “field apertured”. This feature is particularly useful for geometric exposure techniques such as Fluorescence Recovery After Photobleaching (FRAP [17]).

The light then passes through a manually interchangable cube that filters the white light to the absorption spectrum of the fluorescent molecules at the image plane. A diagonal beamsplitting filter in the cube, called a dichroic, reflects the beam towards the objective/sample while also partially filtering it. This filter cube can be readily swapped to accommodate different fluorophores, or be replaced with unfiltered glass to enable a non-fluorescence bright-field imaging mode.

The excitation-filtered light passes through the filter-cube to an objective lens attached to a manual focus-block. The alignment between the focus block and the illumination/detection portion of the microscope is critical, and requires another custom 3D-printed component for rigidity. If the sample illumination is uneven, it is often this alignment that needs adjustment, even with the rigidity enhancement. The light returning through the objective is re-filtered by the filter-cube to reject excitation wavelengths and isolate the emission from the sample fluorophores. It passes through a high quality tube lens before striking a camera placed at its focal length. We have experimented with a plano-convex lens, a fused doublet lens and a lateral and axial chromatically corrected fused doublet lens, and found the performance of the corrected doublet lens to merit the greater price (2.0*μm* vs 3.5*μm* resolution at 10X, see S3 File).

Selection of the objective lens is also critical. Because fluorescence absorption and emission wavelengths are significantly different, objectives that correct for chromatic aberration are preferable for fluorescence applications. Such objectives can be expensive, and must be chosen with applications in mind. In our design we specify a 10X objective and a long working distance 40X objective with a built-in adjustment collar for imaging through different thicknesses of coverglass. Objectives beyond 40X are often significantly more expensive (e.g., up to the cost of our entire microscope) and are more prone to revealing table vibrations. Additionally, we chose infinity-corrected objectives, which do not tightly prescribe the distance between the objective and the rest of the optics, which simplifies microscope customization.

### Filters and light sources

**Fig 2:**
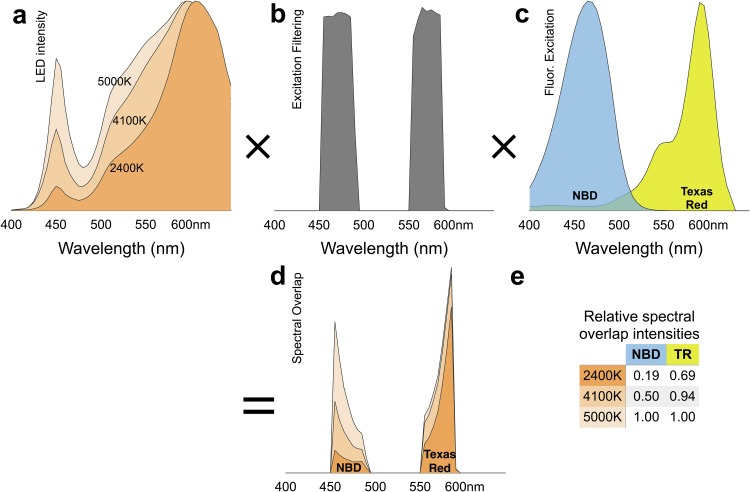
Calculating light-source, filter and fluorphore spectral overlap. **a:** Emission of three consumer LED bulbs (TCP LED10P20D24/ 41/ 50KNFL) of labelled color temperatures. **b:** Transmission spectrum of fluorescence excitation filterset (Chroma 59022). **c:** Excitation spectra of NBD and Texas-Red dyes commonly used in our laboratory. **d:** The product of multiplying the spectra in a/b/c together. **e:** Relative efficiency of the different light sources for the two probes, determined by integrating the area under the curves of the spectra in d. Here the 5000K bulb is best suited for our fluorophores.

Our approach is to target two spectrally distinct fluorophores and optimize the filters and light sources accordingly. The common fluorophores in our laboratory are Texas-Red and NBD (N-(7-Nitrobenz-2-oxa-1,3-diazol-4-yl), which have largely separate excitation and emission spectra (see Fig 2). We selected optical filters from a manufacturer (Chroma, Bellows Falls, VT) that could excite and detect emission from both of these dyes at the same time, to reduce cost with a tolerable efficiency diminishment. Our instrument design allows for rapid swapping of filter-cubes without realignment should higher efficiency single-fluorophores filter sets be required in the future.

Recent advances [18] in consumer LED light bulbs has availed a broad range of efficient, long-lasting and low-cost options [19]. We found a form-factor that fit well in our system (PAR20) and mounted it onto a 3D printed cage-adapter with an E26 light cord (Ikea). We measured the spectra of several LED light bulbs using a spectrophotometer (CC100, Thorlabs) and determined the best spectral overlap with our two fluorophores, including the fluorescence filtering of the microscope. A comparison of three color temperature bulbs from the same manufacturer is shown in Fig 2. This approach is prone to some ripple flicker. Comparable LEDs from the same manufacturer have a 191Hz 3.75% ripple [20], but the fluctuations are negligible for exposures longer than 50ms.

### Detector/Camera selection

Fluorescence microscopy cameras tend to be one of the most expensive components of a microscope. There are several key features to consider. Camera sensitivity is crucial for fluorescence detection efficiency, while frame-rate strongly affects user-experience and noise/resolution characteristics determine image quality.

However, there are options in adjacent consumer fields with tolerable compromises that are orders of magnitude lower in cost. We experimented with low cost cameras from consumer photography (Canon 20D, 60D) and consumer astronomy (QSI 628s), with success. Cameras are rapidly evolving products and market surveys are prudent at the time of microscope assembly [21]. We currently use the astronomy camera, largely due to its higher grayscale sensitivity and its higher frame-rate that facilitates focussing. To aid in future searches, we list some key camera characteristics and our design methodologies in Table (1).

**Table 1:** Feature critera for microscope camera selection.

Characteristic	Design methodology
Sensor size	Approximately the diameter of smallest microscope optic. Smaller to avoid vignetting.
Binning capability	Useful for weak fluorescence imaging
Transfer frame-rate	Sufficiently large for focusing at high binning (approximately 3 frames per second)
Sensor cooling	Diminishes thermal noise for long exposures
Electronic Read-Noise	Largely non-thermal image noise
Quantum Efficiency	Determines sensitivity, lowering exposures
Mechanical shutter	Lessens dust on sensor, enables dark-substracted exposures
Software	*μ*Manager compatibility is useful, although cameras typically come with simple image capturing software
Color	A color camera will have significantly less sensitivity as light will be re-filtered, but it will allow easy distinction between multiple fluorophores

### Imaging Ellipsometry

Ellipsometry is a mature, label-free, non-contact optical technique for determining the properties of thin films by how they change the polarization of obliquely incident light. Its operational and modeling aspects are deeply explored elsewhere [14, 22], but briefly parallel polarizations interact with a surface differently than transverse polarizations. This difference manifests as a change in the total polarization of the light, and is sensitive to surface properties and adherent thin films. In practice, we measure the surface-induced change by finding the right combination of incident polarizations such that the reflected polarization is a single linear polarization, which is readily detectable by minimizing intensity at the detector with a polarizer. This approach is called “nulling,” and the polarization angles of nulling can be computationally modeled to determine surface parameters such as the thickness and refractive index of an adhering film. While the spatial resolution is limited by diffraction optics, the thickness-resolution is typically sub-nm. The technique has applications in semiconductor industries [23] and biology, particularly supported lipid bilayers [24, 25]. Imaging ellipsometry is a variant of the technique which spatially resolves thin films on the surface [26].

To control the incident polarization we use a combination of a laser, linear polarizer (P) and birefringent compensator (C) to produce the namesake elliptical polarization that strikes the sample at an oblique angle. In “nulling” conditions the polarizer and compensator are rotated so that after striking the surface the light is linearly polarized. It then passes through a analyzing polarizer (A) that is rotated 90 degrees from the reflected polarization, resulting in a minimum signal passing through (“null”).

Mechanically, in our multifunctional microscope the optical components of ellipsometry should be removable from the light path, as these optical elements are not necessary during fluorescence imaging. Our design places the incident optical components on a rigid cage system attached to a ball-mount that can freely enter and exit the light path (see Fig 1). A low power 532nm wavelength collimated laser diode module (CPS532, Thorlabs) is the light source, and angle alignment is performed by way of a digital tilt sensor (iGaging, San Clemente, CA). This design, coupled with the SwingScope’s 180 degree range of motion allows for a large range of incident angles for optimizing imaging ellipsometry applications. A rotatable polarizer near the detector also slides on and off the four-post rails in the optical path immediately after the tube lens.

Three of the optical components of imaging ellipsometry need to be rotatable. There are many commercial systems for rotating optical components, both manual and motorized. For contrast, a key design parameter is the resolution that the angle can determined. For quantitative ellipsometry suitable for modeling (which we do not pursue here), motorization of the polarization angles (and focus knobs) would be beneficial.

To perform imaging ellipsometry with underwater samples the optical geometry is vastly simplified by orienting the windows of the wet sample-cell to be perpendicular to the incident beam. Our approach is to laser-cut two triangles out of acrylic as ends of a sample-cell, which are then placed on a laser-engraved base with two 22x50mm coverslips leaning on the triangles as the laser windows. The sample is enclosed in the cell with rapid-curing silicone polymers (Microset 101, Leicestershire, U.K.). The laser-cutting documents are included in the S1 File, and like 3D printing there are online services for laser-cutting parts from files if a local instrument is not available [27].

### Ellipsometric sensitivity

While “nulling” determines the quantitative polarization change of the surface [28], imaging contrast can be achieved when “off-null.” For example, if the sample is a thin-film stripe on an otherwise plain substrate, one could “null” the substrate by setting the P/C/A angles so a minimum of light strikes the camera [22]. However, the adjacent stripe would have different nulling conditions and so its intensity would not be minimized at the same P/C/A angles [29]. This difference is the origin of the contrast in an “off-null” image, enabling the observations of the morphology of the stripe. The principal advantage of ellipsometry is that small differences in thickness and/or refractive-index result in large differences in the null angles of the Polarizer (P) and Analyzer (A) polarization optics (see Fig 1), producing label-free contrast for images of even molecularly thin films.

Ellipsometric sensitivity can be tuned for specific samples, substrates, environments, wavelengths and incident angles. The importance of this tuning to instrument design is largely in the specification of the polarization angle resolution components. By tuning to greater sensitivity we can afford to purchase less precise polarization rotation optomechanics. Additionally, when analyzing a specific sample, a greater difference in the angles of the polarization optics correlates with greater imaging contrast. This contrast is the basis of a subset of imaging ellipsometry called Brewster Angle Imaging [30], which our microscope design is also capable of achieving without modification.

**Fig 3:**
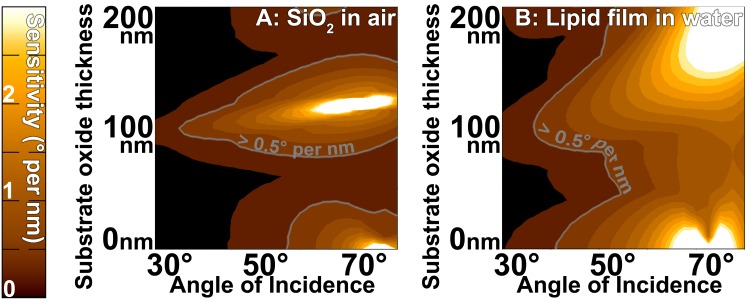
Ellipsometric Sensitivity. Optimizing ellipsometry sensitivity with angle of incidence and substrate oxide thickness, using an ideal thin-slab dielectric theoretical model. Parameters are for a 532nm wavelength laser and substrates consisting of Silicon with various oxide thicknesses. Sensitivity is measured in degrees of P or A rotation per nanometer of added material, noted by the left colorbar, and also by the gray isolines at 0.5 degrees per nm. The map of which regions are more sensitive in P or A is included in S3 File. **a:** Sensitivity for 1nm of additional Silicon oxide in air **b:** Sensitivity for 1nm of lipid material in water.

Two parameters that are convenient to tune are substrates and incident angles. Silicon substrates can be readily purchased with a range of surface oxide thicknesses, and the angle of the ellipsometer for the SwingScope is adjustable. To explore these tunable parameters we consider semiconductor and biomaterials examples: the thickness of an oxide of Silicon in air, and the thickness of a lipid bilayer under water. We mapped the theoretical P or A angle sensitivity of each type of sample to a one nanometer increase in sample thickness using a thin dielectric slab model [31] implemented by E. Kondoh [32], for a range of incident angles (30–80 degrees) and substrate oxide thicknesses (0–200nm), under ideal conditions (Fig 3).

We found regions of significantly greater sensitivity within this tunable-parameter space. Notably a polarizer angle resolution of 0.5 degrees corresponds to single nm or better height resolution for both types of samples over a significant parameter space for both our representative semiconductor and bio-materials (marked on Fig 3). Such optimization guides us to make design decisions. For example, with this theoretical guidance we purchased mechanical rotation elements with 0.1 degree resolution, Silicon substrates with native and 100nm oxides, and will operate the SwingScope at 60–70 degrees of rotation from normal. Further angular determination can be achieved by fitting intensities at measured angles to a parabola in order to determine the nulling angle with greater accuracy [33].

## Results

### Multi-angle Imaging

**Fig 4:**
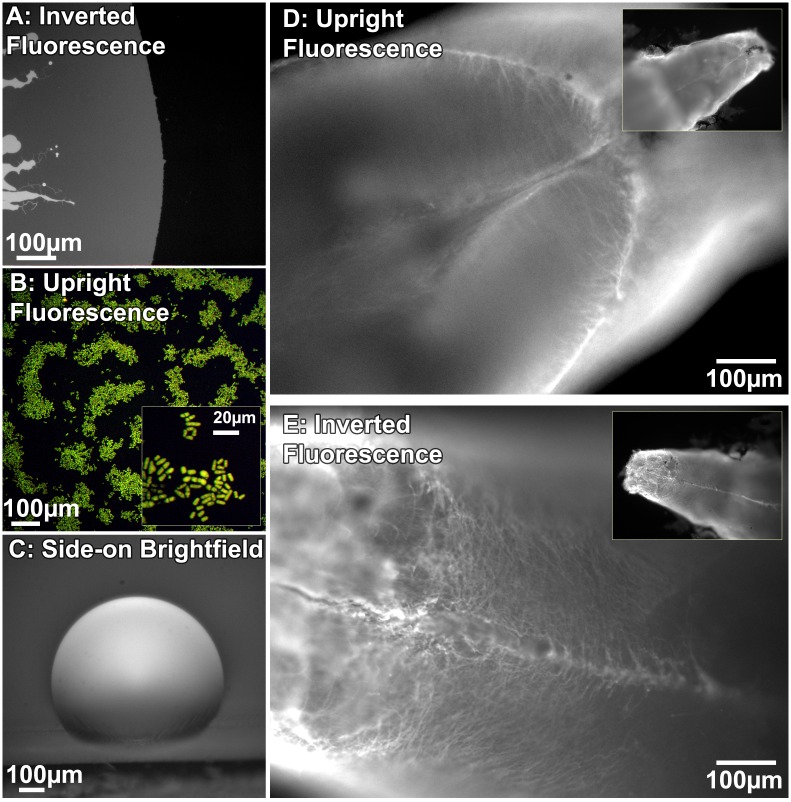
Images from the microscope in different geometric configurations. **a:** Inverted fluorescence image of lipids (97% DOPC, 3% NBD) spreading [37] as a single bilayer out from a lipid-rich stamp on glass, in PBS buffer. **b:** Upright fluorescence image of lid1+ mRNA in S. pombe fission yeast cells harboring the “green RNA” system [38] at 10x and 40x (inset) with a Canon DSLR camera, courtesy of the Tang Lab. **c:** Side-on imaging of a water droplet on a strip of teflon tape.**d: and e:** Upright and inverted fluorescence images of the same adult zebrafish brain, revealing distinct morphology. Sample was dissected whole and subjected to PACT (passive clarity technique) with immunohistochemistry for primary antibody mouse gfap (zirc) and secondary antibody goat anti-mouse fitc-488 (abcam) [39, 40]. All zebrafish protocols were IACUC approved, courtesy of the Spence Lab. Insets are lower magnification (4X) images of the area.

The choice of imaging angle is largely determined by sample requirements or the placement of associated instrumentation, such as patch-clamps and sample life-support. For example, inverted microscopy is particularly useful when the sample is immersed in an open liquid container such as a petri dish. In such cases inverted imaging avoids an air-liquid interface which produces significant optical distortions from even subtle mechanical movements/vibrations due to surface waves. Additionally, evaporation would change the focal plane, which would limit the long term stability of an upright experiment. In our laboratories we primarily use the SwingScope to observe fluorescent phospholipid bilayers immersed in buffer, spreading on glass coverslips at the bottom of an open petri dish (e.g., Fig 4A). The specific lipids used here are mixtures, as noted, of 1,2-dioleoyl-sn-glycero-3-phosphocholine (DOPC, from Avanti Polar Lipids) and 1,2-dioleoyl-sn-glycero-3-phospho-L-serine (DOPS, from Avanti Polar Lipids), and fluorescent (N-(7-Nitrobenz-2-oxa-1,3-diazol-4-yl)-1,2-dihexadecanoyl-snglycero-3-phosphoethanolamine (NBD-PE, from Biotium) in Phosphate Buffered Saline (PBS, Boland Scientific). This sample is best suited to an inverted microscope geometry.

Wet samples between glass slides and a coverslip can be readily imaged in either upright or inverted modes (e.g., Fig 4B is an upright image of yeast cells). Upright microscopy would be also be useful when imaging the air-water interface, or with living samples that have an upright preference. Typically, oblique imaging is less useful as it is difficult to align the focal plane with a significant portion of the sample, however side-on imaging at 90 degrees is particularly useful for determining profiles. For example, one method to measure the surface tension of a liquid or the surface energy of a solid is to determine the vertical profile of a droplet on the horizontal surface [34]. We would place a droplet on a horizontal solid, and orient the microscope so it is imaging directly from the side at 90 degrees(e.g., water on teflon in Fig 4C). Subsequent image analysis would determine the contact angle. Contact-angle imaging does not typically require microscopy, though smaller droplets require less correction for the effects of gravity. However, for smaller sized substrates smaller sized droplets could be required, which would mandate microscopic side-on imaging. Multi-angle imaging is also useful for studying a sample with different morphology on different sides, without moving the sample (Fig 4D and 4E).

### Ellipsometric Off-null Imaging

**Fig 5:**
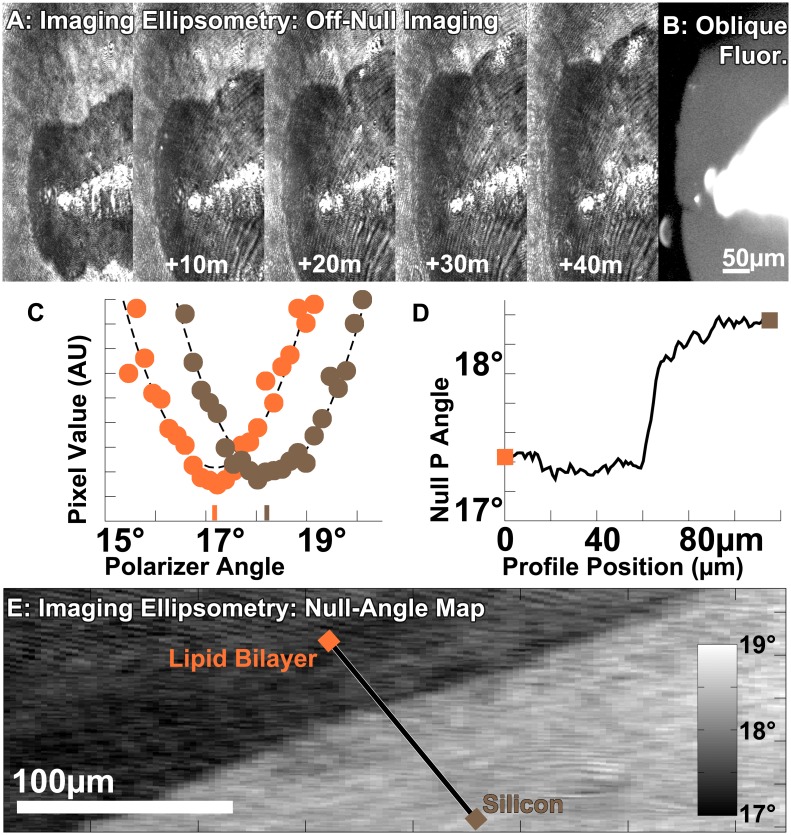
Ellipsometric Imaging. **a:** Time-series of contrast ellipsometric images of lipids (82% DOPC, 15%DOPS, 3% NBD-PE) spreading from a source on Silicon with a 100nm of oxide in PBS solution, without a fluorescence filter-cube in the beampath. Images have been corrected for oblique incidence geometric distortion, and the contrast globally enhanced for visibility. **b:** Fluorescence microscopy image of the same location without moving the sample, by inserting the fluorescence filter-cube and disabling the ellipsometry laser. **c:** Null-angle as determined by fitting the raw intensity vs. polarizer angle to a parabola. **d:** Null polarizer angle as a function of position across the profile noted in e. **e:** Null map of the edge of the a lipid bilayer with the same composition, on bare Silicon in PBS buffer.

We used imaging ellipsometry to observe the spreading [9] of a single lipid bilayer (5.5nm thick [35]) on Silicon with a 100nm thermal oxide, inside a wet-cell filled with PBS buffer (Fig 5). Although the lipid layer was molecularly thin, it produced sufficient contrast for imaging ellipsometry. We were able to determine lipid bilayer spreading morphology and kinetics as it spread outward from the stamped source (Fig 5A). The contrast was not derived from fluorescent labelling, but from the difference in the surface polarization due to the bilayer.

A unique capability of the SwingScope is that it can perform imaging ellipsometry and fluorescence microscopy without moving the sample. For example, we acquired a fluorescence image of the same lipid film (Fig 5B). Here we used fluorescence microscopy to initially find the sample on the surface, and to positively identify it as a lipid film and not contamination. In principle, the orthogonality of fluorescence and ellipsometry could be used to identify interactions between films and analytes, for example protein-binding. As the ellipsometry is a label-free technique, the combination could also be used to test the hypothesis that a thin film fluorescence distribution correlates to the sample distribution.

A characteristic of the oblique imaging required for ellipsometry is that the focal plane intersects the sample plane. This results in only a single line of the sample being in focus at a time, which can be corrected for by gathering images at a range of focal positions and integrating them into a single focussed image (see S3 File). This process would strongly benefit from motorization. In practice, at 10X magnification we find that the depth of field is sufficiently large to capture significant portions of the sample with sufficient focus to be resolvable with imaging ellipsometry. The artifact is more pronounced with fluorescence imaging, as the illumination is also defocussed away from the focal plane. Additionally, images must be corrected for geometric stretching due to the oblique incidence.

### Ellipsometric Null Mapping

Ellipsometric mapping determines the null conditions at every pixel and represents the null angles as an image. The resulting map appears visually similar to an off-null image, but with less artifacts such as laser-interference fringing and uneven illumination. Our approach to generate a null map is to collect images at different polarizer angles (e.g., varying P), when close to null conditions for part of the sample. For example, we null the image on the silicon next to the bilayer by adjusting the P/C/A angles. We subsequently gather a series of images, each at a different P angle. Analytical scripts (written in Octave [36]) extract a location’s pixel-intensity across all of the images, effectively producing an intensity vs. P angle graph (Fig 5C). The script fits polynomials to these graphs, and determines the minimum P-angle. The map consists of the null angle of polarization at each pixel, which varies significantly as we cross molecularly thin lipid films on Silicon in buffer solution (Fig 5D and 5E).

In principle, the null angles can be quantitatively used to determine surface polarization changes, which can be feed into models to determine surface thickness/refractive-index. In practice we find that the lack of motorization severely limits the repeatability of angle measurements, and the rate at which null data can be acquired. For quantitative imaging ellipsometry we are currently exploring motorization using open-source approaches [41], but currently ellipsometry specific software for control and analysis is not widely available. For the purposes of an assemblable system we suggest using the imaging ellipsometry capability as a means of achieving label-free contrast, which is particularly useful as a control on easier to perform fluorescence experiments.

## Conclusions

We introduce a multi-functional microscope called the SwingScope, with fluorescence and imaging ellipsometric capabilities at a broad range of incident angles around the sample. We present it as an assemblable design with a high-level optical plan, a set of low-level assembly instructions, and an adaptable design methodology. Components are either off-the-shelf or rapidly prototyped with consumer 3D printers, which reduces cost and fabrication complexity. The instrument is uniquely multi-functional, allowing inverted, upright, oblique and side-on fluorescent, brightfield and ellipsometric imaging. The multifunctional design enables cost and space savings to meet laboratory needs that may have require multiple instruments to achieve some of these capabilities.

The expected initial utility of this instrument is in small research laboratories, which may have neither the space nor the funds for dedicated commercial equipment that fulfills all of the functionalities of the SwingScope. Recently, several low-cost, portable and innovative microscopes have been introduced in the literature [42–46]. Their focus is largely on field-diagnosis imaging. Laboratory research typically requires long term stability, adaptability and higher image quality than specialized diagnosis applications. A recently published report adapted used components to enable fluorescence imaging with bright-field microscopes, and also included a microscope design with components largely available from a hardware-store [47]. This approach resonates with the assemblable design methodology we adopt here. The SwingScope project also fits within and benefits from the the open source do-it-yourself (DIY) approaches in terms of functionality and assemblability. For example, many publications listed in the PLOS collection on Open Source Toolkit:Hardware [48] can be incorporated into this microscope design to enable functionality and cost savings [2, 41, 49–51].

We demonstrate the multi-functionality of the microscope in several fields, namely biomaterials (supported lipid bilayers in buffer), biology (yeast cells and brain tissues), physical chemistry (micro droplet contact angles). These applications were with wet or liquid-immersed samples. Additionally we include design methodology to allow customization for market changes (e.g., camera and light source), specific sample needs (fluorescence filters and sources), and specific functionalities (e.g., ellipsometer substrates). This assemblable design is intended to reduce costs and increase flexibility for research labs, and is used as such in our laboratory [9]. However, its ease of assembly and customizable design may also lend itself to to pedagogical applications—for example, a teaching laboratory where students rebuild the instrument and apply it to various experiments throughout the semester.

## Supporting Information

S1 Appendix SwingScope Assembly Manual. Detailed instructions for assembly of SwingScope, and associated parts list.
(PDF)

S1 File Diagrams for laser-cutting components for the ellipsometric wet-cell. (ZIP)

S2 File 3D printable files (openSCAD and .stl). (ZIP)

S3 File Imaging resolution for variously priced tube lenses; example of combining slices of multiple oblique images to form a single focussed one; map of P vs. A increased ellipsometric sensitivity. (PDF)
