# Supplementary material for: An Assemblable, Multi-Angle Fluorescence and Ellipsometric Microscope
Source: PLoS One. 2016 Dec 1;11(12):e0166735. doi: 10.1371/journal.pone.0166735 (PMC5132209; doi:10.1371/journal.pone.0166735)
Supplement: S1 Appendix — Detailed instructions for assembly of SwingScope, and associated parts list. (PDF) [file pone.0166735.s001.pdf]

SwingScope Manual

Victoria Nguyen [*], John Rizzo [†], and Babak Sanii [‡]

Keck Science Department, Claremont McKenna, Pitzer, and Scripps Colleges, Claremont, CA 91711

Revised: April 1, 2016

Abstract

The specialized functionality and high cost of commercial quality optical microscopes can be a significant barrier to teaching/research laboratories with constrained resources. In order to address these broader needs, as well as to meet specific biophysics imaging needs in our lab, we have designed an economical, multifunctional microscope. This microscope, called the SwingScope, combines functions traditionally fulfilled by two separate fluorescence microscopes, one upright and one inverted, and an imaging ellipsometer. It utilizes commercially available optical components and requires less than one day for an undergraduate student to assemble. The cost of the microscope is between \$14-16k depending on the desired capabilities, which is an order of magnitude cheaper than an upright and inverted commercial fluorescence microscope. The detailed plans and component lists will be made available as free documents, and the system is open-source whenever possible (ImageJ, μManager, Arduino). Additionally the SwingScope can rotate 180°around the sample, enabling contact-angle measurements as well as 3D reconstruction techniques. This rotation is particularly important for imaging ellipsometry purposes. We have used the SwingScope to measure dynamics of single supported phospholipid bilayers (1% fluorescence), and preliminary point-spread functions indicate a 2μm resolution at 10x magnification. In addition to its research applications, we are exploring its assembly and use for an undergraduate teaching lab.

[*]Electronic address: `victoria.hv.nguyen@gmail.com`
[†]Electronic address: `jrizzo15@students.claremontmckenna.edu`
[‡]Electronic address: `bsanii@kecksci.claremont.edu`

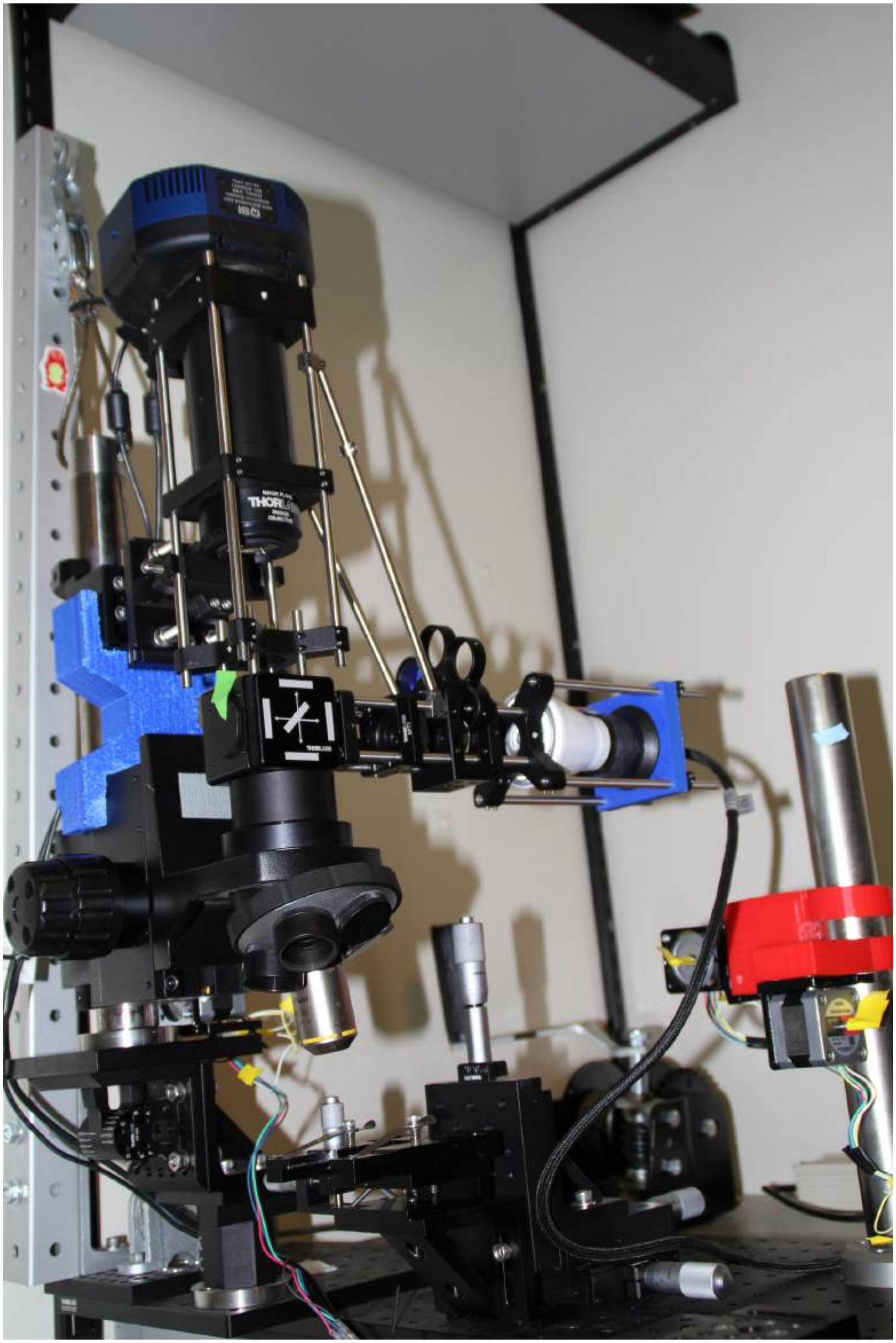

Figure 1: The fully assembled SwingScope

Contents

1 SwingScope Design 6

2 Characterization 8

3 Capabilities 9

4 Costs 10

5 Itemized Parts List 11
5.1 Recommendations for Customizing Parts 14

6 Codes for 3D Printed Parts 14
6.1 LED Adapter 14
6.2 H-shaped Piece 15
6.3 3D Printing Instructions 16
6.3.1 OpenSCAD 16
6.3.2 Afina3D Printer 16

7 Before You Start Building 18
7.1 Screws and Threads 18
7.2 Cleaning and Handling Optics 18
7.3 Building Tips 18

8 Assembly Procedure 19
8.1 Mounting Post 19
8.2 Rotation Platform Post 19
8.3 Mount-to-Cage Extension 20
8.4 Sample Stage 24
8.5 Rotation Platform 25
8.6 Kohler Illumination Arm 28
8.6.1 LED Light Source 28
8.6.2 Filter Wheel (Optional) 28
8.6.3 Illumination Arm 29
8.7 Upright 60mm Cage System 33
8.7.1 Cube to Camera 33
8.7.2 Adding Tube Lens 37
8.8 Filter Cubes 39
8.8.1 Brightfield Filter Cube 39
8.8.2 Fluorescence Filter Cube 40
8.9 Pulley System 41
8.10 Ellipsometer 44
8.10.1 Analyzer 44
8.10.2 Ellipsometer Mount Stage 45
8.10.3 Laser Cage 48
8.10.4 Ellipsometry Arm 49

9 Installation Preparation 51
9.1 Microscope Table for Brightfield and Fluorescence 51
9.2 Microscope Table for Ellipsometry 53
9.3 Computer Table/Cart 53

10 SwingScope Installation Procedures 54
10.1 Diagrams for Installation 55
10.2 Pulley System 57
10.3 Rotation Platform 58
10.4 Mounting Post 59
10.5 Focus Block 60
10.6 Mount-cage Extension 61
10.7 Secure Mounting Post 62
10.8 Sample Stage 62
10.9 Upright Cage System 63
10.10 Kohler Illumination Arm 64
10.11 Objectives 65
10.12 Camera & Filter Cube 67
10.13 Ellipsometer 67

11 Electronics 68
11.1 Cord Lengths 68
11.2 Camera Cords 68
11.3 Light Source Power Supply/Arduino Control 69
11.4 Bundling Cords 69

12 Computer & Software Setup 69
12.1 Overview of Software 69
12.2 ImageJ 70
12.3 MicroManager 70
12.3.1 Overview of Micro-Manager Open Source Project 70
12.3.2 Installing Micro-Manager 70
12.3.3 Configuring Micro-Manager 71
12.3.4 Installing mac-gphoto-enabler 71
12.3.5 Configuring the Camera Using Micro-Manager 72
12.4 Advanced Setup and Customization of Micro-Manager 72
12.4.1 What is source control? 73
12.4.2 Downloads Needed for Editing 73
12.4.3 Setting up the Build Environment to Edit Camera Interface 74
12.4.4 Useful Micro-Manager Debugging Tools: 76
12.5 Implementing Arduino Board for Shutter Control 76

13 Operating the SwingScope 77
13.1 Rotating the SwingScope 77
13.2 Changing Filter Cubes 78
13.3 Adjusting the Focus 79
13.4 Handling and Cleaning 79
13.5 Storage after Imaging 79

14 Imaging Checks & Tests after Installation **80**
14.1 Testing Kohler Illumination 80
14.2 Adjusting Aperture Distance 80

15 Imaging **80**
15.1 Terminology 80
15.2 Imaging Using Micro-Manager 81
15.2.1 Basic use: Taking Images and "Snap" Mode 81
15.2.2 Live Mode 81
15.2.3 Album 81
15.2.4 Multi-dimensional acquisition 82

16 Ellipsometry **82**
16.1 Terminology 82
16.1.1 Setting up for Ellipsometry 84
16.2 Getting Delta and Psi Measurements 85
16.3 Imaging Ellipsometry 86

17 Tubing **86**

18 Troubleshooting **87**
18.1 Dark or Blurred Edges: Alignment 87
18.2 Blurred Stripes: Leveling 87
18.3 Light Source Communication: Arduino 87
18.4 Light Pollution and Leakage 88

1 SwingScope Design

- Off-the-shelf optical components and 3D printed parts
- Open-source software (ImageJ, μManager, Arduino)
- Capable of brightfield, fluorescence, and imaging ellipsometry
- Rotates 180 °around sample for upright, inverted, side-on imaging, and imaging ellipsometry
- Requires less than one day for a student to assemble

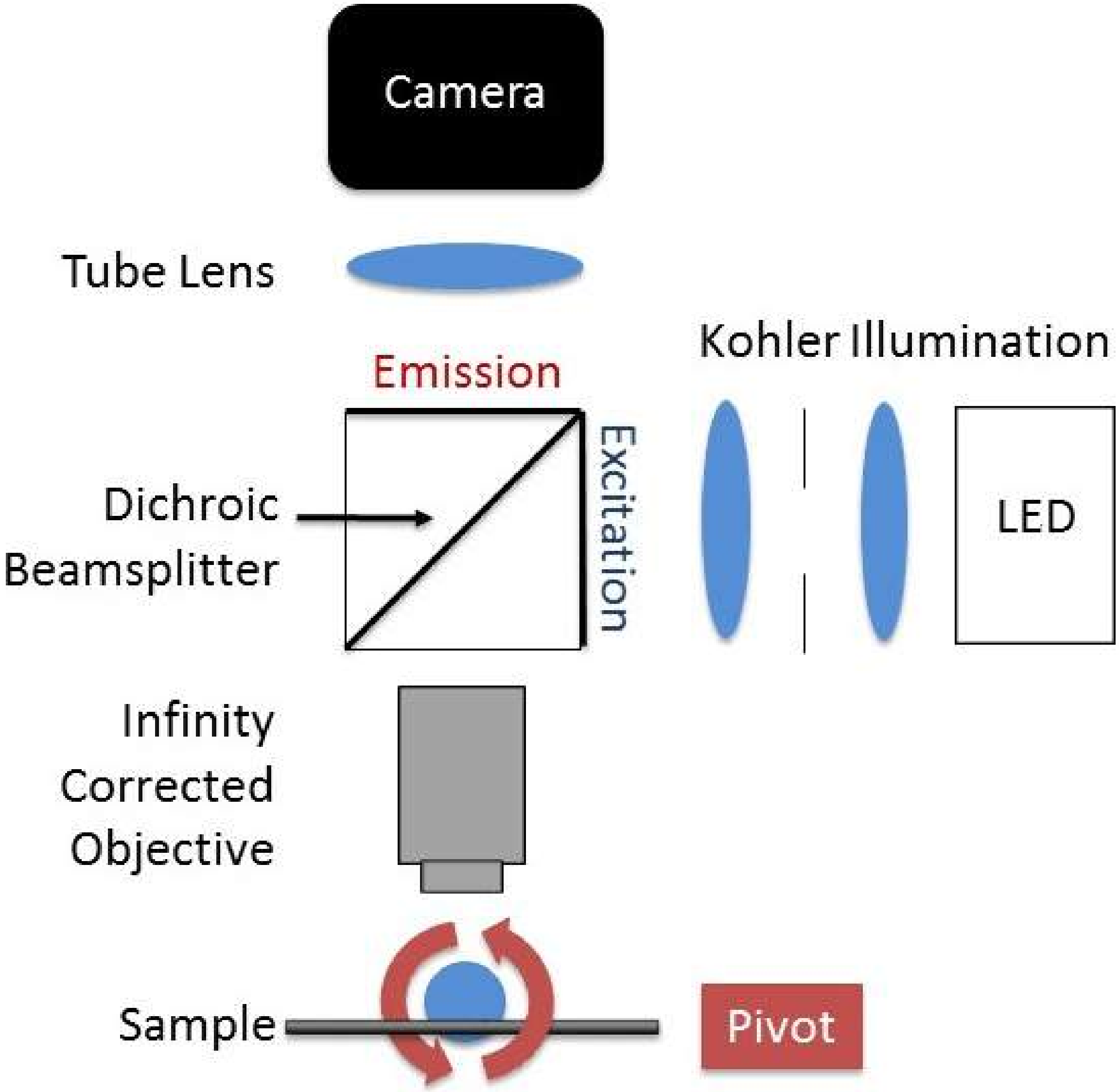

Figure 2: Basic optical layout of the microscope for fluorescence and brightfield

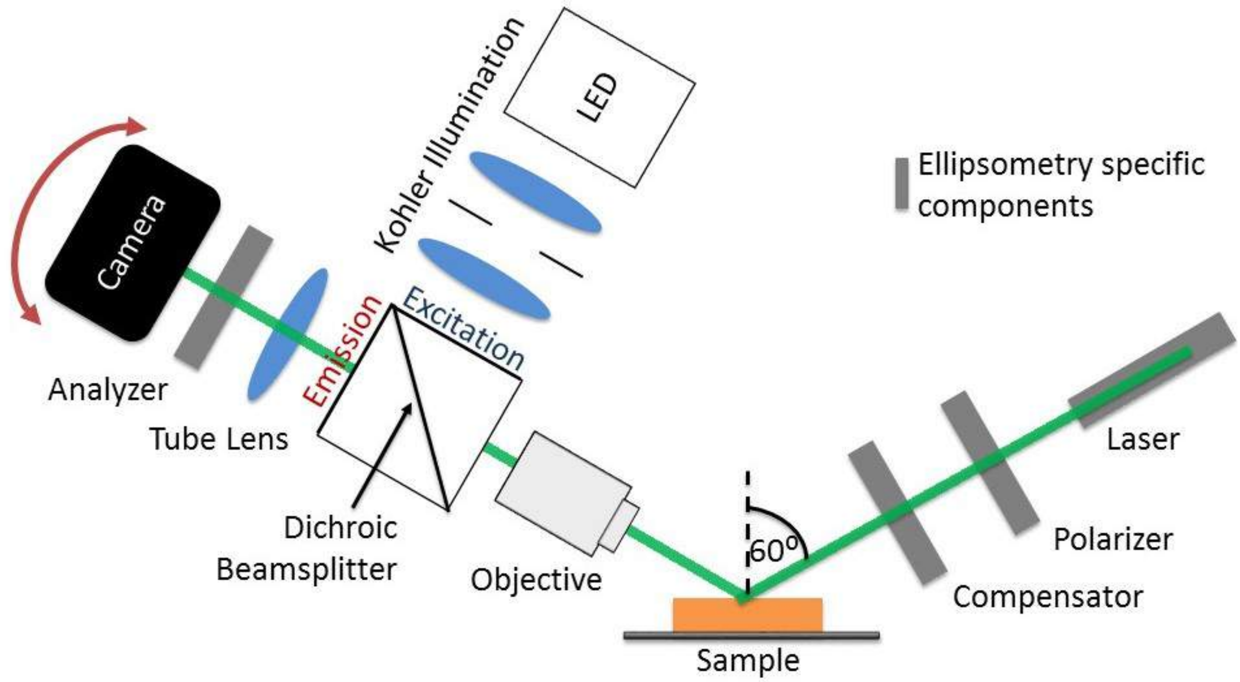

Figure 3: Optical layout for ellipsometry

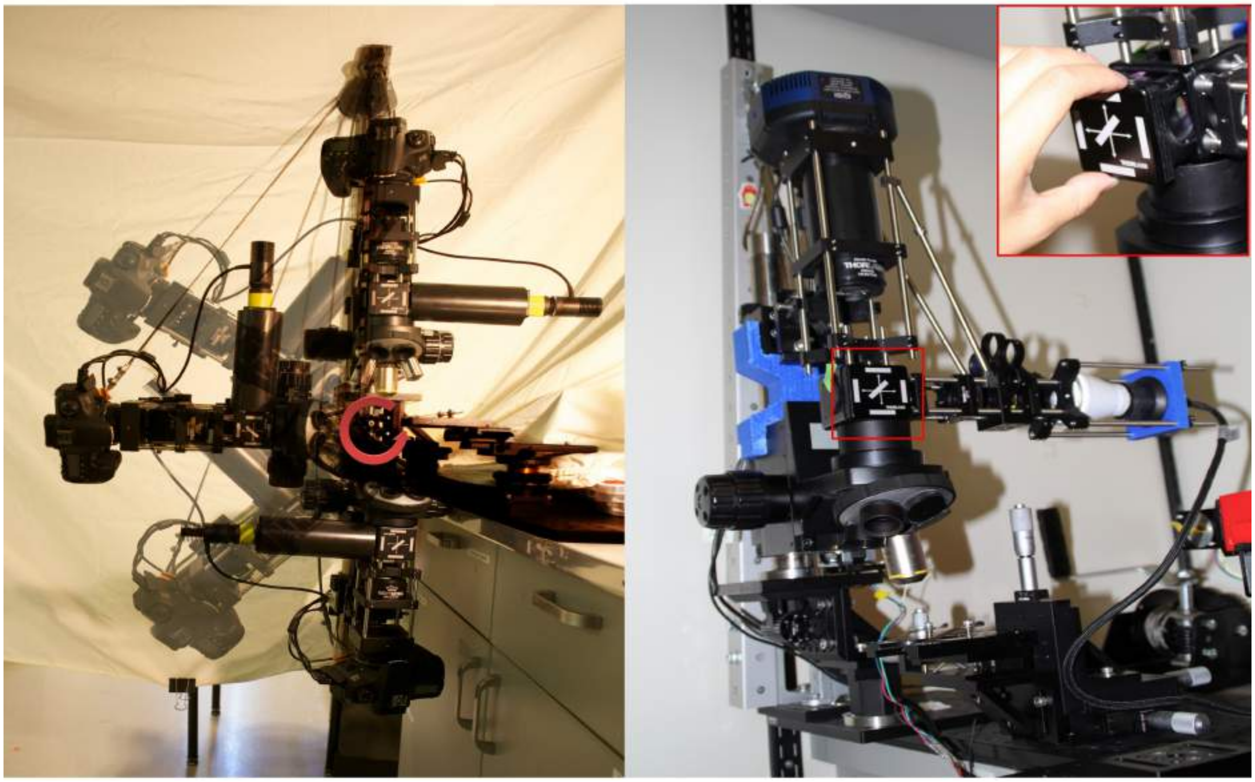

Figure 4: The SwingScope rotates 180 °around the sample and features interchangeable dichroic filter cubes to switch between brightfield and fluorescence capabilities.

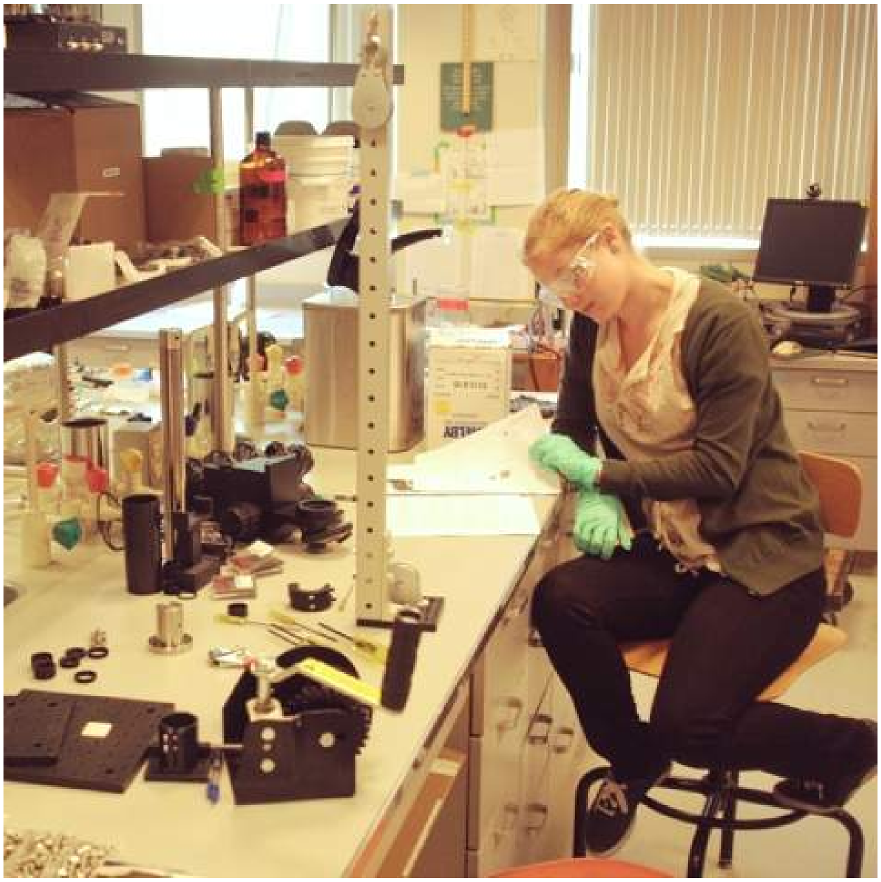

Figure 5: The SwingScope can be assembled from parts and the manual in less than 1 day

2 Characterization

The SwingScope has a 2 μm resolution at 10x (Nikon Plan Fluorite, 0.3 NA). The fluorescence resolution was determined by analyzing the point-spread function of 0.5 μm fluorescent microspheres.

3 Capabilities

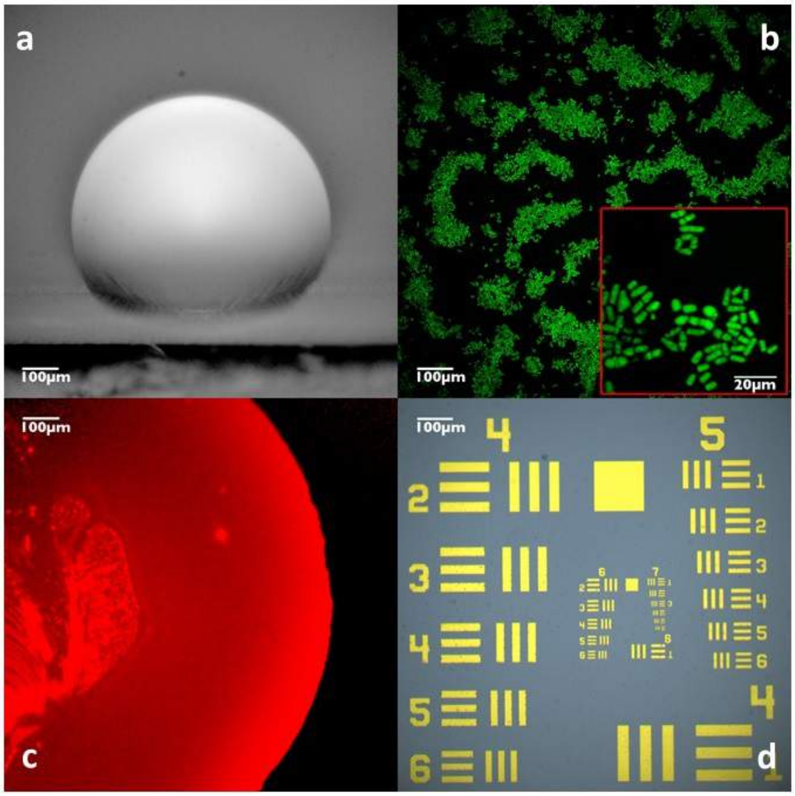

Figure 6: Imaging capabilities. (a) side-on brightfield: water droplet on Teflon at 10x (b) upright fluorescence: distribution of GFP tagged *lid1* mRNA in S. pombe (fission yeast) at 10x and 40x (c) inverted fluorescence: spreading POPC single lipid bilayer with 1% Texas Red at 10x (d) upright brightfield: 1951 USAF resolution target at 10X

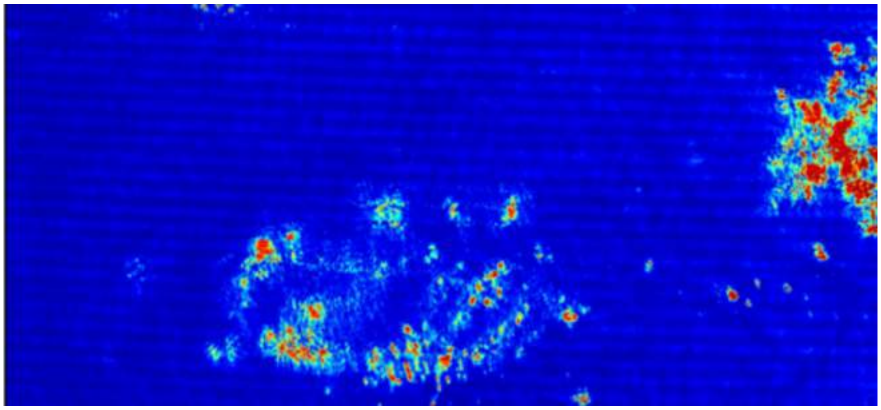

Figure 7: Null-imaging ellipsometry - dust on silicon

4 Costs

Depending on preferences and features, the current cost of the SwingScope is between $14-16k enabling it to meet research needs in a cost effective manner. Traditionally fluorescence imaging is fulfilled by two separate microscopes - one upright and one inverted - that cost approximately $80K per unit. The SwingScope, by having an ability to rotate 180°around the sample, combines these two capabilities for under $15K. In addition, an industrial imaging ellipsometer can cost upwards of $200K. Imaging ellipsometry capabilities can be added to the SwingScope for approximately $1.5K.

Fluorescence/Brightfield Components	Cost (USD)
**Optics**	
Two-channel Dichroic Filters	925
Selection Filters (optional)	650
Lenses	644
10x Objective	826
40x Objective	1081
**Optimechanics**	
Focus Block	2275
Other	4922
**Optielectronics**	
QSI 628 Camera	2995
Consumer LED	25
Other	127
**Software**	
μManager	Free
Arduino	Free
**Total**	14471

Table 1: :Cost of brightfield and fluorescence Components in SwingScope design

.

Ellipsometry Components	Cost(USD)
**Optics**	
Linear Polarizers	260
Wave Plate	235
**Optimechanics**	
Lens Rotation Mounts	237
Rotation Platform	290
Other	258
**Optielectronics**	
532 nm Laser	155
Other	83
**Total**	1517

Table 2: Cost of ellipsometry components

5 Itemized Parts List

***The parts list is also available on a Google document linked here.**

The SwingScope uses off-the-shelf optical components and 3D printed parts (codes included below) whenever possible. The parts listed here are recommended for a microscope that has bright-field, fluorescence, and ellipsometry capabilities and is optimized for use with Texas Red and NBD fluorophores. However, the design can easily be adapted and optimized for specific laboratory needs by changing the camera, objectives, lenses, filters, light source, etc. Some considerations when customizing parts are listed in the next section *5.1 Recommendations for Customizing Parts.*

Part Description	Part number	Cost ea	Quantity	Total
**1000bulbs.com**				
LED	TCP-10P20D50KNFL	$25	1	$25
**Amazon**				
Lock-Tite	Loctite Blue 242 Threadlocker (209728)	$5	1	$5
Adhesive zip tie mounts	Startech Self-adhesive Cable Tie Mounts	$9	1	$9
LED power source	XANTECH AC1 Controlled AC Outlet	$99	1	$99
Black foil	Rosco Matte Black Cinefoil, 12" x 50' Roll	$33	1	$33
Zip ties	Joy Fish 8" Heavy Duty Nylon Cable Ties	$2	1	$2
Digital level	AccuRemote Digital Level	$30	1	$30
**Arduino**				
Ardunio board	Arduino UNO model	$25	1	$25
**Chroma**				
NBD/Texas Red filter set	59022, ET-EGFP/mCherry (FITC/TxRed)	$925	1	$925
NBD selection filter	ET470/40x	$325	1	$325
Texas Red selection filter	ET520LP	$325	1	$325
**Edmund Optics**				
12.5mm linear polarizers	47-215	$130	2	$260
**Ikea**				
Light cord	SEKOND cord set, article: 502.793.73	$4	1	$4

Part Description	Part number	Cost ea	Quantity	Total
**McMaster Carr**				
1/4” thick rubber matting	1310N55, 60A medium hard	$73	1	$73
Sideways pulley	3074T31	$22	1	$22
Upright pulley	3087T41	$16	1	$16
Padlock pulley	3099T49	$18	1	$18
Winch	3205T15	$70	1	$70
Steel cable	3461T38 (10 ft length)	$15	1	$15
S-shaped hook	3784T22	$40	1	$40
Metal pole	4931T123	$21	1	$21
Cable clamp	5513T14	$6	1	$6
Floor-mounting bracket	8809T64	$15	1	$15
**Olympus**				
40x objective	Olympus LUCPlanFLN	$1,081	1	$1081
**QSI**				
QSI 628 camera	Model: 628, SN: 602401	$2,995	1	$2,995
**Shelby Industries**				
6” winch handle	0930-11Z	$15	1	$15
**Thorlabs**				
Lens paper	CP-100	$11	1	$11
Ball-drivers	BD-KIT	$27	1	$27
1/4-20 screws	HW-KIT2	$109	1	$109
Spanner wrench	SPW801	$96	1	$96
1” diameter tubing	SC1L24	$10	1	$10
2” diameter tubing	SC2L24	$12	1	$12
Small 90 degree bracket	AB90A	$26	2	$51
Laser holder	AD11F	$28	1	$28
Rotation platform	AP180	$170	1	$170
90 degree bracket	AP90	$78	3	$234
1/4” spacer	BA2S5	$24	2	$47
1/2” spacer	BA2S6	$26	1	$26
1” spacer	BA2S7	$33	1	$33
2” spacer	BA2S8	$43	1	$43
Brightfield beam splitter	BSW10R	$110	1	$110
Cage grip	C1060	$79	1	$79
C-clamps	C1498	$31	2	$61
Vertical platform	C1519	$96	1	$96
Swivel coupler (4 pack)	C2A	$99	1	$99
Filter wheel	CFW6	$145	1	$145
Cage bracket	CP02B	$13	1	$13
Lens holders	CP06	$17	2	$34
Laser plate	CP6S	$18	1	$18
525nm laser	CPS532	$155	1	$155

Part Description	Part number	Cost ea	Quantity	Total
**Thorlabs (continued)**				
Cage rotation mount for optics	CRM1	$79	3	$237
Mounting post grip	CV1530	$179	1	$179
Filter cube holder	DFM1	$326	2	$652
14" damped mounting post	DP14A	$199	1	$199
0.5" posts	ER05	$5	2	$10
1" posts	ER1	$5	12	$60
2" posts	ER2	$6	2	$12
3" posts	ER3	$6	2	$13
4" post with gradations	ER4E	$16	1	$16
4" posts	ER4-P4	$27	2	$55
6" posts	ER6	$8	6	$51
8" posts	ER8	$11	3	$34
Cage stop	ERCPS	$40	1	$40
Pocket screws	ERSCA	$11	6	$65
Tube lens	ITL200	$450	1	$450
40mm lens	LBF254-040-A	$42	1	$42
75mm lens	LBF254-075-A	$42	1	$42
Tube lens holder	LCP01	$37	1	$37
Thick 60 mm cage plate	LCP01T	$37	1	$37
30-60mm cage adapter	LCP02	$38	2	$76
Laser power supply	LDS5	$83	1	$83
Objective adapters	M25A1	$25	6	$150
Sample holder	MAX3SLH	$124	1	$124
24x12 board	MB1224	$259	1	$259
4x6 plate	MB4	$41	1	$41
4.5x4.5" plate	MB4A	$62	2	$124
6x6 plate	MB6	$48	1	$48
8x8 plate	MB8	$72	1	$72
Focus block	MGZ30	$2,275	1	$2,275
10x objective	N10X-PF	$826	1	$826
1" metal post	P1	$20	1	$20
2" metal post	P2	$26	1	$26
Metal post base	PB1	$24	1	$24
XYZ translation stage	PT3	$867	1	$867
RMSA7 adapter ring	RMSA7	$20	2	$40
Rotating mount	SL20	$290	1	$290
SM1A28 adapter ring	SM1A28	$19	2	$38
Aperture	SM1D12D	$64	1	$64
1" lens tube	SM1L10	$14	1	$14
Tube lens adapter	SM2A20	$45	1	$45
0.5" lens tube	SM2L05	$25	1	$25
SM2 coupler	SM2T2	$34	1	$34
0.5" lens tube	SM3L05	$34	2	$68
XY aperture holder	SPT1	$60	1	$60
T-mount adapter	TMA4	$22	1	$22
Compensator/wave plate	WPMQ05M-532	$235	1	$235

5.1 Recommendations for Customizing Parts

Camera: The current design uses a QSI 628 camera, which is a monochrome charge-coupled device (CCD). It replaced the Canon 60D DSLR camera used in the first prototype because of its increased speed and sensitivity. Depending on imaging needs, a DSLR camera can be a good and affordable option.

Filters: The filters listed in this manual are specifically for use with NBD and Texas Red fluorophores. For filters adapted to the spectra of other fluorophores, browse filters at Chroma.

Objectives: The current design uses a 40X Olympus Plan Fluorite objective and 10X Nikon Plan Fluorite objective, however, objectives can be selected to fit specific imaging needs. When selecting objectives consider the numerical aperture, wavelength of excitation/emission, and correction for aberrations. Places to look: Thorlabs, Olympus, Nikon

LED light source: Consider white LED light sources whose spectral peaks overlap with the spectra of your filters and fluorphores. The current design uses a less expensive consumer LED because it was more efficient at exciting NBD/Texas Red when paired with our filter set in comparison to the industrial Thorlabs LEDs used in our prototype. Places to look: 1000bulbs.com, Thorlabs.

Lenses: Consider the focal length, coatings (like anti-reflection coating), and corrections for chromatic aberrations. Places to look: Thorlabs, Edmund Optics.

6 Codes for 3D Printed Parts

A few 3D printed part were included in the design to align different parts of the microscope and allow customized parts to be added. All of the following parts were created using OpenSCAD software, an Afina H-series 3D printer, and ABS plastic.

6.1 LED Adapter

This part is used to hold the customizable LED.

```
difference(){

cube([71.1+0.5,71.1+0.5,12],center=true);
translate([0,0,-20])
    cylinder(h=50, d=38.1+1.9);
translate([-30,30,-10])
    cylinder(h=50, d=6+0.45);
translate([30,30,-10])
    cylinder(h=50, d=6+0.45);
translate([-30,-30,-10])
    cylinder(h=50, d=6+0.45);
translate([30,-30,-10])
    cylinder(h=50, d=6+0.45);
}
```

6.2 H-shaped Piece

This 3D printed piece is used to align the detection and objective.

```
union(){

translate ([0,0,-38.5])        //Lower bracket, clamps onto focus block
difference(){
    cube([100,49,38.5]);
    translate([10,-5,0])
        cube([80,47,30]);
    translate ([0,42,0])
        cube([100,10,17]);
}

translate([0,0,56])         //Upper bracket, clamps onto mount-cage extension
difference(){
    cube([96.2,42,44.5]);
        translate([10,-5,8.5])
            cube([76.2,50.8,45]);
}

translate([0,21.4,0])    //Middle of H-shaped part
rotate(a=[90,0,0])

union (){
    linear_extrude(height=42, center=true)
    polygon(points = [ [0,0],[100, 0], [48.6,28] ]);

    translate ([0,56,0])
        mirror([0,1,0])
            linear_extrude(height=42, center=true, convexity=10)
            polygon(points = [ [0,0],[96.2, 0], [48.6,28] ]);

    translate ([48.6,32-4,0])
        linear_extrude(height=42, center=true)
        square ([40,56],center = true);
 }
}
```

6.3 3D Printing Instructions

6.3.1 OpenSCAD

1. Copy and paste code into OpenSCAD.
2. Load preview of shape (F5).
3. Compile and render (F6 or Design¿Compile and Render).
4. Export as STL file (Design¿Export as STL).

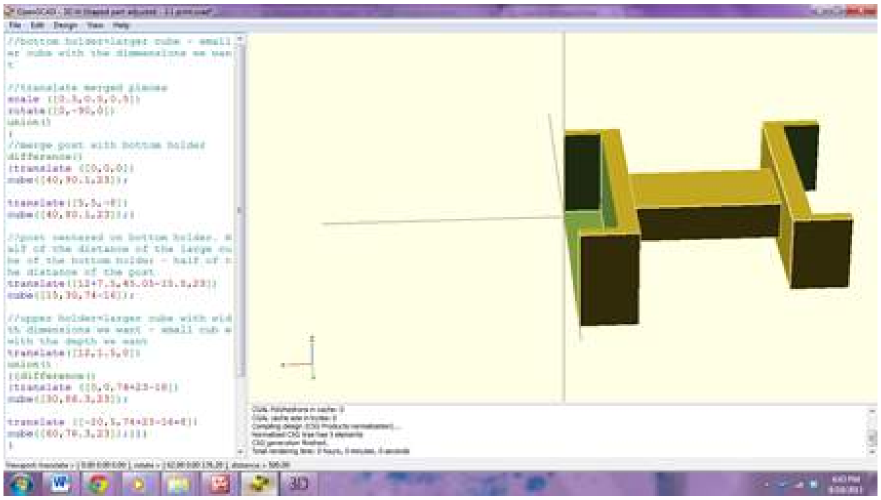

Figure 8: Part in OpenSCAD program

6.3.2 Afina3D Printer

1. Open STL file in Afina 3D.
2. Turn on the 3D Printer. The "on" switch is behind the 3D printer and the light bulb on the front should appear as red.
3. Connect the usb cable to the back of the 3D printer and to your computer.
4. Make sure that the green platform is securely attached to the 3D printer. To do this, use four small clips and attach the green platform to each of the four corners of the black 3D printer platform.
5. Open the Afinia 3D printer software. (This can be downloaded online at http://www.afinia.com/support/dow
6. Before printing can occur, initialize the printer by clicking on the 3D Print drop down menu and clicking initialize. The 3D printer will move to its native position and the light bulb on the front of the printer will turn green.
7. Click on the 3D print drop down menu and select maintenance. Make sure that the nozzle height is set to 134.45 mm.

8. Under maintenance, select table heat for 1 hour. Make sure the platform is heated to at least 100°C before printing.
9. On the right side of the Afinia window select the load option to load your object. This should be a .stl file. (Objects should be created using the OpenSCAD software downloaded here http://www.openscad.org/downloads.html).
10. Select Auto Placement to center your object in the middle of the platform.
11. Click on the 3D print drop down menu and select print. A pop-up box will appear.
12. On the pop-up box, make sure that the platform height is still set to 134.45 mm and select ok. The light bulb should flash green and red quickly as your object is loaded onto the 3D printer.
13. Once the object has been loaded, a second pop-up box will appear stating the mass of the object and how long it will take to complete the print job. Select ok. The 3D printer should begin to print, indicated by a slower green/red flashing of the light bulb.
14. Once the object has been printed use a tool kit to peel off the object from the platform and to remove unwanted material.

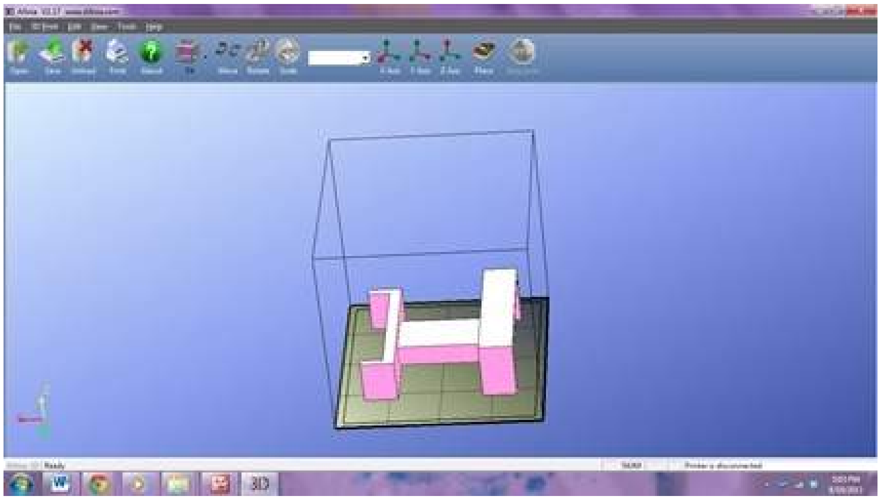

Figure 9: Part in Afina3D as STL file

7 Before You Start Building

7.1 Screws and Threads

All screws used in the assembly procedure are 1/4”-20 screws (1/4” in diameter and contain 20 threads) and are included in the HW-Kit 2 from ThorLabs unless otherwise noted.

- **Threads** refer to the raised spiraling groves on screws and various parts.
- **External threads** match up with internal threads, allowing two pieces to be ”threaded,” or connected together, by applying torque.
- **Countersinks** (or counterbores) are sunken cavities that allow the head of a screw to be below an object’s surface so they do not protrude.
- When threading any two elements together, ensure the threads are properly aligned and use only a few threads at first. If there is resistance, do not force the two elements together as this can damage the threads. Instead unthread, realign, and rethread.
- When multiple screws are involved in connecting two pieces, do not completely tighten the screws until all the screws are in place. Partially tighten each screw, ensuring the threads of the screw turn smoothly, before tightening all the way.

7.2 Cleaning and Handling Optics

These general guidelines have been adapted from ThorLabs’ website.

- Always use gloves when handling all optics or delicate components such as lenses, tube lens, filters, objectives, etc. It may also be advisable to wear gloves that are one size smaller than the size you would normally wear to have better control when handling delicate components.
- Handle coated pieces by the edges only.
- Gently clean optics if necessary. Loose particles should be removed with light, pressurized air. Avoid high velocity air as this can result in dust and other particles scratching the surfaces.
- If necessary, wipe surfaces using anhydrous alcohol, such as pure ethyl alcohol, and lint free towels such as Webril pads designed specifically for optics.
- AVOID wiping surfaces with dry towels. This can cause scratching.
- AVOID touching or wiping A/R coated or metal mirror surfaces.
- AVOID handling exposed coatings with bare fingers.

7.3 Building Tips

- Replace the setscrews that come with the pocket screws with slightly longer ones for ease of access.
- Level posts with respect to a plate/surface by placing the plate on a flat level surface. Slide the posts through so that they contact the surface before tightening the setscrews.
- Use a pencil and the diagrams in the manual to mark the bores that will have a screw threaded into it.

8 Assembly Procedure

8.1 Mounting Post

- 14" Damped mounting post (DP14A)
- 1" metal post (P1)
- 3/4" setscrew (x1)

1. Screw a 3/4" setscrew into the top of the 14" mounting post (DP14A).
2. Thread the 1" metal post (P1) onto the setscrew.
3. Slip the screwdriver through the hole that extends through the 1" metal post and twist to make sure they two metal posts are tightly fitted together.

8.2 Rotation Platform Post

- 2" metal post (P2)
- Metal base (PB1)
- 5/8" screw (x1)
- Lock-Tite

1. Apply Lock-Tite to a 5/8" screw. Pass the screw through the sunken counterbore at the bottom of the metal base (PB1). Use it to connect the metal base to the 2" metal post (P2).
2. Use a hexkey to turn the screw as much as possible for a tight fit.
3. Set this aside to allow the Lock-Tite to cure.

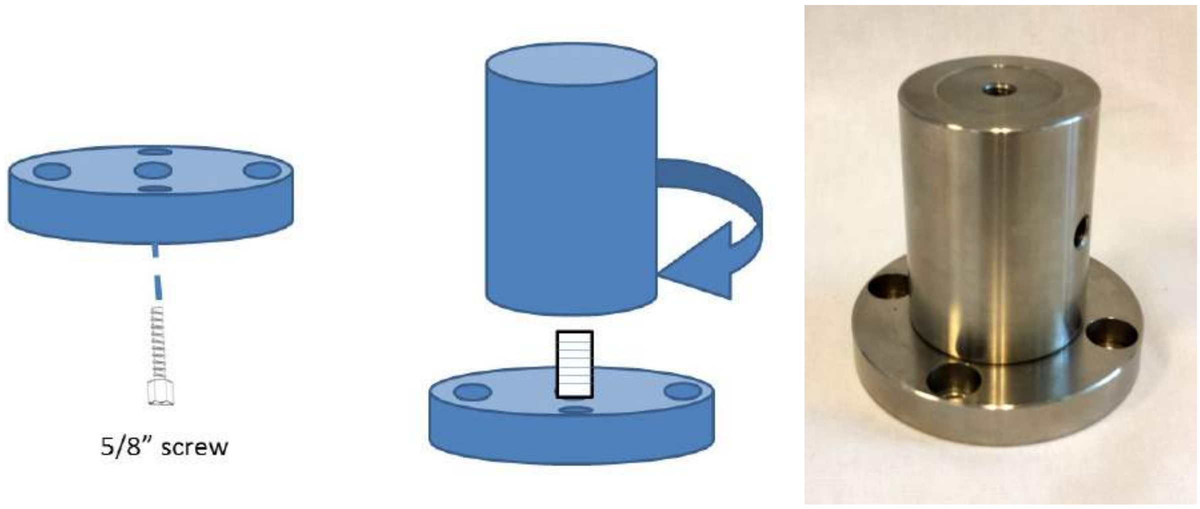

Figure 10

8.3 Mount-to-Cage Extension

Design Note: The CV1530 cage clamp is intended for a 30 mm cage system, but may be modified for a 60 mm cage system and 1.5" mounting post. Each clamp consists of two main parts: a part that grips the mounting post and a part that grips the cage rods. These two elements may be disassembled and additional spacers of various lengths added to properly center the cage system over the sample.

Parts:

- Mounting post grip (CV1530), parts 1 and 2
- Cage grip (C1060), part 2a
- 2" spacer (BA2S8)
- 1/2" spacer (BA2S6)
- 1/4" spacer (BA2S5)
- 2" screws (x4)
- 1.5 " screws (x4)
- 1" screws (x2)
- Washers (x4)
- Nuts (x4)

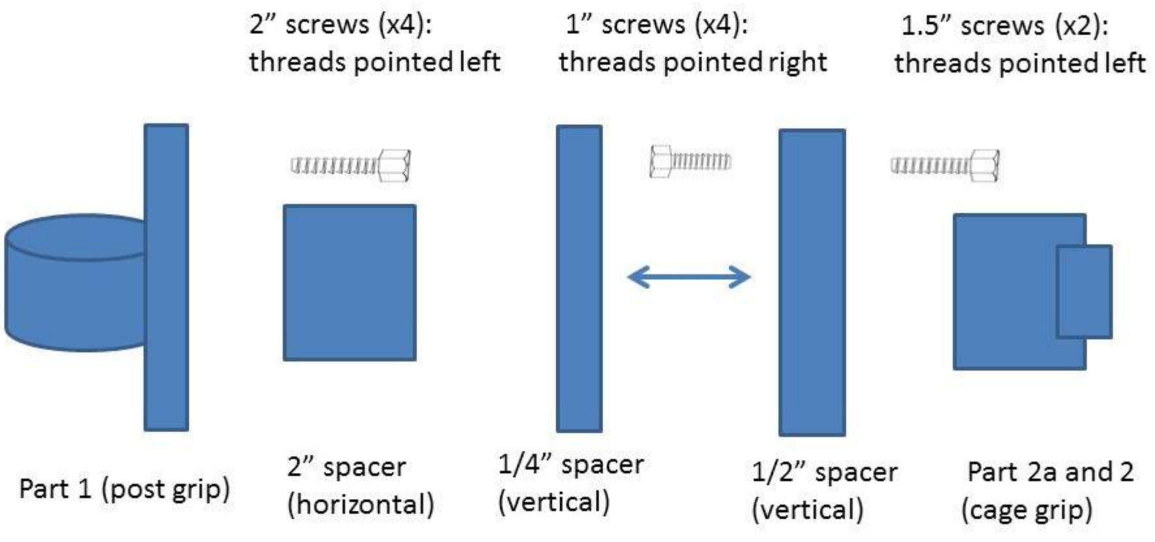

Figure 11: Schematic of spacers and screws in assembling Mount-to-Cage Extension

1. For part CV1530: Separate the clamp into its three parts. Parts 1 and 2 will need to be retained.

2. For part C1060: Separate the clamp into its two parts. Part 2a, including the four screws that held it in place, will be needed in the following steps.

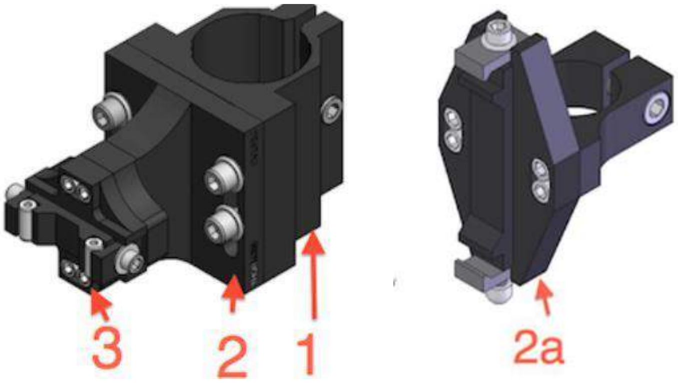

Figure 12: Step 1 (left) and Step 2 (right)

3. Orient the 2" spacer (BA2S8) with respect to Part 1 so that the side of the spacer with the printed height is furthest away from Part 1.

4. Align the spacer's clearance slots with the holes on Part 1.

5. Loosely thread two 2" screws through each of the riser's clearance slots on the countersunk side where the height is printed. Slide the spacer until the edge of the clearance slot nearest the printed height is flush with the screws before tightening completely.

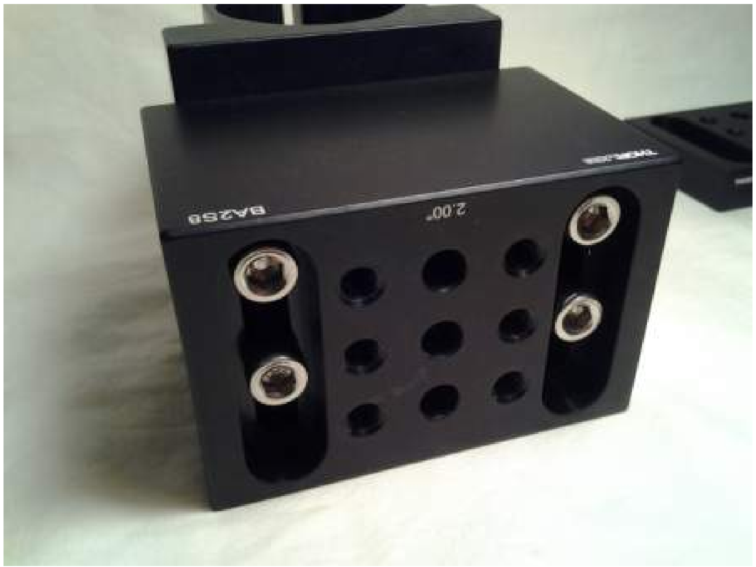

Figure 13: 2" spacer connected to Part 1 (steps 3-5)

6. Slide two 1.5" screws through each clearance slot of the 1/2" spacer (BA2S6) on the countersunk/printed side.

7. Stack the 1/4” spacer (BA2S50) behind the 1/2” spacer so the screw head is between the two spacers.

8. Orient the stack vertically to the 2” spacer so the clearance slots of the stack and that of the 2” spacer are perpendicular with respect to one another. The screw threads should point away from the 2” spacer.

9. Connect the stack to the 2” riser by threading 3/4” screws (x2) through the countersunk bores located at the edge of the middle row of bores in the 1/2”spacer. Note: Before completely tightening all screws, make sure the risers are not tilted and are even with respect to one another.

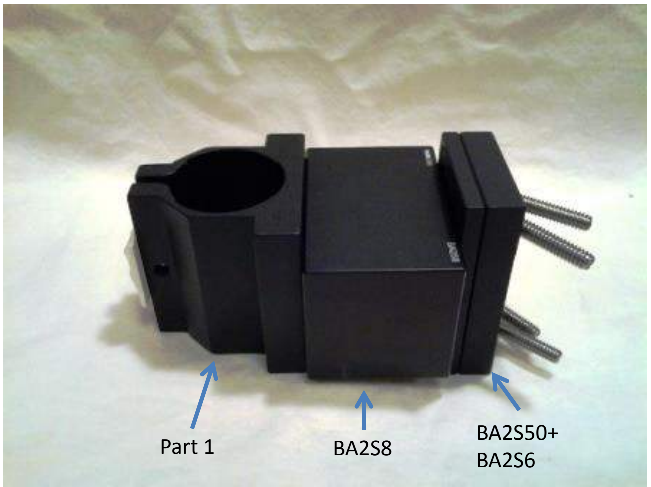

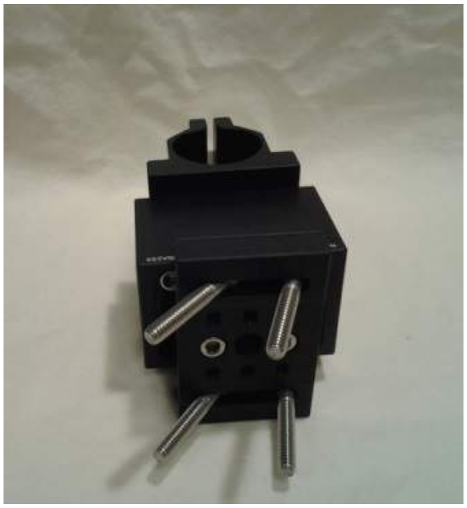

Figure 14: Setup by the end of step 9

10. Orient Part 2a and Part 2 (retained from steps 1 and 2), so that Part 2a is on top and parallel to the direction of clearance slots in Part 2. Make sure the two parts are not tilted with respect to one another before tightening the four small screws to connect them.

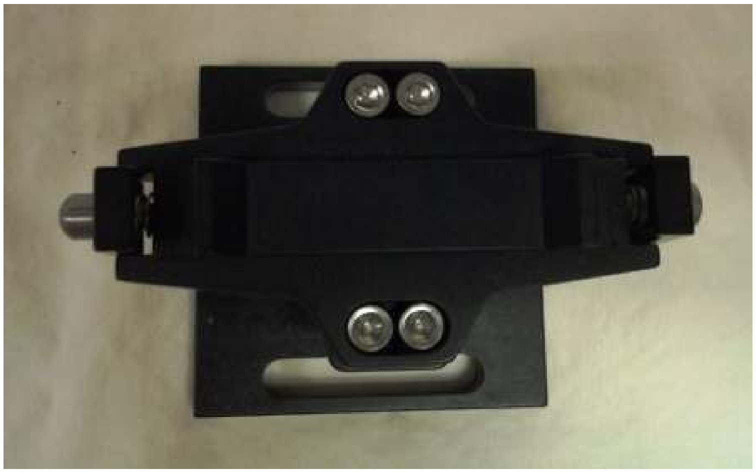

Figure 15: Step 10

11. Slide the clearance slots of the assembled part from Step 10 onto the protruding threads of the 1/2” riser from Step 9.

12. Adjust the screw threads so they are at opposite ends and as far apart as possible. Center the assembled part from Step 10 on the threads.

13. Slide washers (x4) onto the threads and then use nuts (x4) to fasten the assembled part from step 7 to the other risers (see Figure 16).

14. Use a small hex screwdriver that fits through the opening to keep the 2” screw from rotating while using a wrench to tighten each nut (see Figure 16).

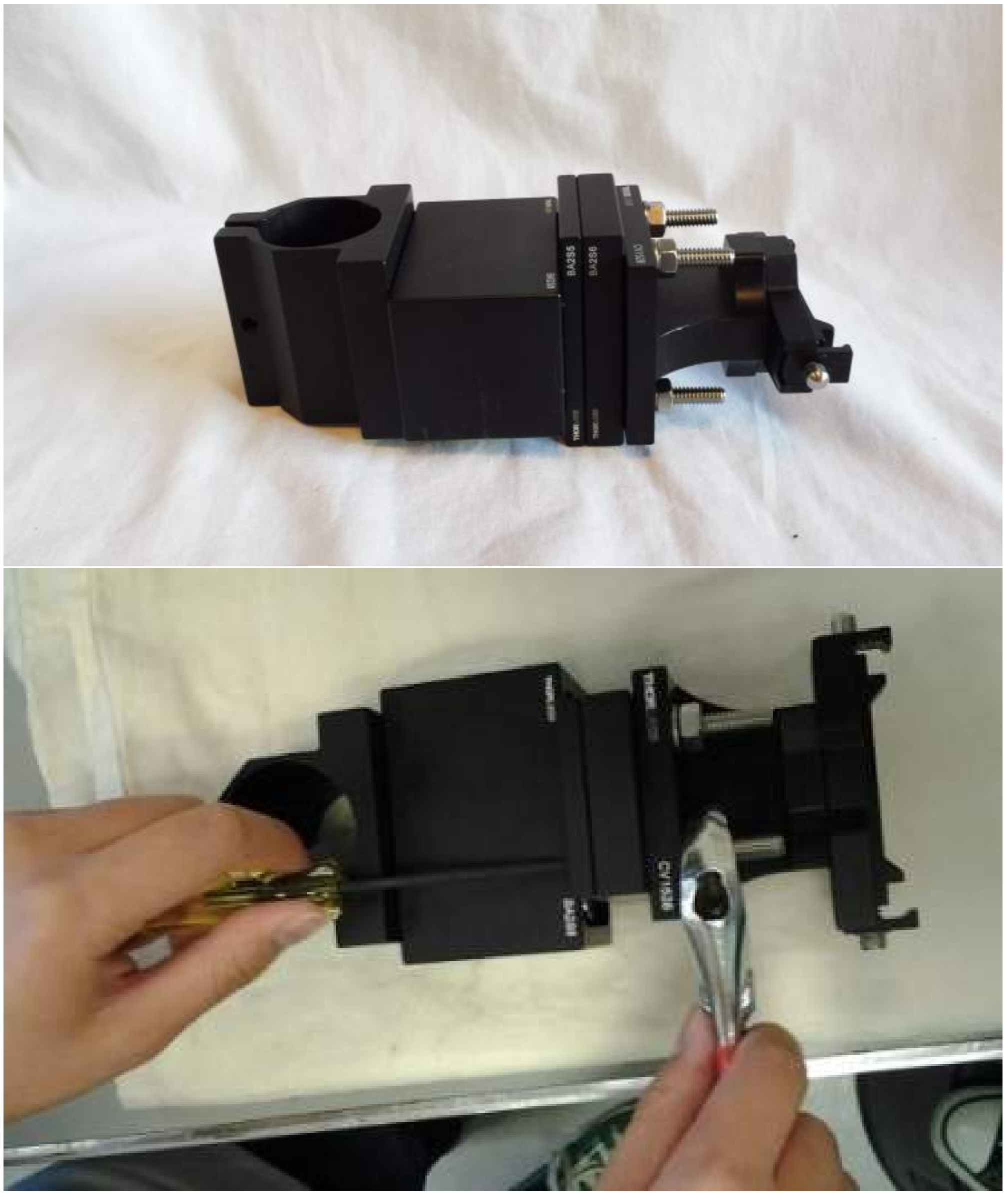

Figure 16: Steps 13 and 14

8.4 Sample Stage

Parts:

- XYZ translation stage (PT3)
- Sample holder (MAX3SLH)
- 90 degree bracket (AP90)
- 1/4” spacer (BA2S5)
- 1” screw (x1)
- 2” screw (x1)
- 5/8” screw (x2)
- Nut (x1)

1. Set all axes of the translation stage (PT3) to half their total travel distance at the ”5” marking.
2. Attach the 90 degree bracket (AP90) to the Z axes of the translation stage using two 5/8” screws. The shorter side of the bracket should attach to the face of the Z axis so that the bottom of the bracket is in line with the bottom of the Y axis stage.
3. Connect the sample holder, 1/4” spacer, and the 90 degree bracket:
    (a) Pass a 1” screw through the clearance slot of the sample holder, the middle thread of the 1/4” spacer, and topmost thread of the 90 degree bracket
    (b) Pass a 2” screw below the thread in step above.
    (c) Use a nut on the opposite side to fasten. Tighten using a screwdriver and wrench.
    (d) Adjust the spacer, bracket, and sample holder so the edges are parallel before tightening

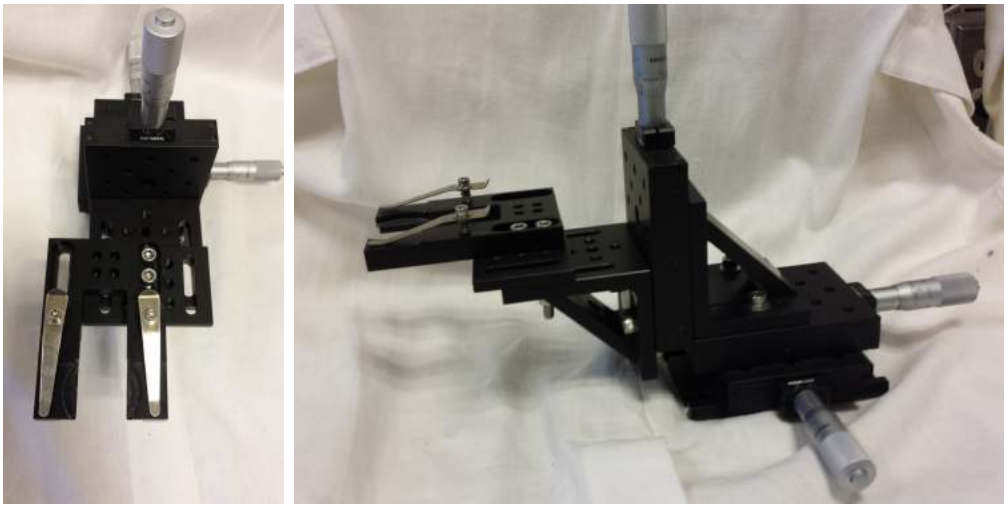

Figure 17: XYZ Sample Stage

8.5 Rotation Platform

Parts:

- Rotation platform (AP180)
- 90 degree bracket (AP90)
- Adapter plate (MB4A)
- 3/4" screws (x2)
- 1" screws (x4)
- 3/8" screws (x4)
- Washers (x8)
- Nuts (x4)

1. Remove the linchpin from the rotation platform (AP180).
2. Align the side of the 90 degree bracket (AP90) containing four clearance slots with the base of the rotating platform (AP180) that has 4 screws.

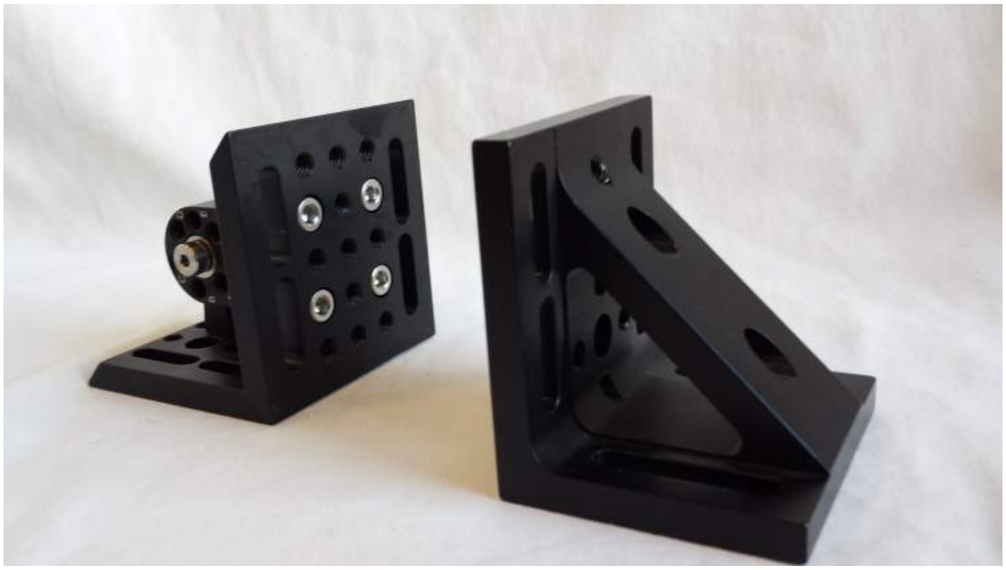

Figure 18: rotation bracket (left) and 90 degree bracket (right)

3. Connect the 90 degree bracket to the rotation platform base using 3/4" screws (x2) threaded through the two bores in the triangle/slanted leg of the 90 degree bracket. Check that the platform and bracket are level by placing both on a flat surface before tightening the screws.
4. Slide a washer onto each 1" screw (x4) and pass the screw through each of the aligned clearance slot with threads pointing towards the rotating platform. Allow the screws to rest on the bottom of each slot.

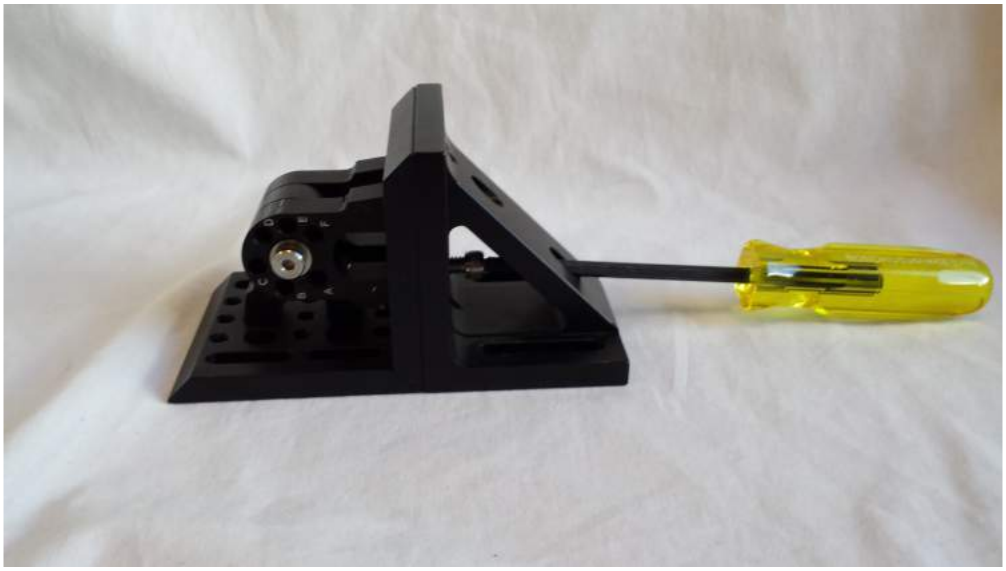

Figure 19: Step 3. screws threaded through the triangular leg of 90 degree bracket

5. Use a washer and nut on the protruding threads of each screw to keep the bracket and rotating platform flush to one another. There should be washers on both sides of the screw.

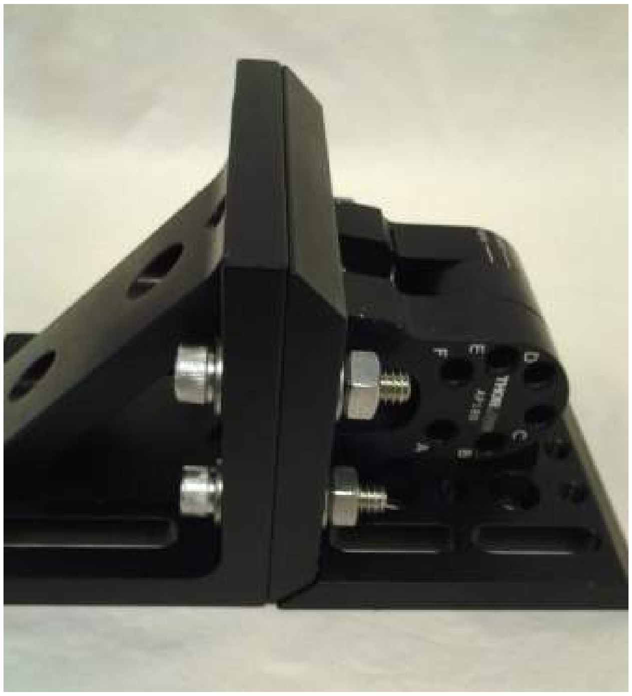

Figure 20: Step 5

6. Use a screwdriver and wrench to tighten each nut. Move the rotation platform around as needed during the process to access each nut.
7. Set rotation platform at 180 degrees (marking F on the side without Thorlabs logo) so that it can be placed on a flat surface.

8. Attach the 4.5x4.5" adapter plate (MB4A) to the rotating platform surface (side with two screws) with 3/8" screws (x4) (see Figure 21 for placement).

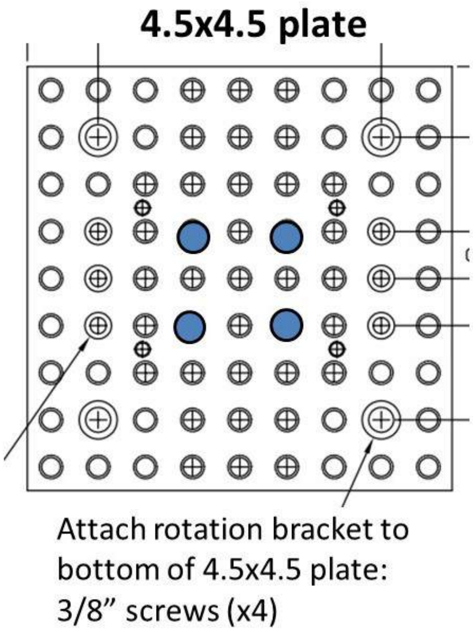

Figure 21: The bores outlined in blue are used to connect the adapter plate to the rotation platform (step 8)

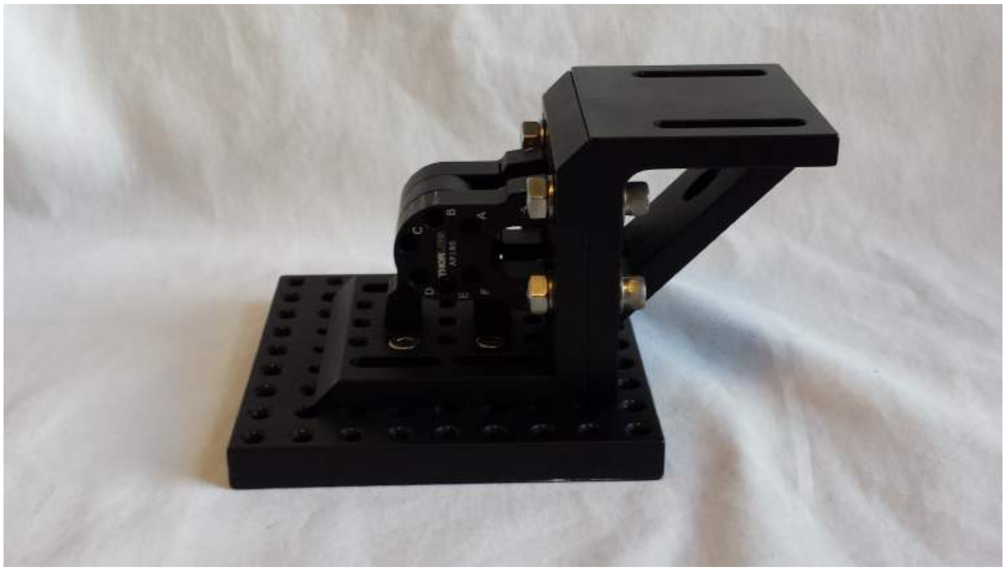

Figure 22: The finished rotation platform

8.6 Kohler Illumination Arm

8.6.1 LED Light Source

Parts:

- LED adapter (3D printed)
- Ikea light cord
- LED (TCP5000)

1. Remove the dome that covers the plug of the IKEA light cord.
2. Thread the IKEA light cord into the 3D printed LED adapter.
3. Thread the LED (TCP5000) into the IKEA light cord.

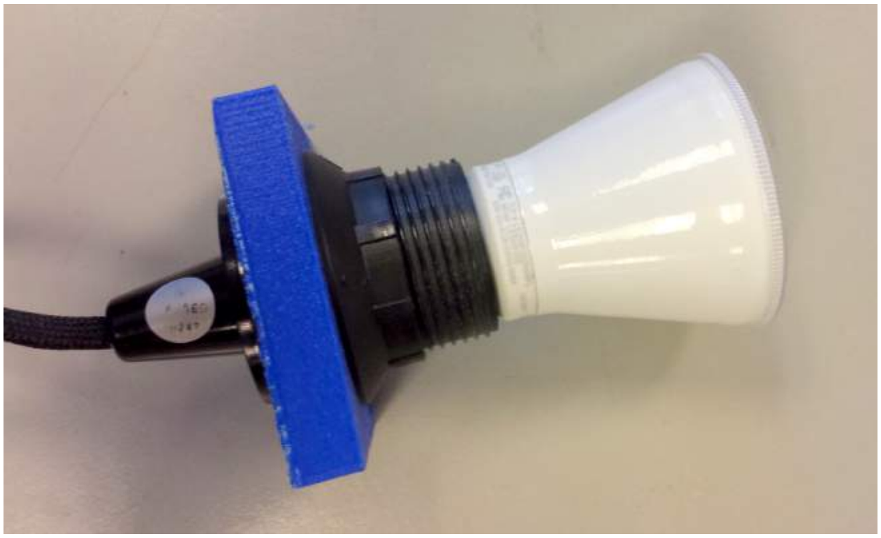

Figure 23: Steps 1-3

8.6.2 Filter Wheel (Optional)

Note: This is an **optional** fluorescence feature. A filter wheel is useful if working with different fluorophores and can be equipped with filters to selectively excite a fluorophore when paired with the filter cube. The SwingScope in our lab uses filters for NBD and Texas Red dye, but the filter wheel can be customized to use with other filters.

- Filter wheel (CFW6)
- Chroma NBD filter (ET470/40x)
- Chroma TR filter (ET520LPDFM1)
- Other filters as needed

Caution: The next few steps involve adding optics and should be done with care while wearing gloves.

1. Use the spanner wrench to remove the threaded retaining rings from the desired filter holder.
2. Place the lens into the desired filter holder, with the arrow/carret on the chroma filters pointing towards the TOP of the numbers on the wheel.
3. Use the spanner wrench to thread the retaining ring into the holder until it is flush with the back of the filter.

8.6.3 Illumination Arm

Caution: The next few steps involve adding optics and should be done with care while wearing gloves.

Parts:

- LED light source (assembled in 8.6.1)
- Filter wheel (assembled in 8.6.2)
- Lens holders (CP06) (x2)
- 30-60mm adapter (LCP02)
- 40 mm lens (LBF254-040-A)
- 75 mm lens (LBF254-075-A)
- X-Y aperture holder (SPT1)
- Aperture (SM1D12D)
- Swivel coupler (C2A) (x2)
- Cage stop (ERCPS) (x4)
- 6” posts (ER6) (x6)
- 4” posts (ER4) (x2)
- 2” posts (ER2) (x2)
- 3” posts (ER3) (x2)
- 0.5” posts (ER05) (x2)

1. Loosen the set screws in each of the lens holders (CP06).
2. While wearing gloves, carefully place the 40mm lens into the holder. Tighten the set screw to keep the lens in place. Place a labeled piece of tape with the lens distance (40mm) onto the side of the lens holder.
3. Repeat step 2 for the 75mm lens.

4. Locate the two setscrews on the front plate of the x-y aperture holder (SPT1). Remove the setscrews and thread them onto the back plate (plate furthest from the internal threads and the side closest to the ThorLabs logo).

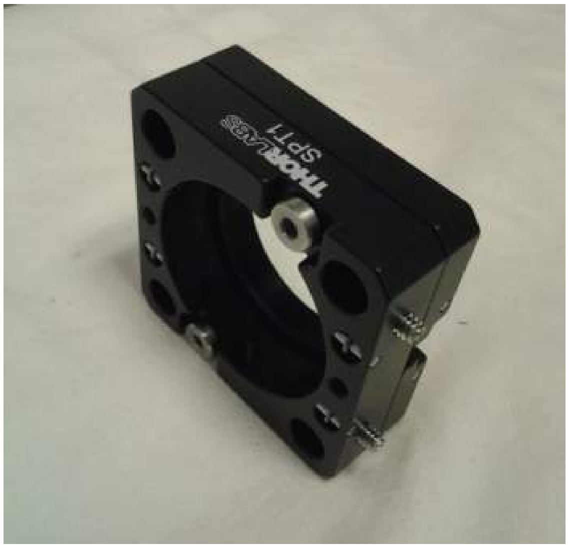

Figure 24: Step 4, with setscrews threaded onto the back plate

5. Thread the aperture (SM1D12D) into the aperture holder. Make sure it is fully open while threading to avoid accidentally damaging the metal that forms the opening.

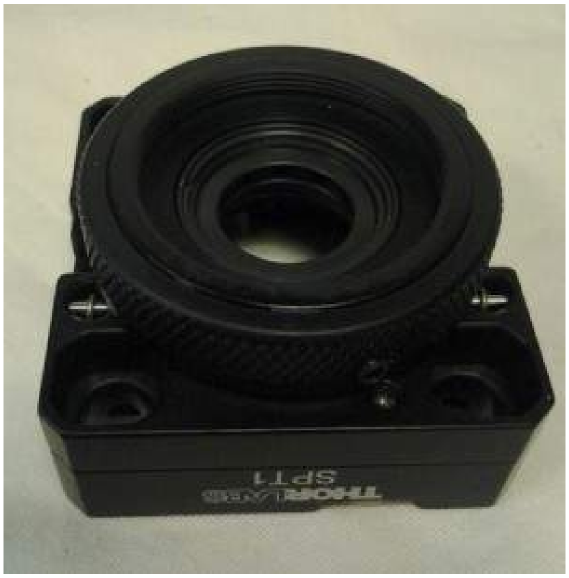

Figure 25: Step 5

6. Locate the inner set of holes (closest to the center) of the 30mm-60mm adapter plate (LCP02). Slide a 2” post into two adjacent inner holes.

7. Slide a 6” post into the other two inner holes.

8. Level the posts with respect to the adapter plate by placing the adapter plate on a flat surface. Tighten the setscrews on the side of the adapter plate.

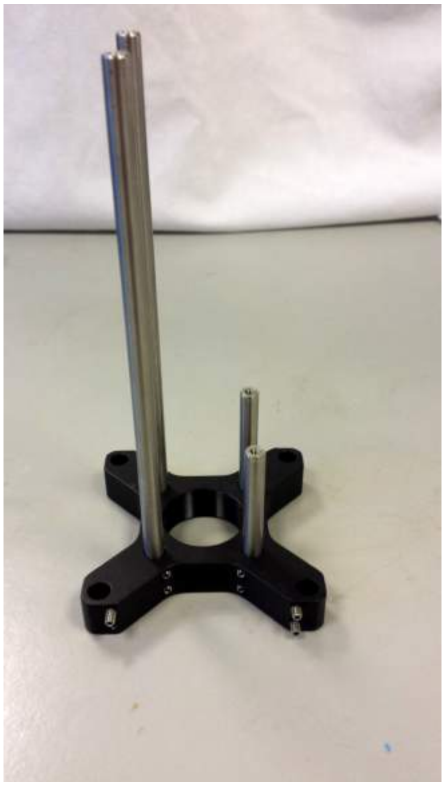

Figure 26: Steps 6-8

9. Slide a 6” post into each of the remaining outer holes of the adapter plate. Level the posts with respect to the other side of the adapter plate so that the posts are on the opposite side of the posts in steps 6 and 7. Tighten the setscrews.
10. Slide the 40mm lens onto the 2” posts with the convex side of the lens facing the adapter plate. Adjust until the lens holder is 21mm from the adapter plate before tightening the setscrews.

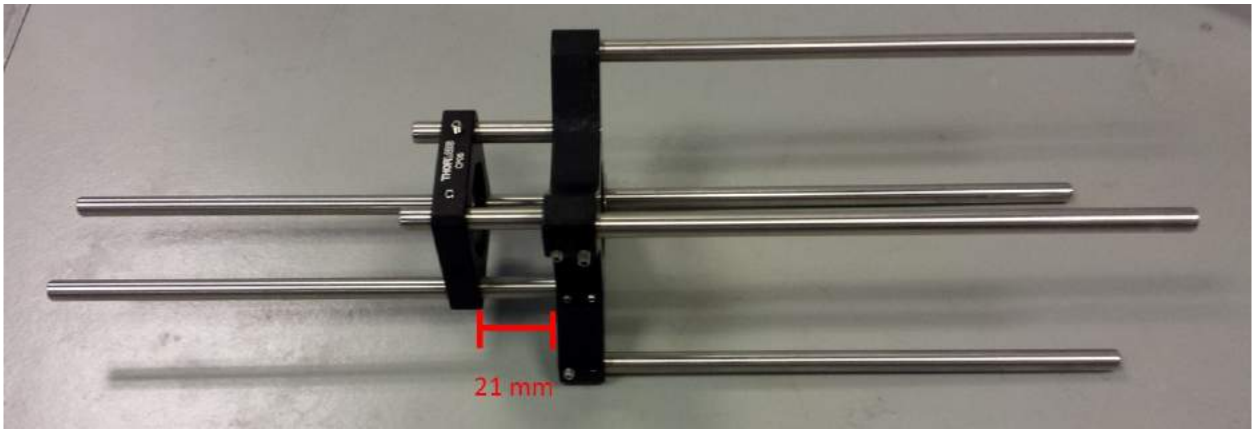

Figure 27: Steps 9-10

11. Slide the filter wheel (group assembled in section *8.6.2 Filter Wheel*) onto the posts so that the top of the numbers on the filter wheel point away from adapter plate. Adjust the filter wheel so that it is flush with the 40mm lens. Tighten setscrews.
12. Create two 3.5" posts by threading a 3" and 0.5" post together.
13. Slide a 3.5" post into the top holes of the filter wheel and tighten the setscrews. **CAUTION: The posts should not go all the way through. Be careful to not damage any filters!**
14. Thread a 4" post onto each swivel coupler (C2A).
15. Slide the swivel coupler onto the posts so that it is flush with the filter wheel. Lock one of the bottom screws to hold it in place.
16. Slide the aperture onto the posts with the aperture pointed towards the filter wheel.
17. Slide the 75mm lens onto the posts with the convex side towards the adapter plate.
18. Adjust the aperture so that is 22mm from the filter wheel before tightening the setscrews (see Figure 28).
19. Adjust the 75mm lens so that it is 7mm from the end of the posts before tightening the setscrews (see Figure 28).
20. Slide the LED adapter (the 3D printed component of the group assembled in section *8.6.1 LED light source*) onto the 6" posts that point away from the adapter plate/lenses. Adjust the LED adapter until the LED is flush with the adapter plate.
21. Slide one cage stop onto the back of each post so that it is flush with the back of LED adapter. Tighten the setscrew on each cage stop.

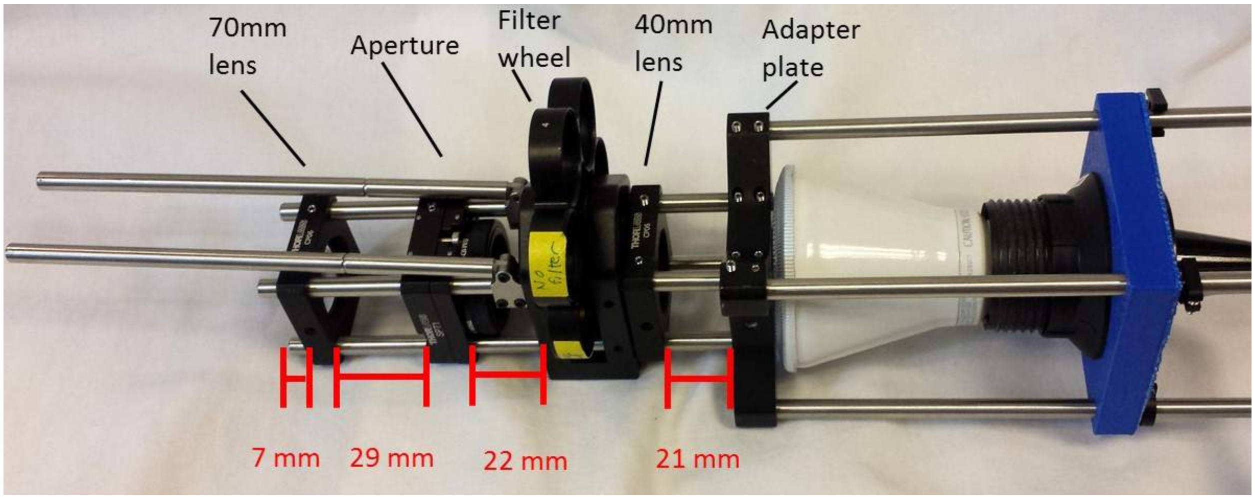

Figure 28: Finished Kohler illumination arm

8.7 Upright 60mm Cage System

8.7.1 Cube to Camera

Parts:

- Filter cube holder (DFM1)
- 30mm-60 mm cage adapter (LCP02)
- Tube lens holder (LCP01)
- T-mount adapter (TMA4)
- SM2 coupler (SM2T2)
- Thick 60mm cage plate (LCP01T)
- Pocket screw (ERSCA) (x6)
- Swivel coupler (C2A) (x2)
- 1” posts (ER1) (x12)
- 8” posts (ER8) (x3)
- 4” post (ER4) (x1)
- 4” post with gradations (ER4E) (x1)
- 0.5” lens tube (SM2L05)
- 1” lens tube (SM1L10)

1. Remove the top from the filter cube holder (DFM1).
2. Locate the face of the filter cube holder opposite the face with a protruding circle. Thread a pocket screw (ERSCA) into each corner of the face.
3. Remove a setscrew from one side of the 1” posts (x8).
4. Thread a 1” post (from step 3) into each corner on top of the filter cube.
5. Slide the inner set of holes of 30mm-60mm cage adapter plate (LCP02) onto the 1” post. Level the posts with respect to the face of the adapter by inverting the system built so far and place the adapter on a flat, level surface. Make sure the posts contact the flat surface before tightening the setscrews.
6. **For ellipsometry capabilities:** Thread a 1” post into the inner set of holes in the adapter plate. These are used to hold the polarizers for ellipsometry.

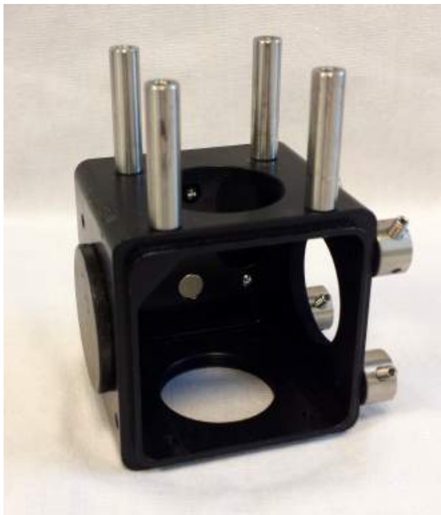

Figure 29: Dichroic filter cube with pockets and 1” posts installed (steps 1-4)

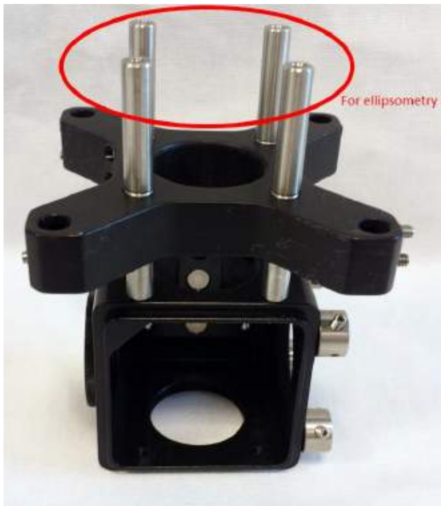

Figure 30: Steps 5-6, with optional posts for ellipsometry capabilities

7. Thread the 1” lens casing (SM1L10) into the bottom of the filter cube.
8. Create four 9” posts by threading the following posts together in this order:
   (a) Make 1 of these: 4” graduated (ER4E)+ 4” + 1”
   (b) Make 3 of these: 8” + 1”
9. Remove the setscrews from both sides of each 9” post.
10. Take the 9” post created in 8a and slide the end with the graduated markings into the corner of the adapter plate such that the graduated marks can be seen if you are looking at the removable top of the filter cube.
11. Slide the remaining 9” posts into the remaining outer corners of the adapter plate.

12. Level the posts with respect to the surface of the adapter plate before tightening the setscrews. This can be done by inverting the system and placing the posts on a flat level surface. If they are properly level the system should be able to stand on the posts without rocking.

13. Thread the 0.5" lens tube (SM2L05) into the tube lens holder (LCP01).

14. Slide the tube lens holder (step 13) onto the posts so that the tube lens holder points away from the filter cube.

15. Designate and tighten 2 setscrews on the tube lens holder that are diagonal from each other. These setscrews will keep the tube lens holder in place during the adjustment process later in the procedure.

16. Thread pocket screws (ERSCA) onto the end of each swivel coupler (C2A).

17. Slide the swivel couplers onto the two posts that are on the same side as the pocket screws on the filter cube. For ease of access, slide the couplers so that the screws on the hinge are facing outward. Tighten the screws to lock the swivel couplers in place.

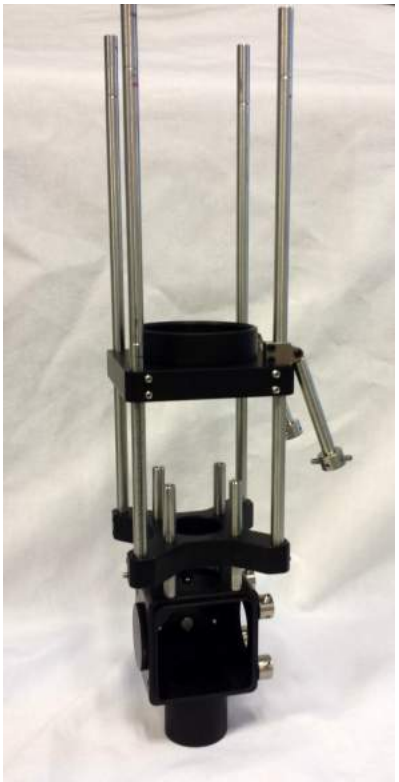

Figure 31: Set up after steps 7-17

18. Thread the SM2 coupler (SM2T2) into the T-mount adapter (TMA4). This will be used to mount the camera.

19. Thread the part in step 15 into the thick 60mm cage plate (LCP01T).

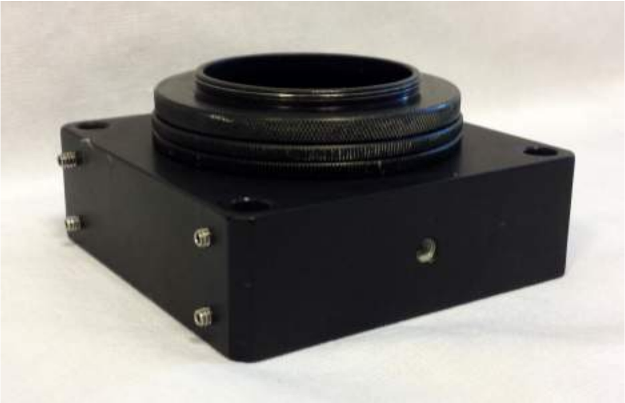

Figure 32

20. Slide the thick cage plate onto the metal posts, with the T-mount adapter pointed away from the filter cube. Adjust until there is 8mm between the end of the posts and the top of the thick cage plate. **Note:** Use a sharpie to mark the post with the position of the plate in case of future adjustments.
21. Place the cage system built thus far onto a flat surface. Use a leveling tool to make sure the top of the T-mount adapter where the camera should be connected and the flat surface are the same level.

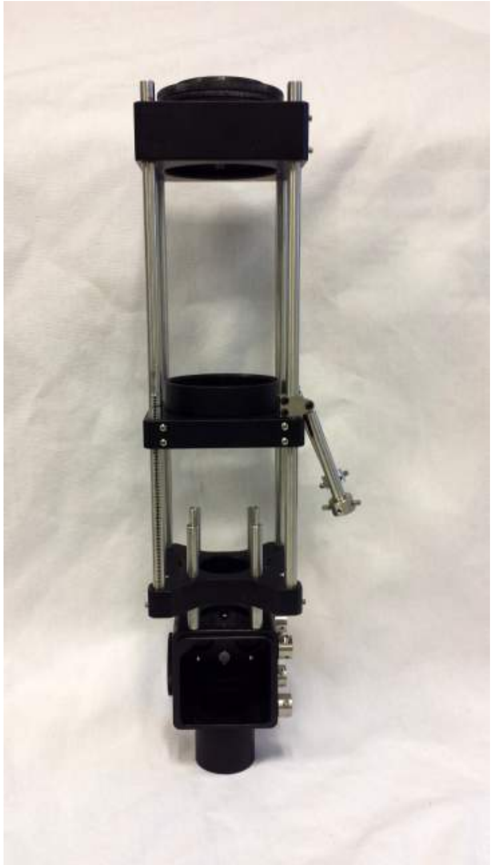

Figure 33: Steps 20-21

8.7.2 Adding Tube Lens

Caution: The next few steps involve adding optics and should be done with care while wearing gloves.

Parts:

- Tube Lens (ITL200)
- Tube Lens Adapter (SM2A20)

1. While wearing gloves, thread the tube lens (ITL200) into the tube lens adapter (SM2A20).
2. Make sure the tube lens holder (LCP01, in the previous procedure *8.7.1 Cube to Camera*) is secure before proceeding.
3. Invert the assembled upright cage system so that the filter cube is pointing upwards and then place the system on a flat surface.
4. Carefully loosen the setscrews keeping the lens holder in place. Slide the lens holder down to make room to thread tube lens in.
5. Carefully slide the tube lens between the posts and thread it into the tube lens holder so that the "objective" label points towards the filter cube.

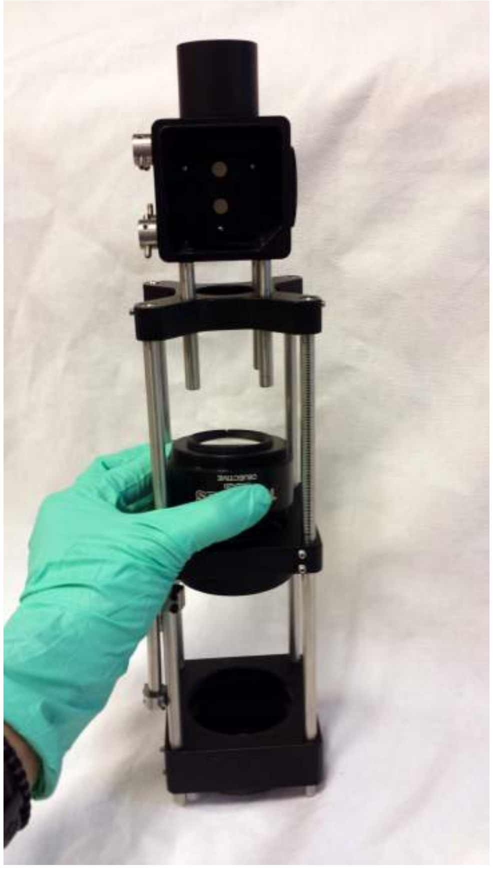

Figure 34: Installing the tube lens

6. Turn the system onto its side. Adjust the tube lens holder so the top of the tube lens holder is 5 notches from the top end of the graduated post **Tip:** mark this position on the post with a sharpie for future use. This ensures the tube lens is at the correct distance from the camera's image plane.

7. Tighten all the setscrews to keep the holder in place.

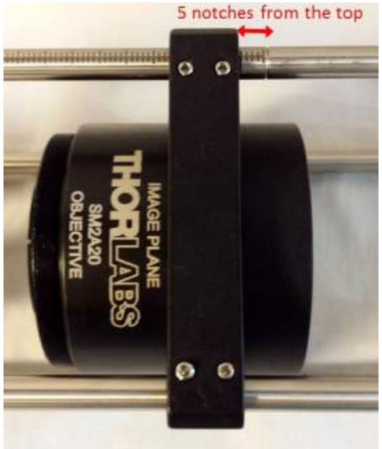

Figure 35: Step 6

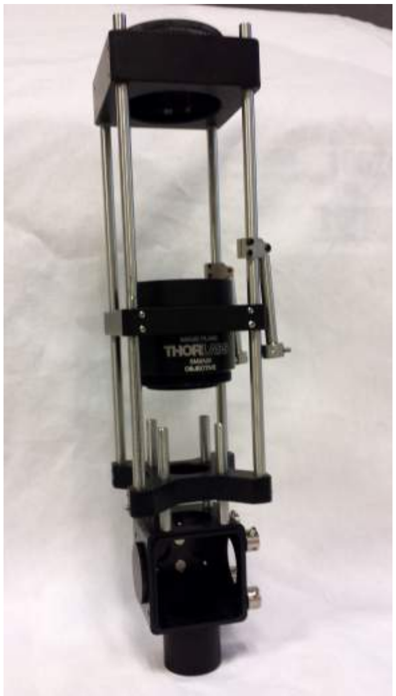

Figure 36: Upright cage system after the tube lens has been added

Note: Future handling and removal of the tube lens (such as for cleaning) should be done with care. Avoid bumping into other elements that could damage the lens.

8.8 Filter Cubes

8.8.1 Brightfield Filter Cube

Caution: The next few steps involve adding optics and should be done with care while wearing gloves.

Parts:

- Beam splitter (BSW10R)
- Dichroic filter cube (DFM1)

Drawings from Thorlabs of Dichroic filter cube for reference

1. Remove the top from the filter cube base.

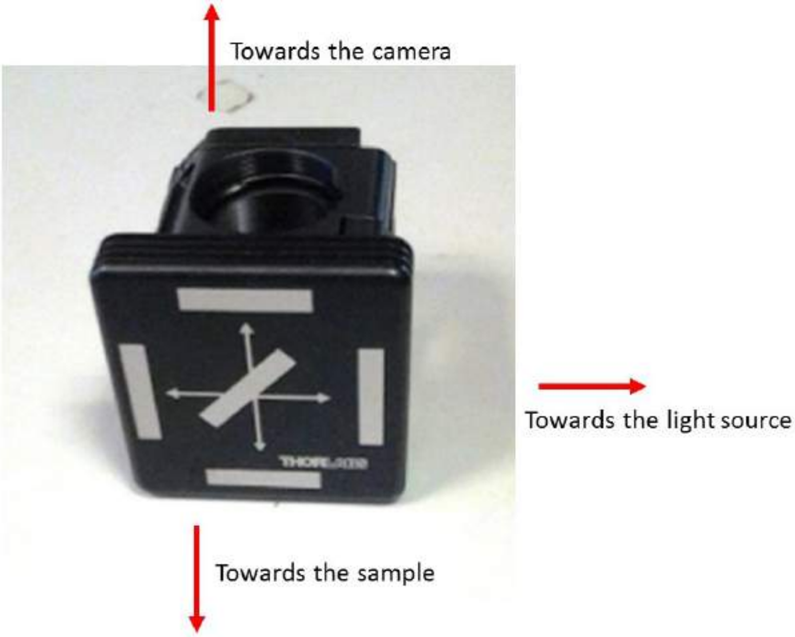

Figure 37: The top of the filter cube

2. Unscrew the 2 locking screws within cube top. Remove the insert piece in order to expose the recessed notch where the beam splitter will be placed.

3. While wearing gloves, carefully unwrap the beam splitter (BSW10R) from the lens tissue and hold the beam splitter by the edges using a pair of tweezers or with gloved hands.

4. Place the beam splitter into the recessed notch in the filter cube so that the surface with the item number engraved on it faces towards the light source and sample (see Figure 37 for orientation).

5. Make sure that the beam splitter is settled before reattaching the insert piece and tightening the locking screws. To ensure even pressure, partially tighten both screws before carefully tightening both screws fully. **Avoid overtightening as this can damage the lenses.**

6. Place the cube top back into the filter cube.
7. Label the filter cube (for example, put it in a box labeled "Brightfield cube") and store in a safe place to prevent dust from accumulating on the filters.

8.8.2 Fluorescence Filter Cube

Parts:

- Filter cube (DFM1)
- Spanner wrench (SPW801)
- Filter set (59022)
  - Beam splitter (59022bs)
  - Excitation filter (59022x)
  - Emission Filter (59022m)

Caution: The next few steps involve adding optics and should be done with care while wearing gloves.

1. Remove the top from the filter cube base.
2. Unscrew the 2 locking screws within cube top. Remove the insert piece in order to expose the recessed notch where the beam splitter will be placed.
3. While wearing gloves, carefully unwrap the beam splitter (59022bs) from the lens tissue and hold the beam splitter by the edges using a pair of tweezers or with gloved hands.
4. Identify the coated side of the beamsplitter, which has a caret pointing to it. Place the beam splitter into the recessed notch in the filter cube with the caret facing towards the light source and sample (see Figure 37 for orientation).
5. Make sure that the beam splitter is settled before reattaching the insert piece and tightening the locking screws. To ensure even pressure, partially tighten both screws before carefully tightening both screws fully. **Avoid overtightening as this can damage the lenses.**
6. Locate the circular openings where the filters will go.
7. Position the excitation filter (59022x) with the arrow pointing toward the sample and the inside of the cube/away from the light source.
8. While wearing gloves, begin to thread the filter into the opening and check to see that it is evenly threaded. Use the spanner wrench for the last couple of threads. You should be able to see a green tint when peering through the cube.
9. Position the emission filter (59022m) with the arrow pointing toward the sample and the inside of the cube/away from the camera. Repeat step 8.
10. Place the top back into the filter cube base.
11. Label the filter cube (for example, label it with the fluorophores the filters are used for or as a general "Fluorescence cube"). Store in a safe place to prevent dust from accumulating on the filters.

8.9 Pulley System

Parts:

- Metal pole (4931T123)
- Floor-mounting bracket (8809T64)
- Sideways pulley (3074T31)
- Upright pulley (3087T41)
- Padlock pulley (3099T49)
- S-shaped hook (3784T22)
- Cable Clamp (5513T14)
- 4.5 x4.5" plate (MB4A)
- 3/4" screw (x1)
- 5/8" screw (x1)
- Winch (3205T15)
- Steel Cable (3461T38)
- 6" Winch handle (0930-11Z)

1. Cut the metal pole (4931T123) to be 2 feet tall.
2. Align the 2 holes on the floor-mounting bracket (8809T64) with the 2nd and 3rd holes from the bottom of the metal pole. Pass 2" screws (x2) through the holes and thread nuts onto the end to attach the floor-mounting bracket to the metal pole.
3. Drill a hole into the top the metal pole so the S-shaped hook (3784T22) can attach onto the end of the metal pole (see Figure 38).
4. Hang the padlock pulley (3099T49) off the end of the S-shaped hook .

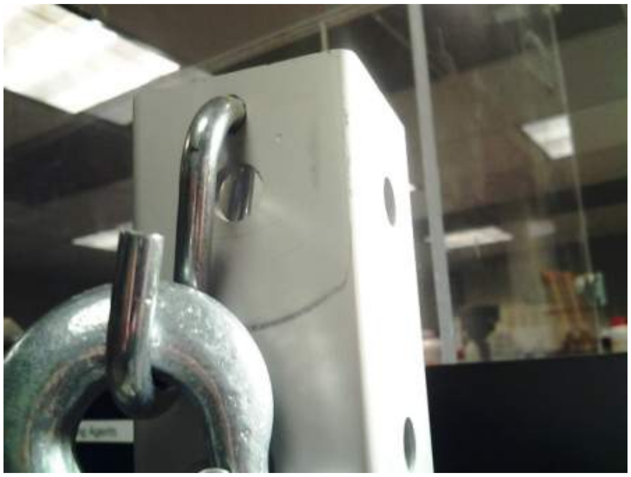

Figure 38: steps 3-4

5. Stack a small, medium, and large washer on top of each other. Slide the stack of washers onto a 3/4" screw (x1). Use the screw to attach the metal pole to the 4.5"x4.5" plate (MB4A) board (refer to Figure 39 for placement).

6. Attach the upright pulley (3087T41) to the 4.5x4.5" plate with a 5/8" screw (x1) (refer to Figure 39 for placement).

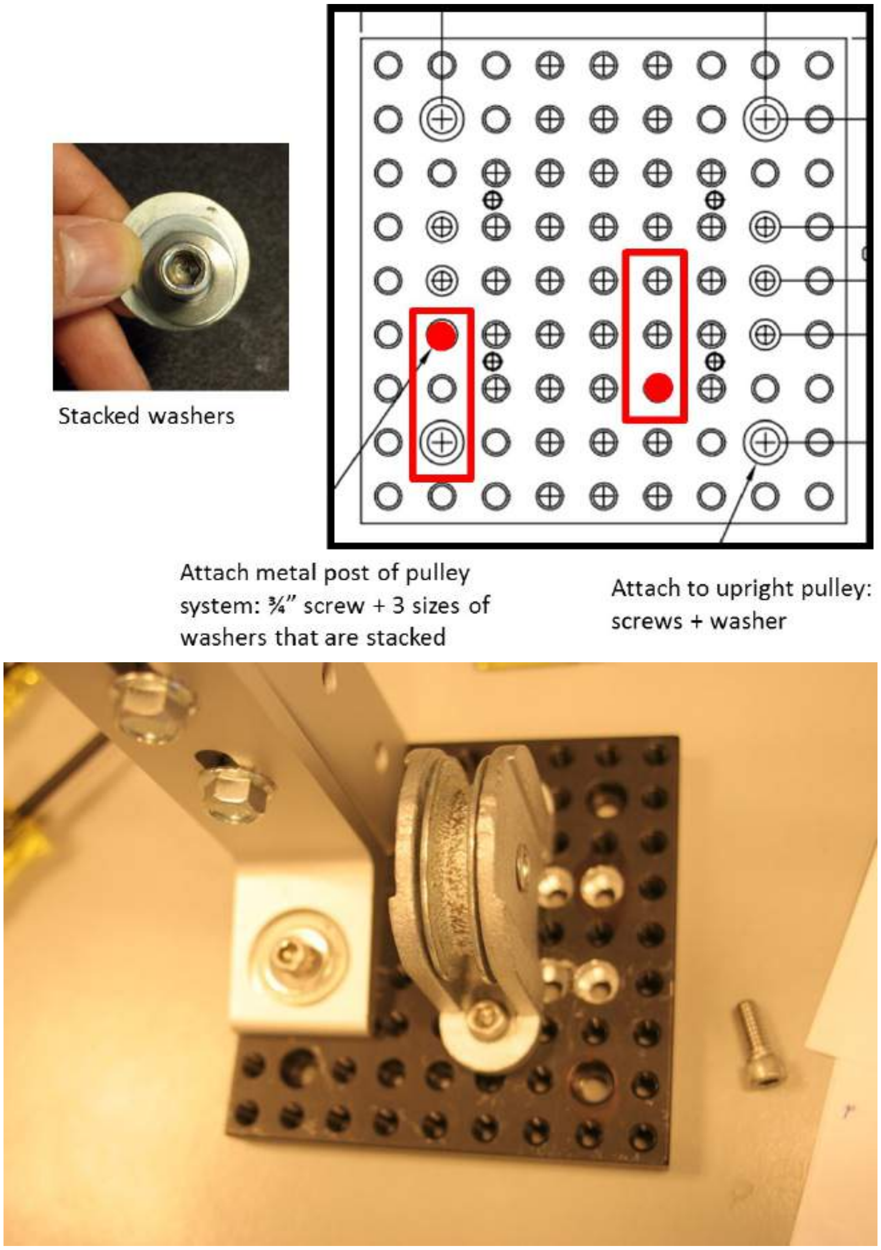

Figure 39: Diagram of bores for the placement of metal pole and upright pulley

7. Disassemble the cable clamp (5513T14). Retain the washer, plate, and nuts.

8. Thread the steel cable through the padlock pulley, the upright pulley, and the side-ways pulley.

9. Leave about a foot at the end of the cable free and use the cable clamp to keep the steel cable from slipping through the padlock pulley.

10. Remove the 8" handle of the winch and attach the 6" handle. Use the nut that came with the winch to attach the 6" handle.

11. Pass the cable through the winch and follow the instructions provided with the winch to tie the end of the cable to the winch spool.

12. Wind up the cable for the first time following the instructions provided with the winch. Keep the tension in the cable so it is wound tightly onto the spool.

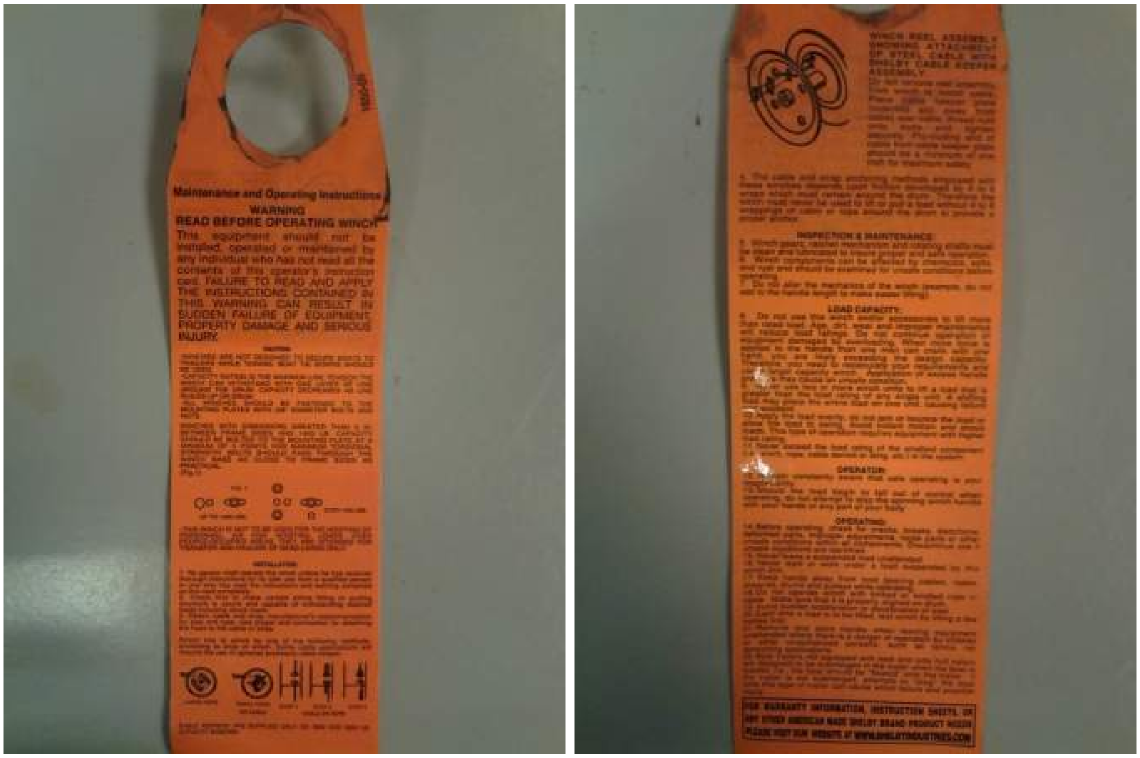

Figure 40: Instructions for winch

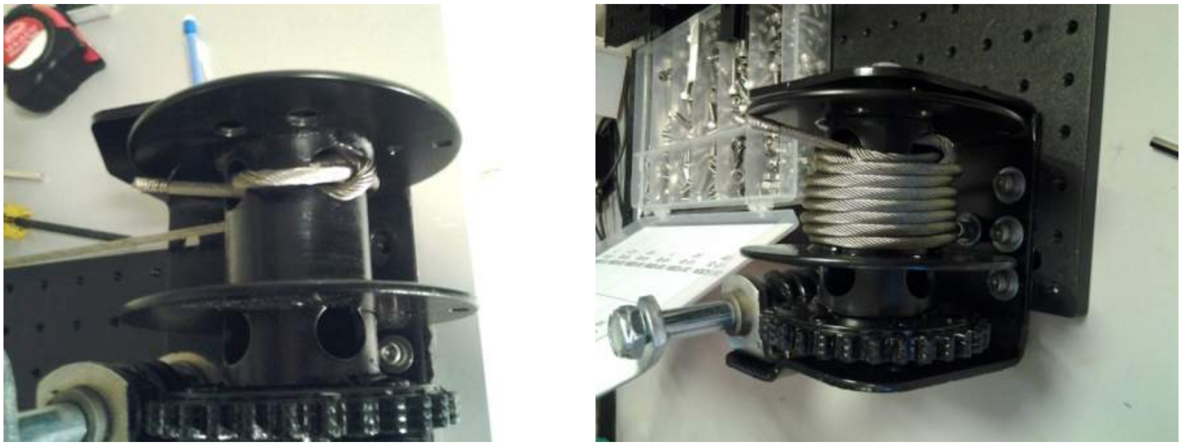

Figure 41: Knot to begin winding steel cable on winch and the wound up steel cable, and overall pulley system

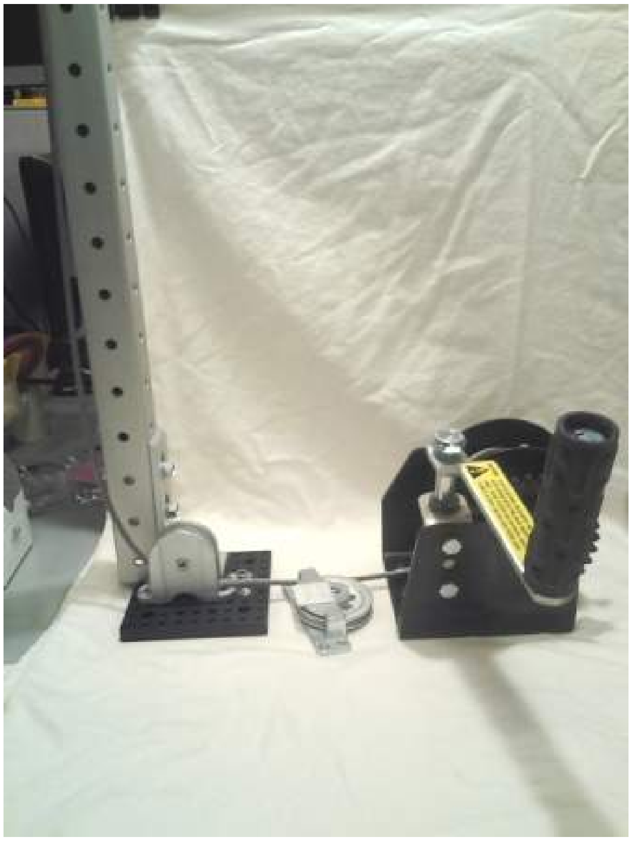

Figure 42: Overall pulley system

8.10 Ellipsometer

8.10.1 Analyzer

Parts:

- Cage rotation mount for optics (CRM1)

- Polarizer (47-215 [Edmund's Optics]))

1. Thread the polarizer into the cage rotation mount.
2. Label it as the "analyzer" and store in a case/box to avoid dust from accumulating. This will be needed for imaging ellipsometry.

8.10.2 Ellipsometer Mount Stage

Parts:

- 4x6 plate (MB4)
- 90 degree bracket (AP90)
- Rotating mount (SL20)
- Small 90 degree bracket(AB90A)
- 3/4" screws (x8)

1. Locate the side of the 90 degree bracket (AP90) that contains the Thorlabs logo and two clearance slots. Place this side flush against the 4x6 plate (MB4). Attach the 90 degree bracket to the 4x6 plate using 3/4" screws (x2) passed through clearance slots and threaded into the plate (refer to Figure 43 and 45 for placement and orientation).

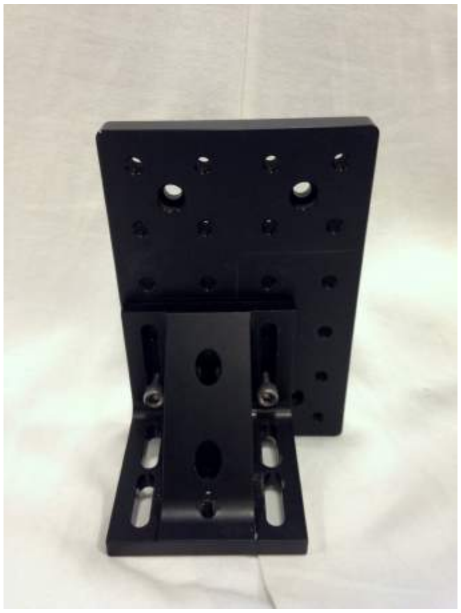

Figure 43: Step 1

2. Use 3/4" screws (x4) to attach the base of the rotating mount (SL20) to the side of the 4x6 plate that does not contain the 90 degree bracket. Refer to Figure 44 and 45 for placement.

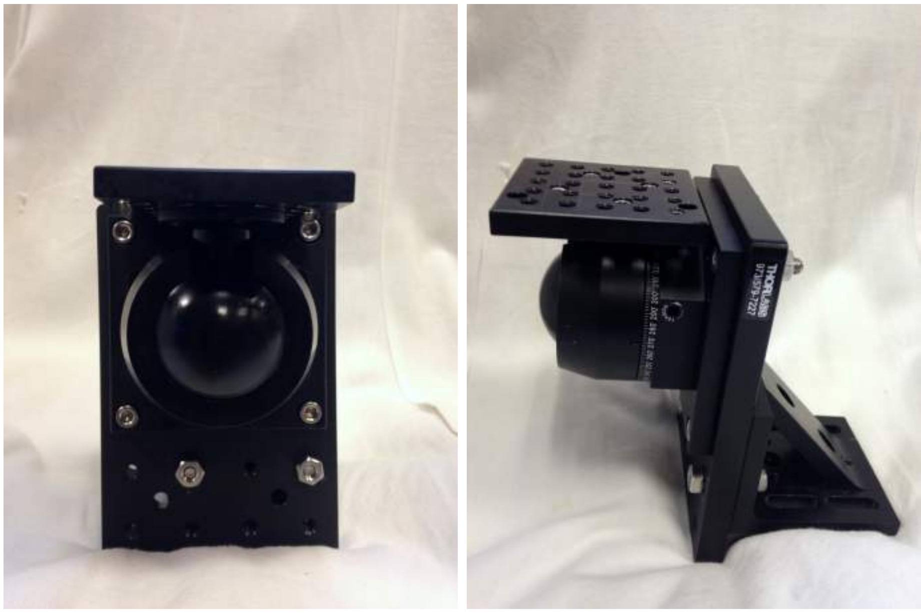

Figure 44: Step 2

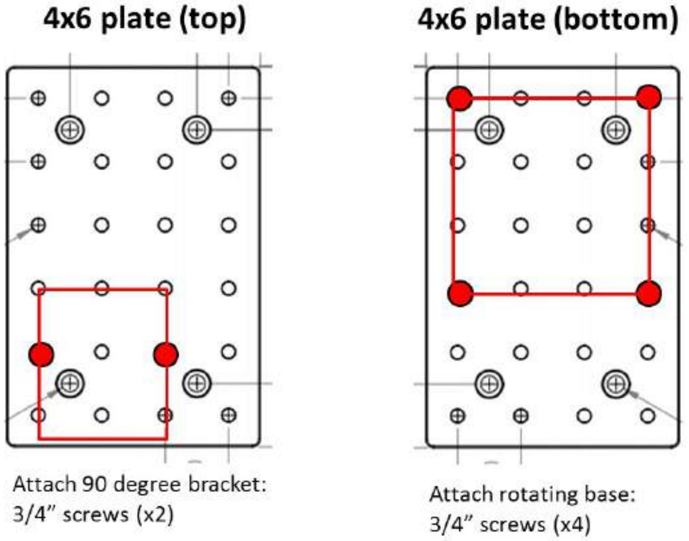

Figure 45: Diagrams for the placement of the 90 degree bracket and rotating base on the 4x6 plate

3. Place the side of the small 90 degree bracket (AB90A) with the Thorlabs logo flush against the platform on the rotating mount. Attach the small bracket to the platform using 3/4” screws (x2) threaded through the clearance slots of the small bracket and into the bores on the platform. Refer to Figure 46.

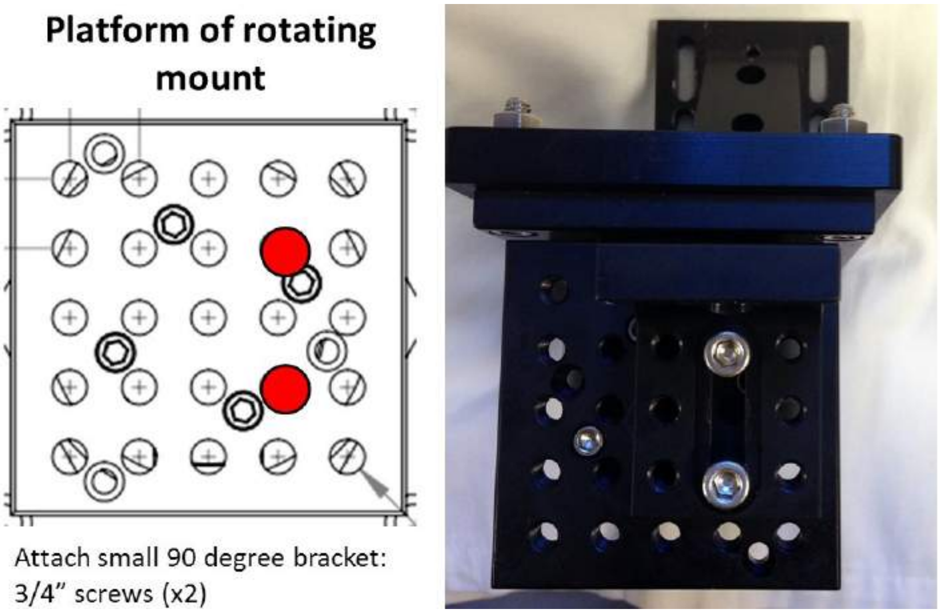

Figure 46: Step 3

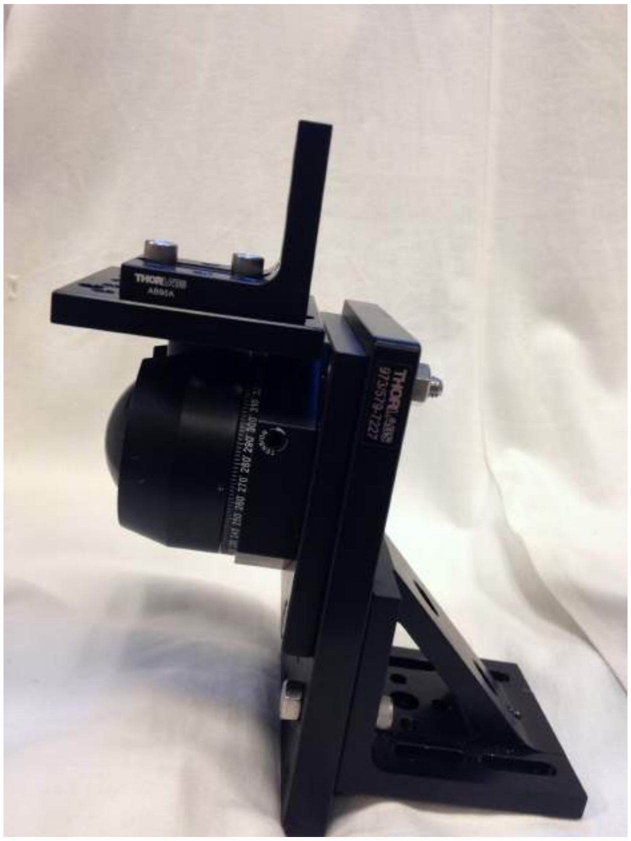

Figure 47: Finished ellipsometry mount stage

8.10.3 Laser Cage

Parts:

- 532nm laser (CPS532)
- Laser holder (AD11F)
- Laser plate (CP6S)
- Cage rotation mount for optics (CRM1) (x2)
- Polarizer (47-215 [Edmund's Optics])
- Cage bracket (CP02B)
- Small 90 degree bracket (AB90A)
- 4" posts (ER4)(x4)
- Compensator/wave plate (WPMQ05M-532)

Refer to the following image while building the laser cage.

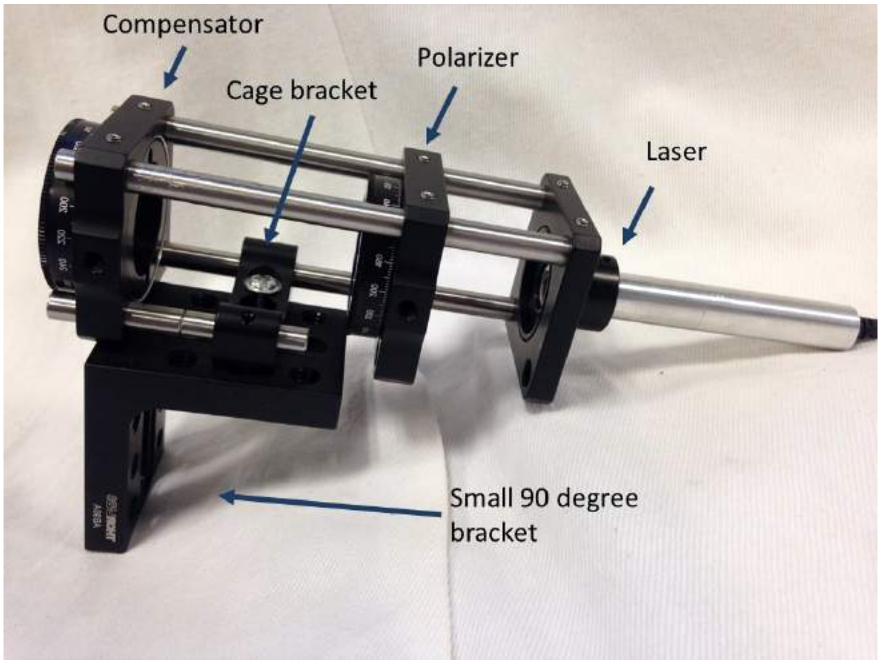

Figure 48: Fully assembled laser cage

1. Thread the laser holder (AD11F) into the laser plate (CP6S).
2. Slide the laser (CPS532) into the laser holder. Tighten the setscrew on the side of the holder to keep the laser in place.
3. Slide three 4" posts into the laser plate (see Figure 48). Level the posts and tighten the setscrews to keep the posts in place.

4. Thread a polarizer (Edmund's optics 47-215) into a cage rotation mount (CRM1). Slide the cage rotation mount onto the posts with the circular portion pointing away from the laser plate. Adjust it until it is 1" from the laser plate before tightening the setscrews to hold it in place.

5. Slide the cage bracket (CP02B) onto only ONE post (see Figure 48). Adjust it until it is about 1" from the polarizer in step 4.

6. Slide a 2" post into the remaining hole in the cage bracket.

7. Thread the compensator (WPMQ05M-532) into cage rotation mount (CRM1). Slide the cage rotation mount onto the end of the posts. Tighten the setscrews on the side to hold it in place.

8. Align the cage bracket (CP02B0) on top of a small 90 degree bracket (AB90A) so that the face of the AB90A spans the distance from the compensator to the polarizer.

9. Thread a 5/8" screw through the leftmost hole in the cage bracket and the corresponding hole on the AB90A. Refer to Figure 49.

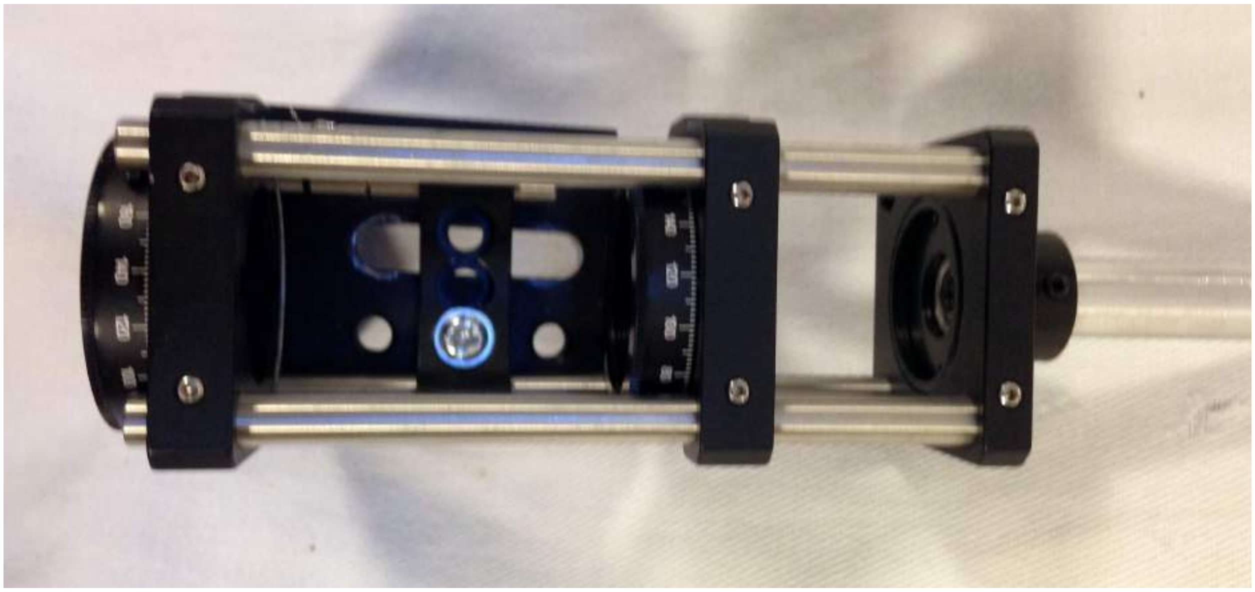

Figure 49: Step 9

8.10.4 Ellipsometry Arm

Parts:

- Ellipsometry mount stage (assembled in section *8.10.2*)
- Laser cage (assembled in section *8.10.3*)
- 1" Screws (x2)
- Nuts (x2)

1. Use 1” screws (x2) to fasten the small 90 degree bracket in the laser cage to the other small 90 degree bracket in the ellipsometer mount stage. Pass the screws through the clearance slots of both small brackets and thread nuts onto the end of each screw to hold them in place. (The heights of the brackets relative to each other will need to be adjusted according to your set up.)

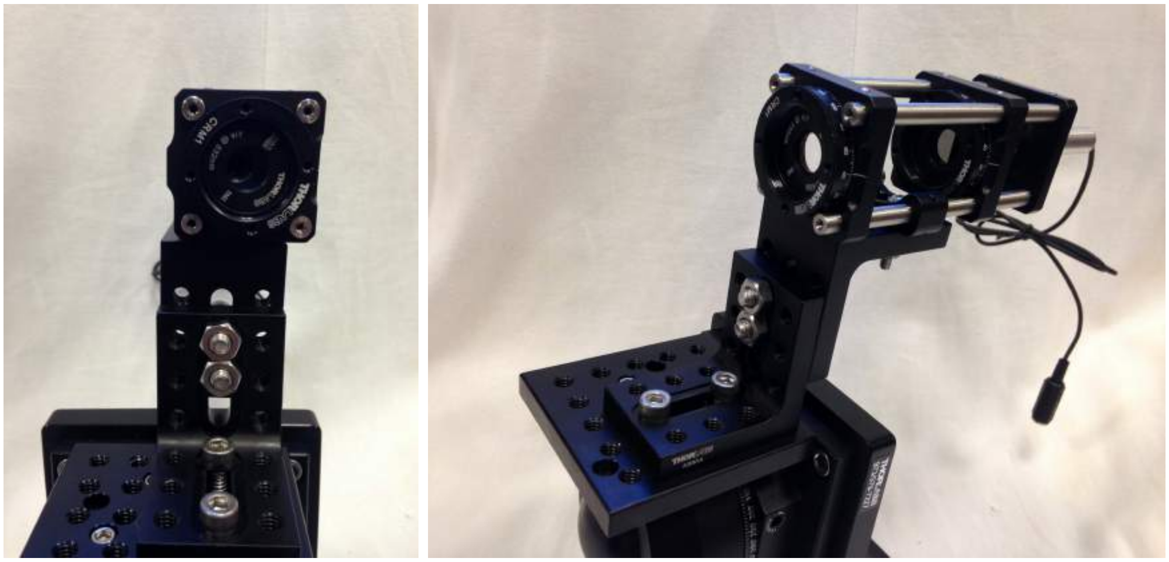

Figure 50: Step 1 to connect the laser cage to the ellipsometry mount stage

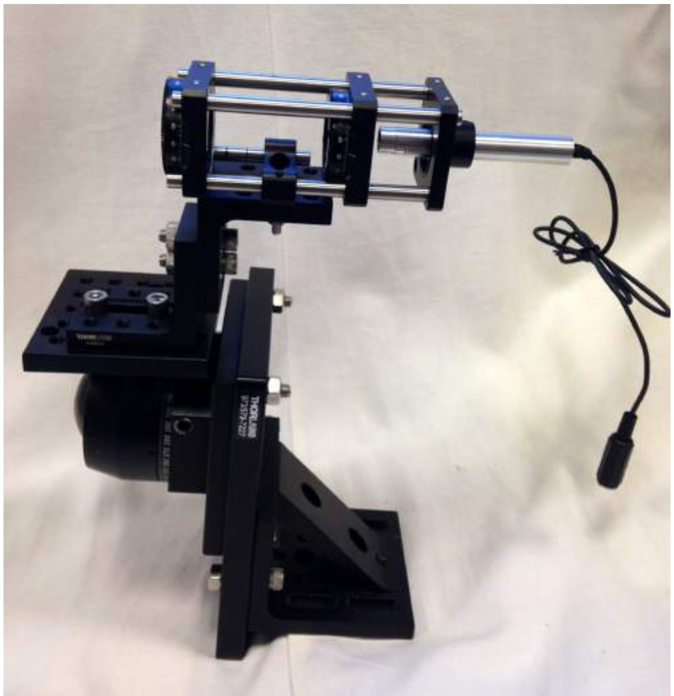

Figure 51: Fully assembled ellipsometer after the two groups have been combined. This is the position when the ellipsometer is not being used.

9 Installation Preparation

Note: The procedure for preparing the installation space for the SwingScope may vary depending on the available workspace. Some considerations include having adequate room for a table that is at least large enough to have a 24x12" board bolted down to it and enough room to enable the winch handle and SwingScope to rotate freely (at least 3 feet around the table). The space should also have access to a power outlet and room for a computer table/cart. Outlined below is the process for the installation of the SwingScope in our particular workspace, but can be adapted to fit other spaces.

9.1 Microscope Table for Brightfield and Fluorescence

Parts:

- Main board, 24x12" (MB1224)
- 8x8" plate (MB8)
- Rubber matting (1310N55)

1. Install table for microscope in desired workspace. Table size will vary depending on available work space, but there should be at least 2.5 feet of space from the edge of the table for the SwingScope to rotate freely.
2. Center the main board (MB1224) on the table and adjust the board so it is flush with the table's edge.
3. Place a rubber mat between the table surface and the main 24x12" board onto which the SwingScope will be mounted in order to dampen vibrations.
4. Drill holes into the table and the rubber matting to enable the main board to be bolted to the table.
5. Clean the table surface and rubber matting of debris.
6. Bolt the board down the table using 2 1/2" screws, washers, and nuts (screw length required will vary with table thickness). Trim rubber matting if needed.
7. Attach the 8x8" plate (MB8) that will serve as the base for pulley system and rotation platform to the main board with 5/8" screws through the back two countersinks of the plate (refer to Figure 52 for placement).

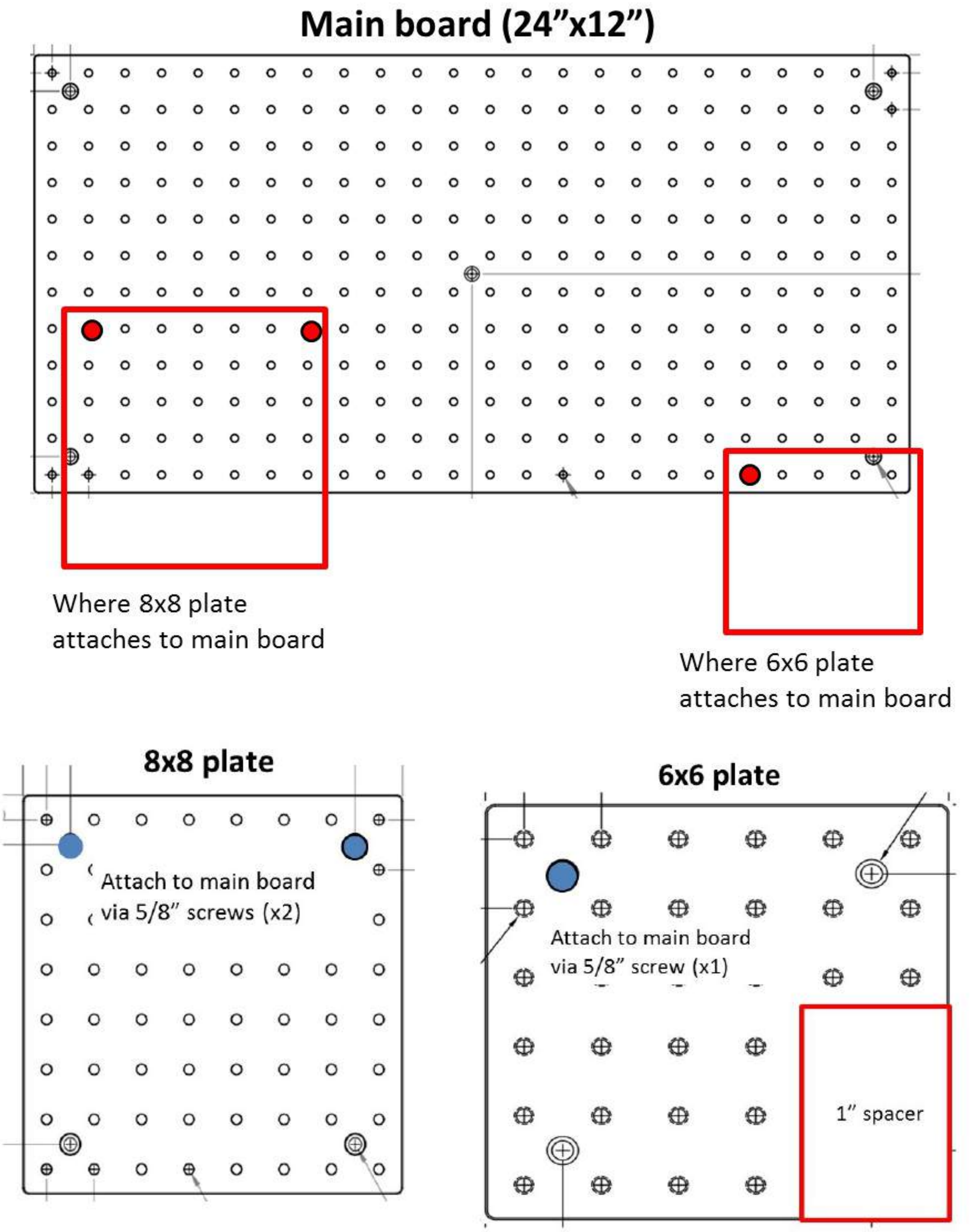

Figure 52: Diagrams for the set up of the microscope table for fluorescence and ellipsometer

9.2 Microscope Table for Ellipsometry

The setup for ellipsometry capabilities may be added to the basic setup for brightfield and fluorescence.

Parts:

- 6x6" plate (MB6)
- 1" spacer (BA2S7)
- 5/8" screw (x1)
- 1.5" screws (x2)

1. Place a 1" spacer (BA2S7) on the bottom corner of the 6x6 plate (MB6) so that the edges of the spacer and the plate are aligned. Attach the spacer to the plate using 1.5" screws (x2) threaded through the counterbores on the spacer (located on the side of the spacer without an labels/logos) and the holes on the plate. Refer to Figure 53 for placement.
2. Align the top row of pin holes on the 6x6 board (MB6) with bottom row of holes on the main board. Attach the 6x6 plate to the main board using a 5/8" screw (refer to Figure 52 for placement).

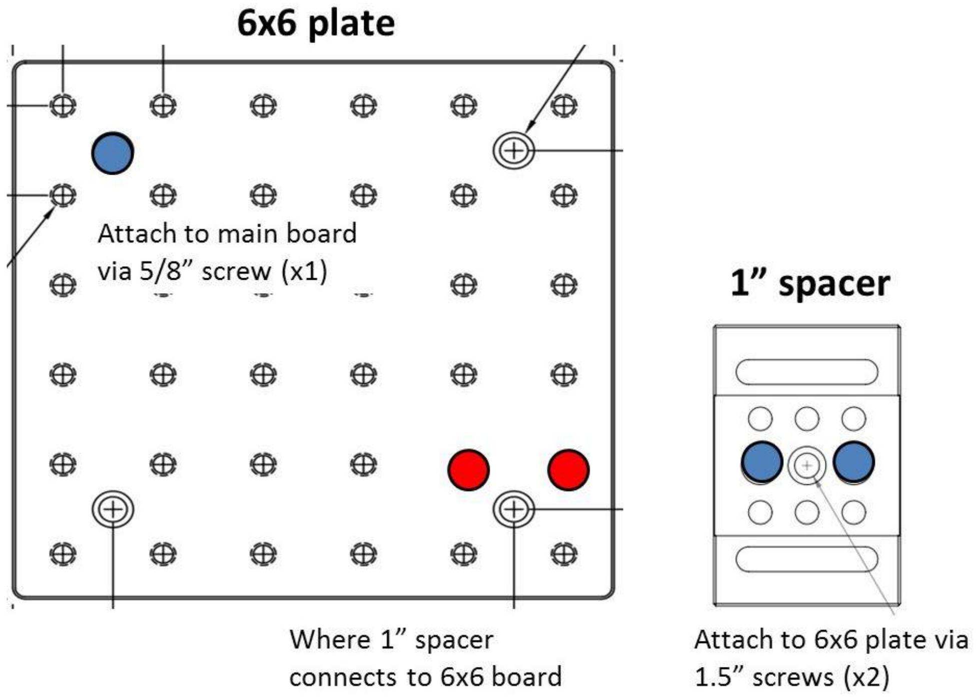

Figure 53

9.3 Computer Table/Cart

1. Assemble desired computer and computer cart. Install in workspace.

10 SwingScope Installation Procedures

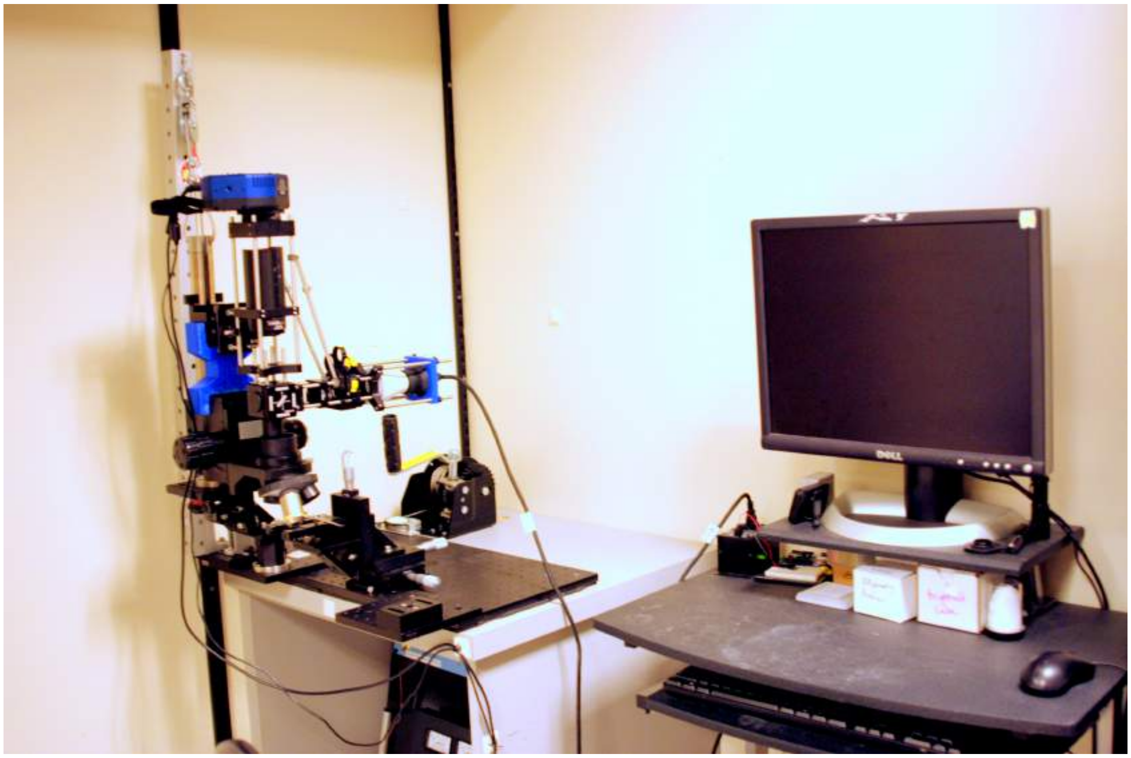

Figure 54: Fully installed SwingScope

This section outlines the procedure to put all of the groups assembled in the section *8 Assembly Procedure* and install the SwingScope in a workspace.

10.1 Diagrams for Installation

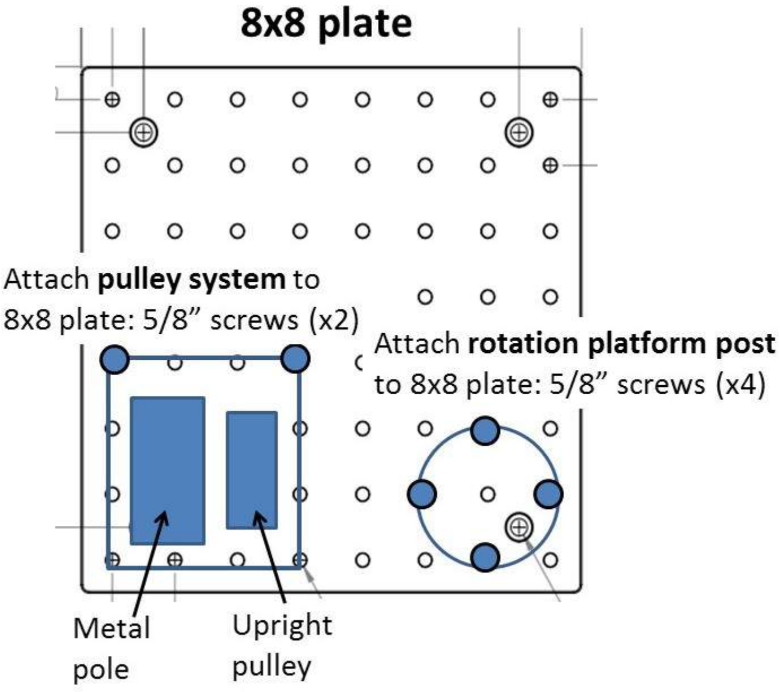

Figure 55: Where to attach the pulley system and rotation platform post to the 8x8 plate

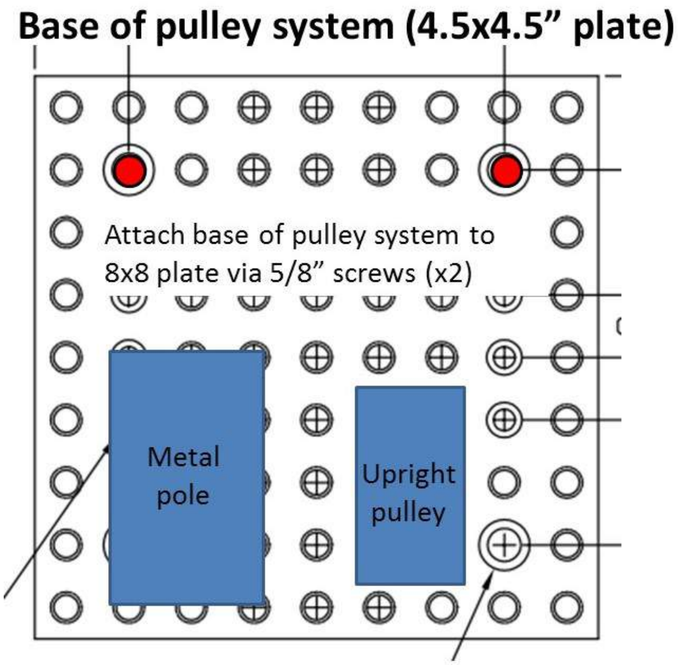

Figure 56: How to attach the base of the pulley system to the 8x8 plate

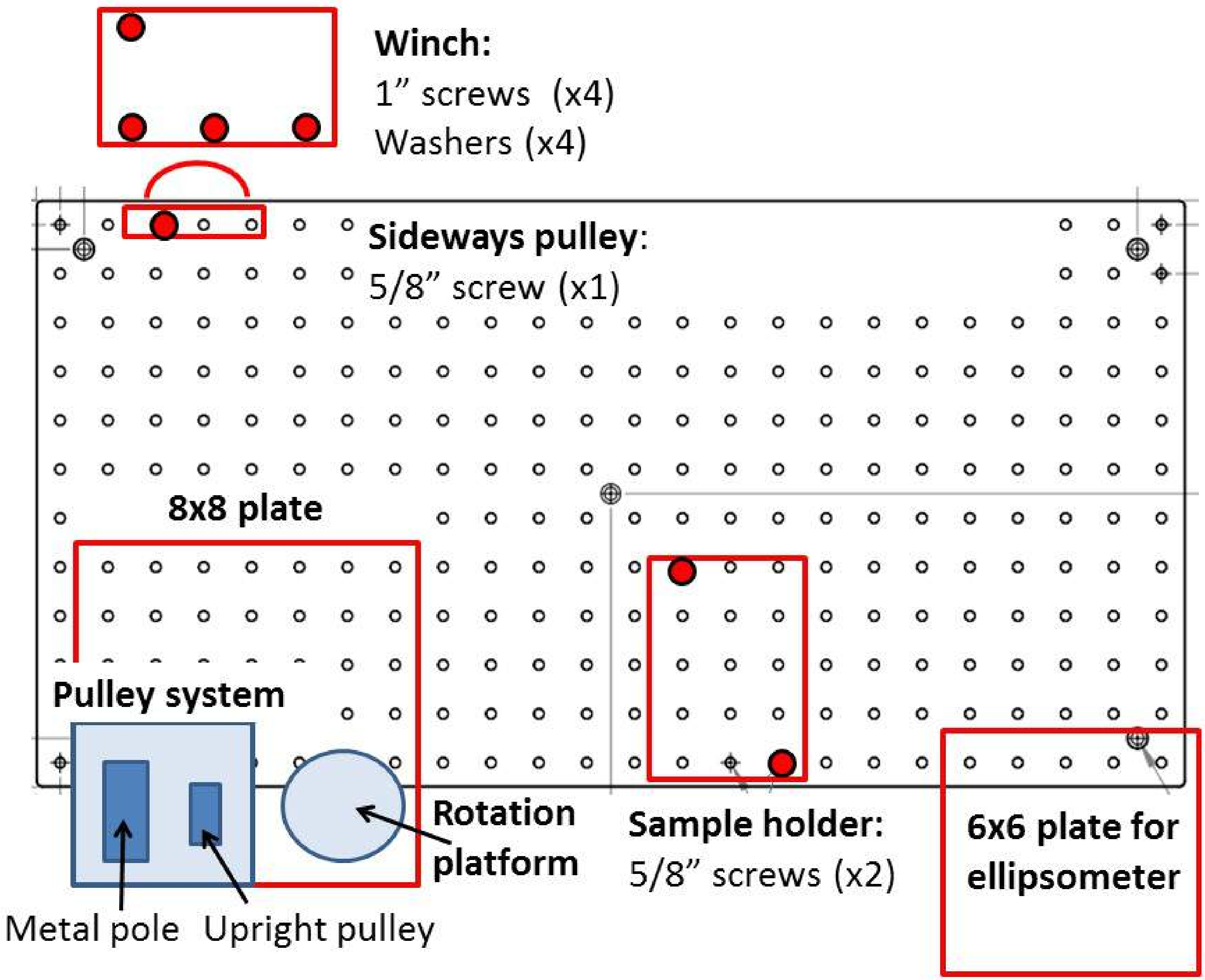

Figure 57: Overall setup of the components directly attached to the main board

10.2 Pulley System

Parts:

- Pulley System (group assembled in section 8.9)
- 5/8” screws (x7)

1. Place the winch in the top-left corner of the table. Use a power drill to create 4 holes in the table that correspond with the slots at the base of the winch. Attach the winch to the table using 1” screws and washers (refer to Figure 57).
2. Place the sideways pulley in the top-left corner of the main board and orient it so the circular side of the pulley is pointed towards the winch (refer to Figure 57 for placement). Attach the sideways pulley to the main board with a 5/8” screw (x1).
3. Attach the 4.5x4.5” plate that forms the base of the pulley system (group assembled in section 8.9) to the 8x8” plate using 5/8” screws (x2) threaded through the counterbores of the plate (refer to Figure 55 and 56 for placement).

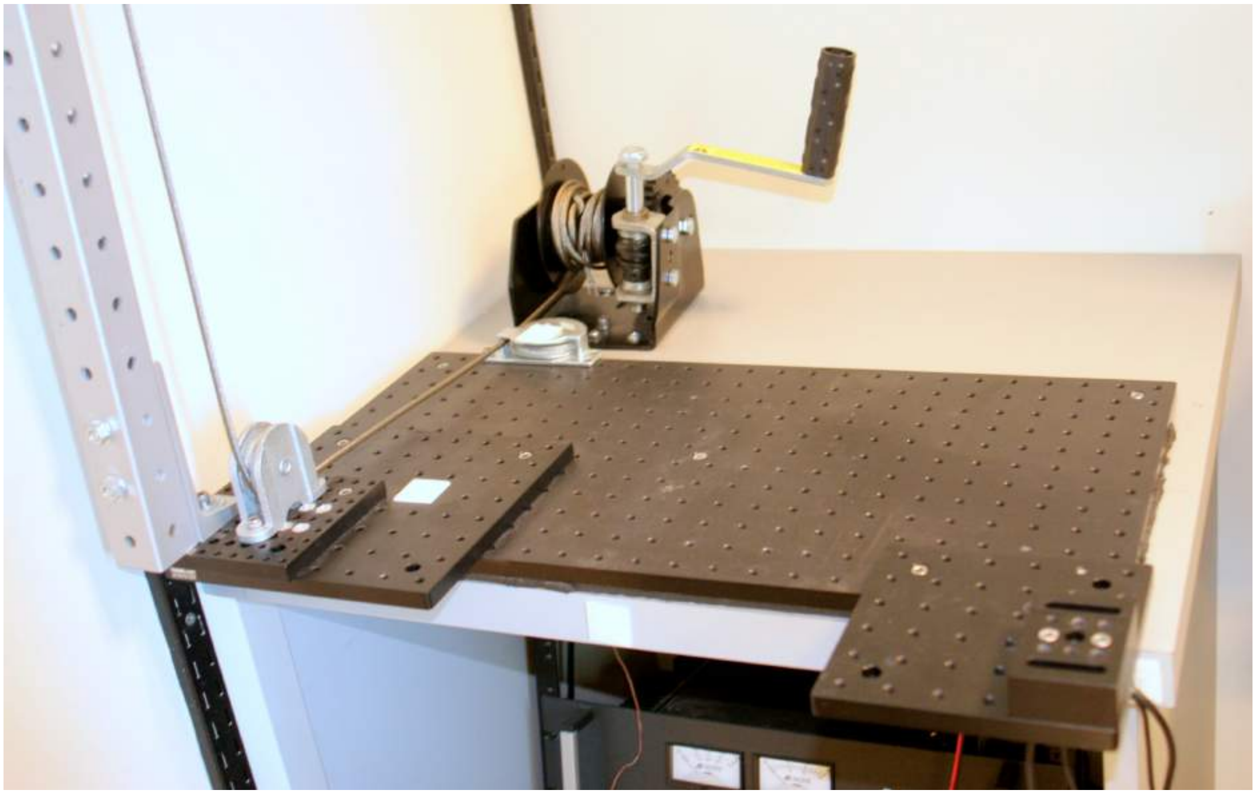

Figure 58: Overall setup after installation of pulley system

10.3 Rotation Platform

Parts:

- Rotation platform post (assembled in section 8.2)
- Rotation platform (assembled in section 8.5)
- Vertical platform (C1519)
- 5/8" screws (x4)
- 3/4" screws (x4)

1. Fasten the base of rotation platform post (group assembled in section 8.2) to the 8x8" plate with 5/8" screws (x4) (refer to Figure 55).Tighten the screws using a locking key.
2. Slide the vertical platform (C1519) all the way down onto the post and tighten the locking handle on the side.
3. Locate the 2" clearance slots at the base of the 90 degree bracket that is part of the rotation platform (group assembled in section 8.5). Use washers (x4) and 3/4" screws (x4) threaded through the clearance slots to attach the rotation platform to the vertical platform (refer to Figure 59 below). Adjust the 90 degree bracket so it is as far forward as possible before tightening the screws.

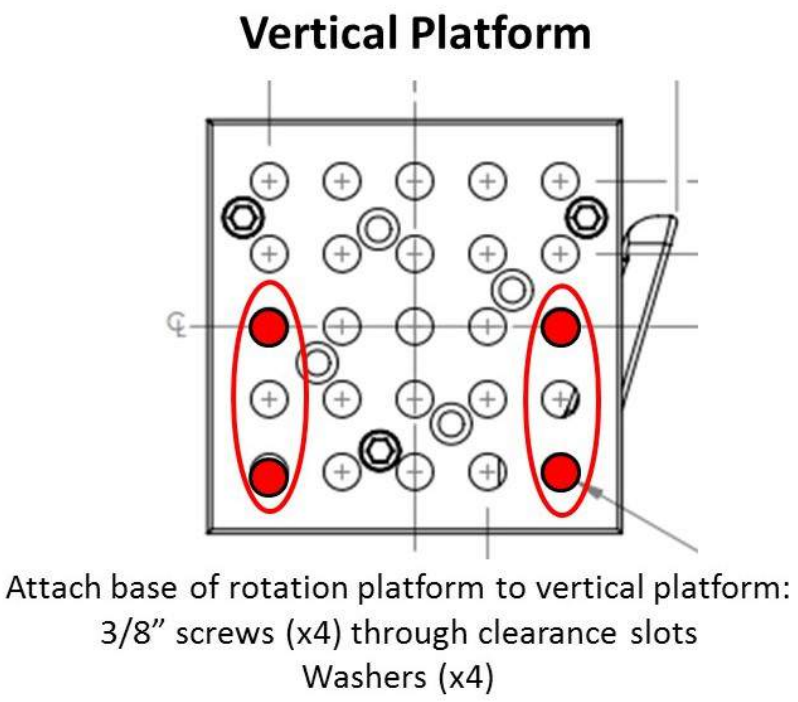

Figure 59: Placement of the 90 degree bracket that forms the base of the rotation platform relative to the vertical platform

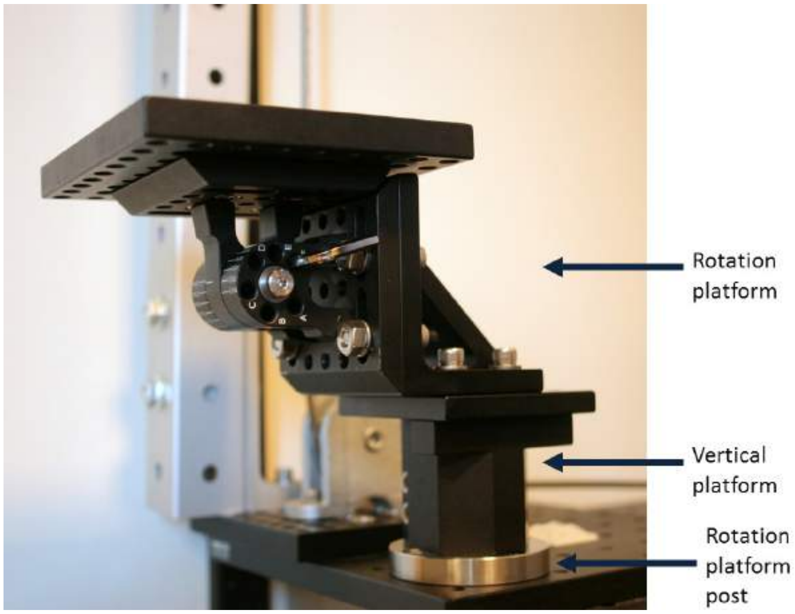

Figure 60

10.4 Mounting Post

Parts:

- Mounting post (assembled in section 8.1)
- 5/8" screws (x4)

1. Center the base of the mounting post on top of the rotation platform installed in the last section. Use 5/8" screws (x4) to connect the mounting post to the rotation platform (refer to Figure 61 for placement)

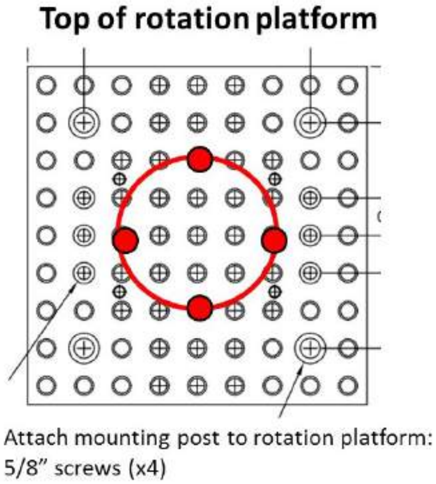

Figure 61

10.5 Focus Block

Parts:

- Focus block (MGZ30)
- Objective adapters (M25A1) (x6)
- C-clamp (C1498)

1. Remove the adapter rings that come with the the rotating platform of the focus block. Replace them with M25A1 adapters.
2. Place a c-clamp (C1498) around the mounting post 13 mm from the base of the mounting post. Lock the c-clamp in place using the locking screw on the side.
3. Slide the focus block onto the mounting post with the lock handle on the right side of the post, facing the back of main board/winch. Adjust the focus block so that it rests on the c-clamp.
4. Keep the focus block oriented parallel to the main board. Lock the focus block in place using the lock handle.

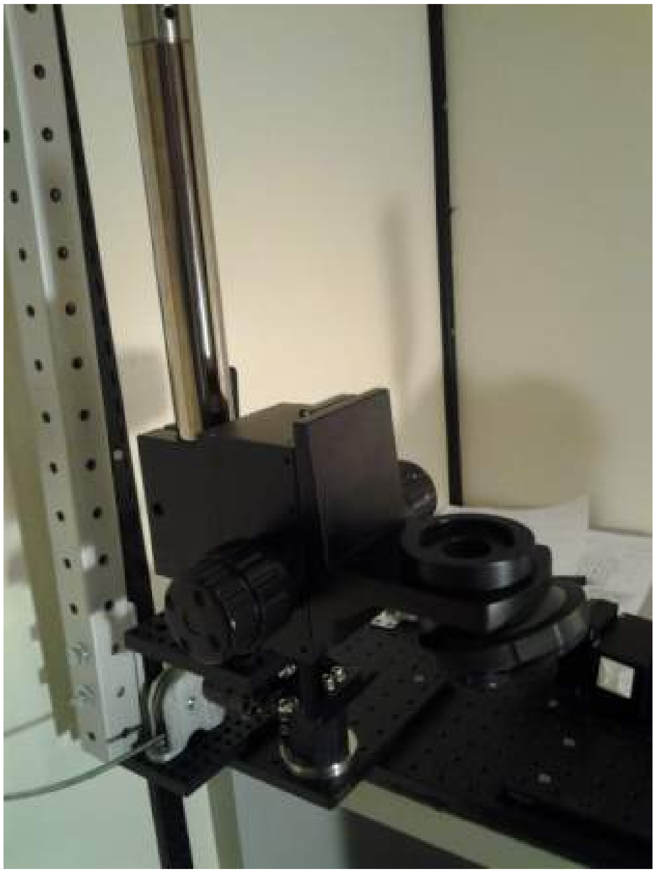

Figure 62: Installed focus block

10.6 Mount-cage Extension

Parts:

- H-piece (3D printed)
- Mount-cage extension (assembled in section 8.3)
- C-clamp (C1498)

1. Attach the bottom of the H-piece (which contains three sides) to the top of the focus block. Make sure the fit is snug.
2. Slide the mount-cage extension (group assembled in section 8.3) onto the mounting post. Snap the 2” spacer of the mount-cage extension into place on the H-piece.
3. Place a c-clamp (C1498) around the mounting post so that it is flush with the top of mount-cage extension. Lock it in place using the screw on the side.

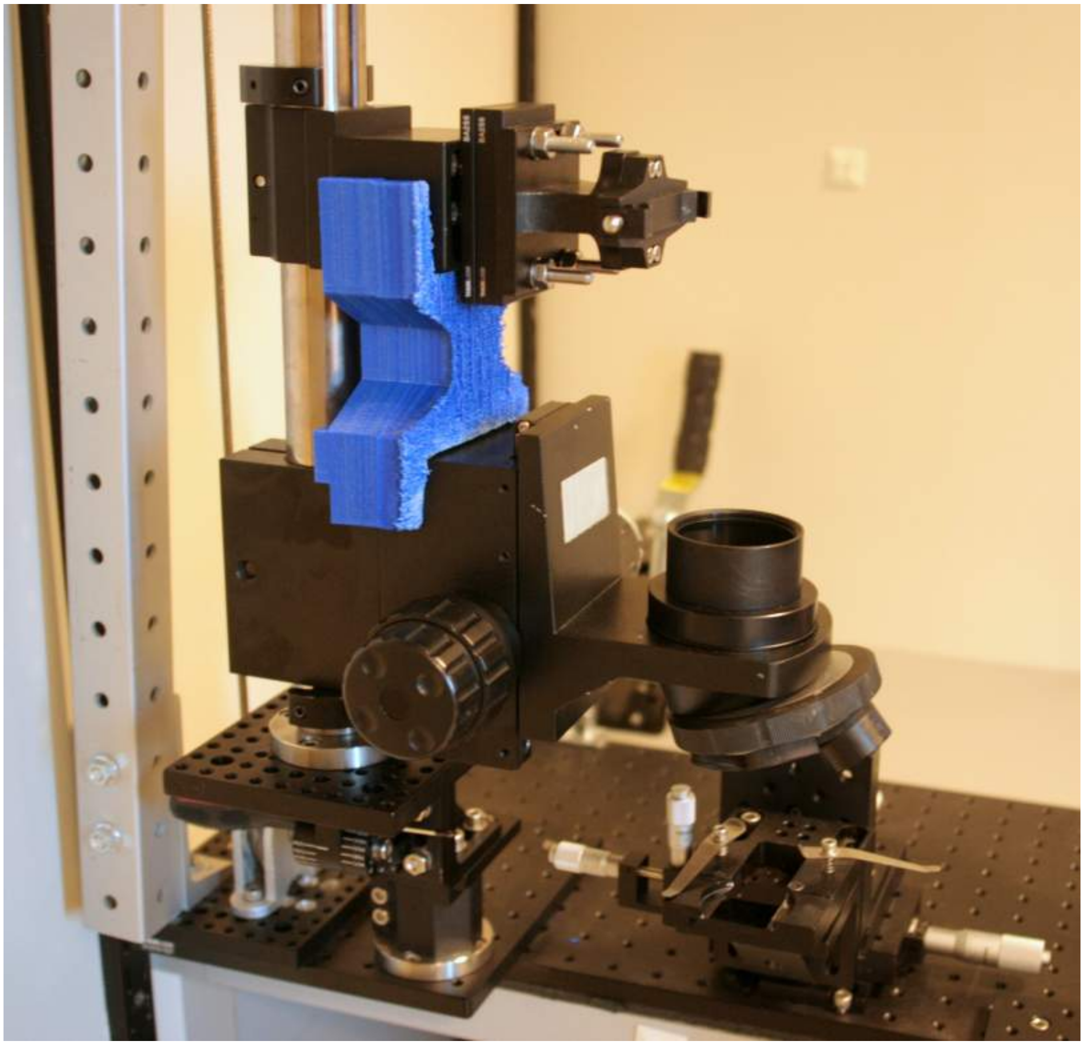

Figure 63: Installation of the H-piece and mount-cage extension

10.7 Secure Mounting Post

1. Feed the steel cable through the hole at the top of the mounting post.
2. Form a tear-drop shape and loop the end of the cable through the clamp.
3. Tape the end of the steel cable to keep it from fraying.
4. Use the plate, washers (x2), and nuts (x2) that came with the cable clamp to secure the clamp on top of the cable. Use a wrench to tighten the nuts.
5. Wind up the remaining cable until the cable by the metal pole is relatively taunt, but be careful to not over tighten as this can cause the cable to snap.

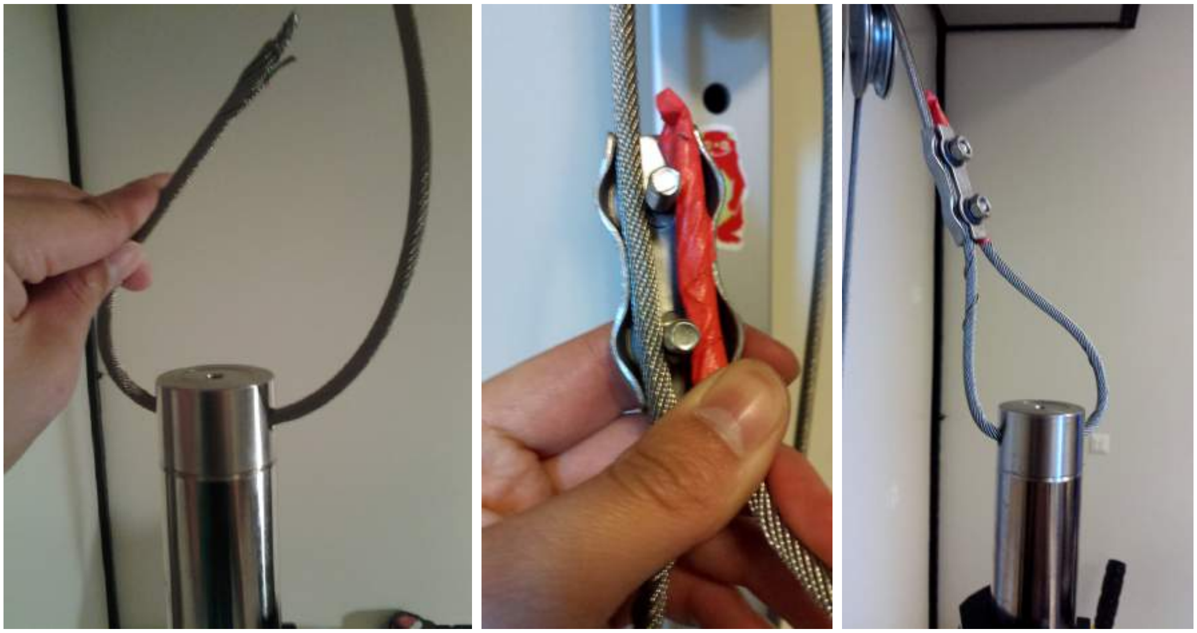

Figure 64: Loop the steel cable through the top of the mounting post and use the cable clamp to secure the mounting post (Steps 1-4)

10.8 Sample Stage

Parts:

- 5/8" screws (x2)
- Sample Stage (group assembled in section 8.4)

1. Use the knobs on the sample stage to position each axes (x,y, and z) at the mid point of their total travel. This should be at the "5" tick mark.
2. Use 5/8" screws (x2) threaded through the back-left and front-right corners of the base of the sample holder to connect it to the main breadboard (see Figure 57 for placement). Make sure the sample stage is parallel to the main board.

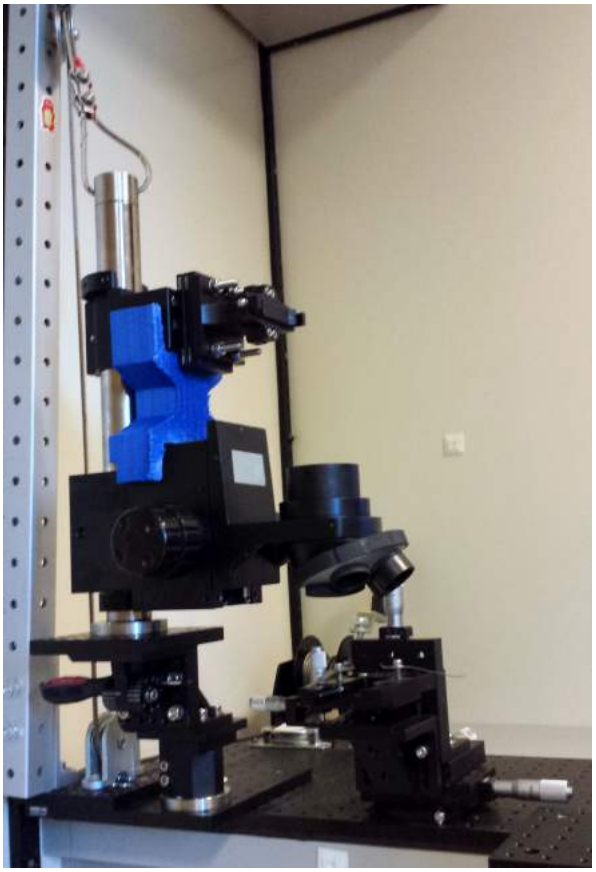

Figure 65: Installation of sample holder

10.9 Upright Cage System

Parts:

- Upright Cage System (group assembled in section 8.7)

1. Bring the focus block platform all the way up by twisting the focus knobs on the side.
2. Orient the upright cage system (group assembled in section 8.7) so that the swivel couplers are pointing towards the back of the main board and towards the winch.
3. Settle the bottom of the filter cube on the focus block platform.
4. Place the metal rods of the upright cage system into the cage grip of the mount-cage extension.
5. Tighten the clamping screw on the side to keep the cage system in place.
6. OPTIONAL: Cut a piece of PCP tube that is 2.25” in diameter and 3.75” long. Place above the tube lens to help block light leakage.

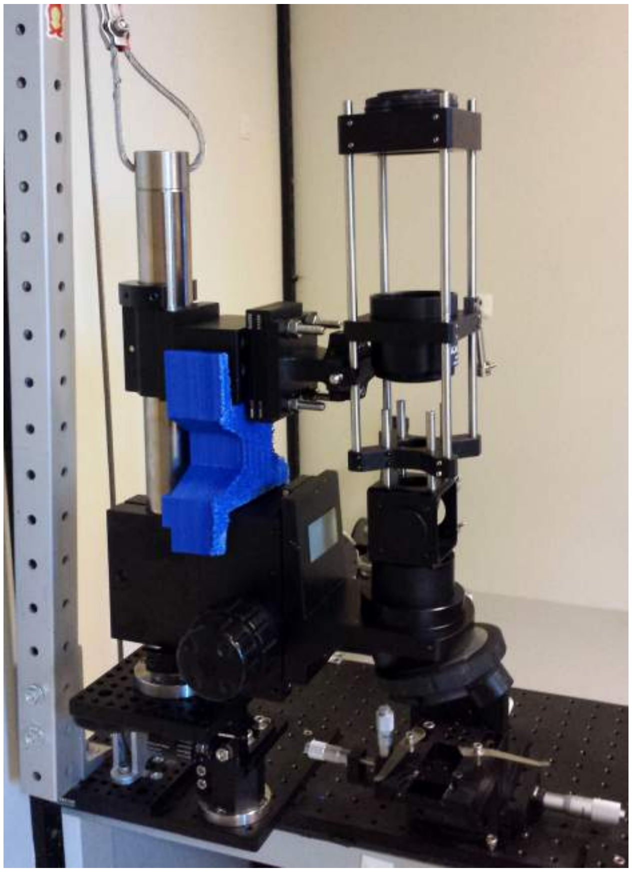

Figure 66: Installation of upright cage system

10.10 Kohler Illumination Arm

Parts:

- Kohler illumination arm (group assembled in section 8.6)

1. Align the metal posts of the Kohler illumination arm (group assembled in section 8.6) with the 4 pockets on the side of the filter cube of the upright cage system.
2. Tighten the setscrews on the sides of the pockets of the filter cube to keep the system in place.
3. Locate the swivel couplers on the Kohler Illumination arm. Note: the swivel couplers have three screws: 2 locking screws on the bottom and a rotation locking screw on the top.
4. Loosen the locking screws holding the swivel couplers on the Kohler illumination arm. Adjust them so that they are flush with the filter wheel. Loosely tighten the bottom locking screws to hold them in place.
5. Loosen the locking screws holding the the swivel couplers on the upright cage system. Adjust their vertical position on the upright system until the posts attached to the swivel couplers on the Kohler illumination arm can fit into the pockets attached to the swivel couplers on the upright cage system.

6. Adjust the angle and distance of the swivel couplers on both the Kohler illumination arm and upright cage system until they are as taunt/stable as possible. Try rotating the swivel couplers outwards. Make sure the posts attached to the swivel couplers can still fit into the pockets.

7. Tighten all the screws on the swivel couplers to hold them in place.

8. Tighten the setscrews on the pockets to hold the posts in place.

9. Make sure the cage system is securely held and not sagging.

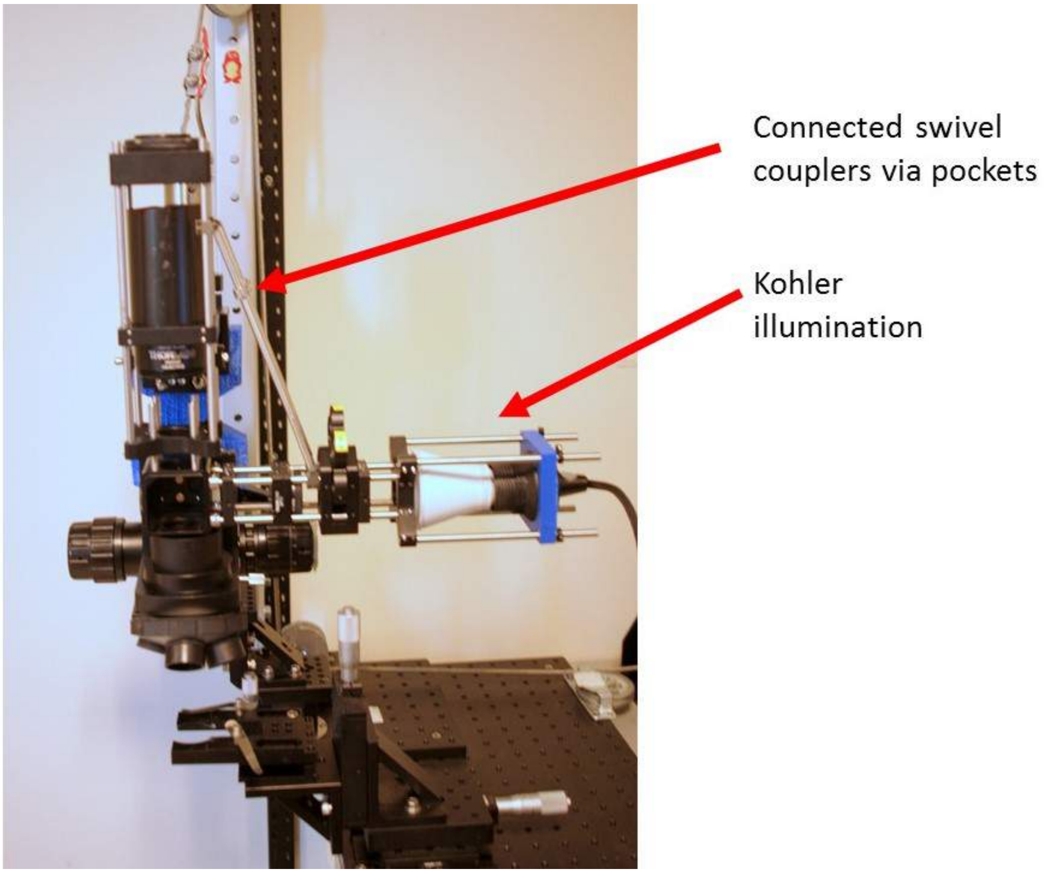

Figure 67: Installation of Kohler Illumination Arm

10.11 Objectives

Parts:

- SM1A28 Adapter Ring (x2)
- 0.5” lens tube (SM3L05) (x2)
- RMSA7 Adapter Ring (x2)
- 4x objective (optional)
- 10x objective (N10X-PF)

- 40x objective (Olympus LUCPlanFLN)

Note: The objectives listed above were the ones used in our microscope, but other objectives can be used depending on the application.

1. Adjust the x and y rails of the sample stage so it is as far back as possible.
2. **Read section 13.1 Rotating the SwingScope before proceeding!**.
3. Invert the microscope by removing the linchpin from the rotation platform and rotating the winch.
4. While wearing gloves, rotate the objective platform located at the bottom of the focus block until the desired opening is as far from the focus block and the sample holder as possible.
5. Thread desired objective into the objective adapter rings. One hand should be on the objective at all times and caution should be exercised while threading.
6. Repeat for each opening/objective (see images below for the placement).
   (a) For 4x and 40x objectives: thread a SM1A28 Adapter to a 0.5" lens tube (SM3L05). Thread a RMSA7 adapter to the other side of the tube lens. The SM1A28 adapter connects to the objective platform on focus block while the RMSA7 threads to the objective.
   (b) For Nikon 10x objective: Thread into the M25A1 adapter ring that was previously added to the focus block.
7. After the objectives are installed, carefully bring the microscope back to it's upright position.

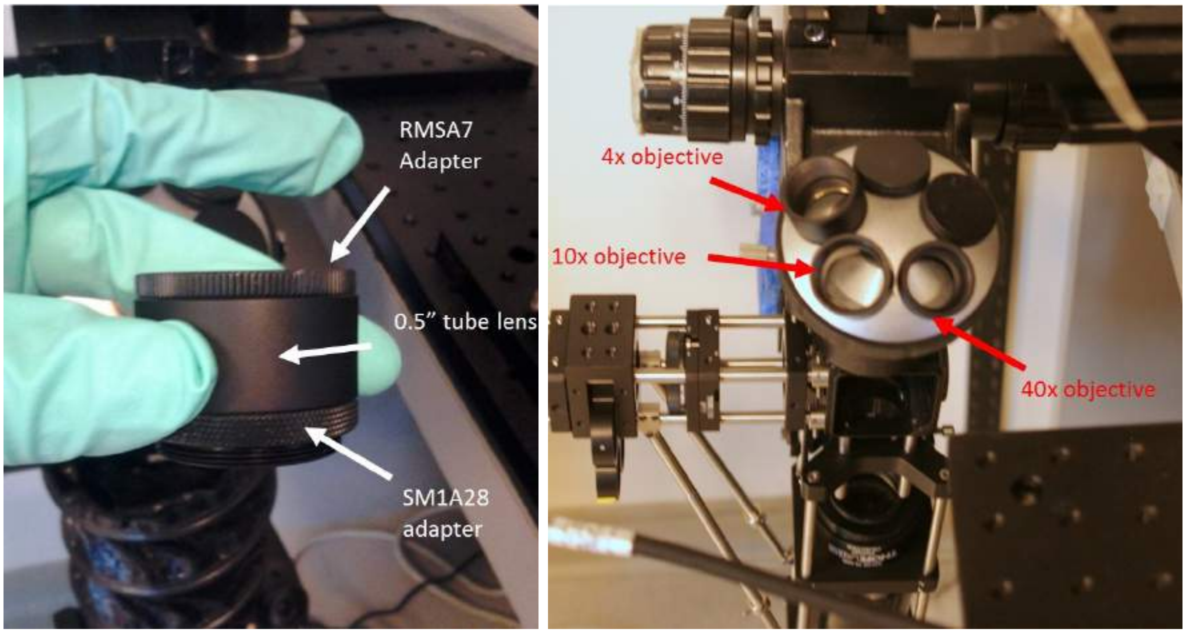

Figure 68: Order of parts for the 4x/40x extension (left), placement of objectives on the objectives platform of the focus block (right)

10.12 Camera & Filter Cube

1. Align the QSI camera with the T-mount adapter at the top of the upright cage system.
2. Twist clockwise to thread the camera into place.
3. Insert desired filter cube.

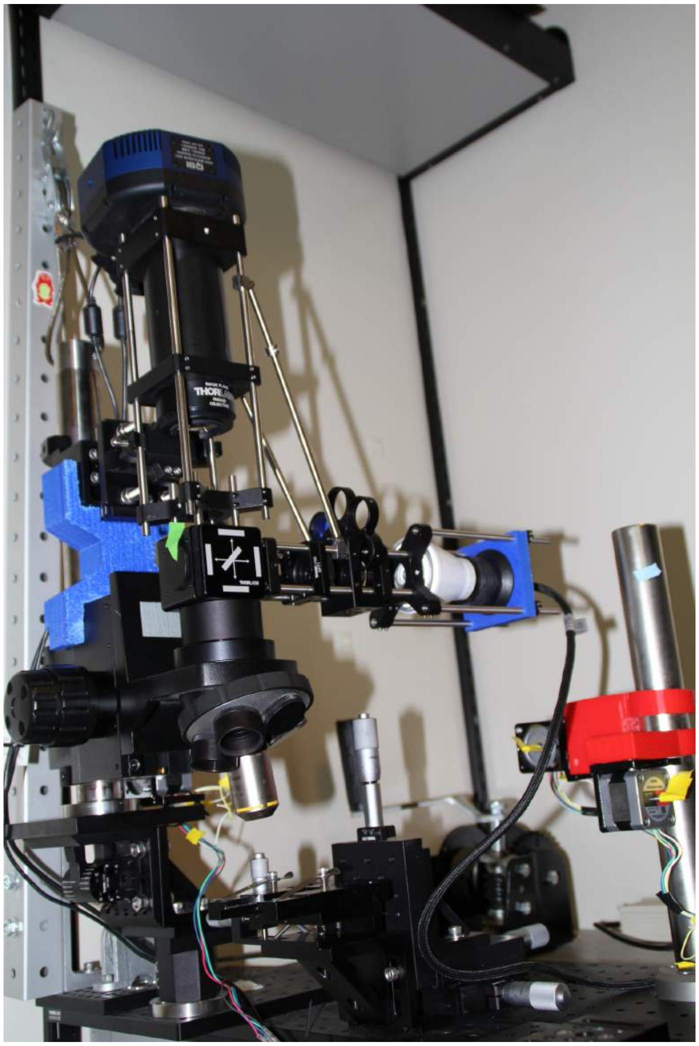

Figure 69: Fully installed SwingScope

10.13 Ellipsometer

Parts:

- Ellipsometer (group assembled in section 8.10)
- 3/4” screws (x2)

1. Locate the 90 degree bracket that is at the base of the ellipsometer (group assembled in section 8.10). Orient the 90 degree bracket so that the pin holes in the diagonal support line up with the left-most row of holes on the 1” spacer of the ellipsometry board.

2. Thread 3/4” screws (x2) through the holes on the diagonal support of the 90 degree bracket into the 1” spacer. Refer to Figure 70.

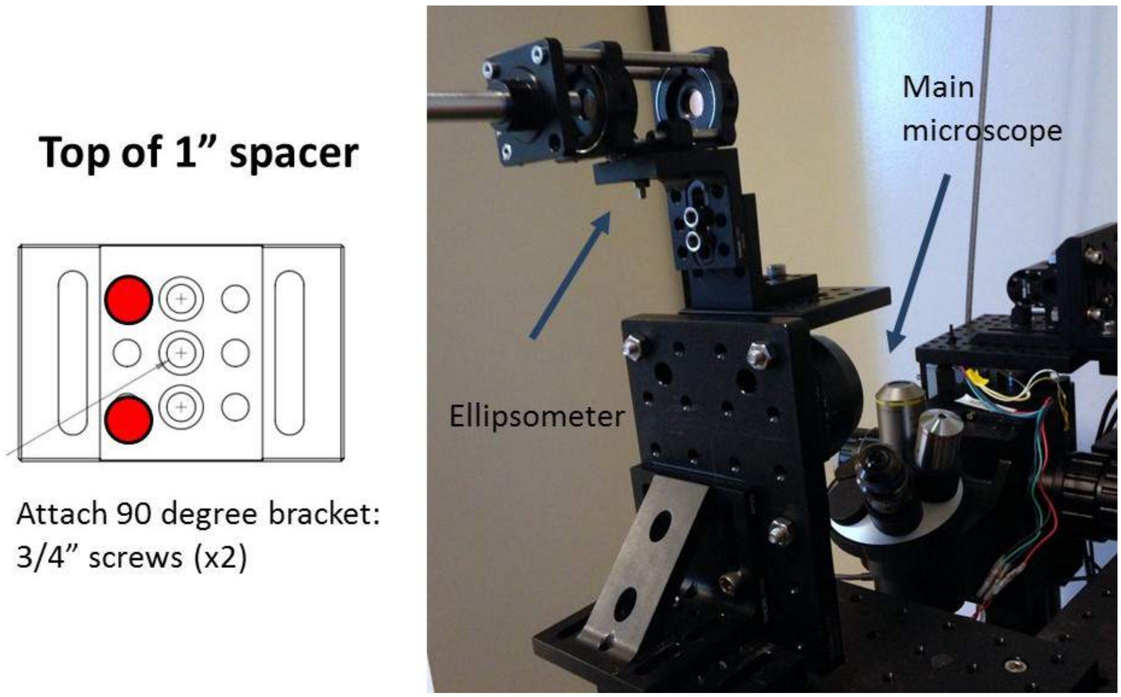

Figure 70: Installed ellipsometer, with the laser cage in the upright position so that it is out of the way while the ellipsometer is not in use

11 Electronics

11.1 Cord Lengths

Note: Please read the instructions in section *13.1 Rotating the SwingScope* before proceeding to the next step.

1. Connect a power strip to an outlet close to the microscope table.
2. Rotate the SwingScope to 90°. Check that there is sufficient length in the cords (camera cords, light cords, etc) to be able to reach the power source when the SwingScope is at its maximum distance in its rotation at 90°.
3. Return the SwingScope to it’s upright position.

11.2 Camera Cords

1. Connect the microUSB cord to the camera and the computer.
2. Plug the power cord into an outlet and the camera.

11.3 Light Source Power Supply/Arduino Control

Parts:

- AC outlet (Xantech AC outlet, serial:15033070010)

1. Attach the AC outlet to a power outlet.
2. Attach the LED light cord to the AC outlet.
3. Attach the Arduino board to the "control input" port of the AC outlet.

11.4 Bundling Cords

1. Unplug all power cords from outlets and power supplies.
2. Place zip tie mounts at desired locations.
3. Thread the zip ties through the squares and bundle the desired cords to keep them organized and out of the way.
4. Once the cords are bundled, tighten the zip ties to keep them in place.
5. Replug power cords and USBs after double checking that they can still reach the power source.

12 Computer & Software Setup

The following steps for setting up a computer for the SwingScope was written by Professor Babak Sanii and edited for general use.

Note on system: We chose a used iMac for our microscope as it has a small footprint, and was generally available in Academia. Micro-Manager will run on Mac or PC, but the camera-control on the mac is through an open-source architecture called gPhoto, whereas on the PC you'll need to spend about $250 to get a copy of a program called DSLRRemoteCamera.

- Mac OS X 10.7.5
- iMac, 2.5 GHz Intel Core i5, 4GB DDR3 RAM

12.1 Overview of Software

A brief overview of the three open-source software used in conjunction with the SwingScope are listed below.

Micro-Manager (full guide here) is a free, open-source program and the main program used for the SwingScope. It can be used in conjunction with ImageJ to control the camera settings and take images. Changes to ISO, exposure, and binning values using on Micro-Manager should be reflected in by changes in the values on the camera screen.

Image J is a free, open source software that can be used in conjunction with Micro-Manager to take images with the microscope. It can also be used to process, edit, and analyze images.

Arduino allows Micro-Manager to control the light source. This is important in preserving the lamp and preventing photobleaching of fluorescent samples.

12.2 ImageJ

ImageJ is paired with MicroManager for imaging. ImageJ can also be used for post-imaging editing and analysis. For help with ImageJ, refer to the guide posted on the ImageJ website. http://imagej.nih.gov/ij/docs/guide/146.html

12.3 MicroManager

12.3.1 Overview of Micro-Manager Open Source Project

Micro-manager is the open source project we use to run the SwingScope. Occasionally we have the need to customize it, or add a feature that's missing. This document starts with the process to download the code and compile a working version of the program. In particular it documents the path Dr. Babak Sanii took while changing the gphoto2 camera interface so it can properly control the Canon 20D, using OS X. Much of my knowledge about this process came from the generous insight and support of Koen De Vleeschauwer ¡kdv@kdvelectronics.eu¿ in the Netherlands. There are alternate instruction, more general for any adapter, here:

http://valelab.ucsf.edu/~MM/MMwiki/index.php/Building_Micro-Manager_Device_Adapters
and here:

http://valelab.ucsf.edu/~MM/MMwiki/index.php/Full_Build_on_MacOS_X_Leopard

12.3.2 Installing Micro-Manager

1. Start this process with the camera unplugged from the computer and turned off.
2. Download nightly build Micro-Manager1.4.15-20130624.dmg (latest as of 6/24) for mac from www.micro-manager.org:
   (a) http://valelab.ucsf.edu/~MM/nightlyBuilds/1.4/Mac/Micro-Manager1.4.15-20130624.dmg
3. Open downloaded file, drag the "Micro-Manager1.4" folder to the Applications folder.
4. In the applications folder, open the "Micro-Manager1.4" folder.
5. Start ImageJ64.
   (a) Agree to warning about opening something downloaded.
   (b) Enter the information about your institution - this helps the micro-manager program.
6. In the splash-screen that pops up (the one with the spiral picture) select "(none)" and click OK

12.3.3 Configuring Micro-Manager

Micro-Manager sets up a configuration file, which we use to tell it what hardware we have. For the most basic SwingScope we just need it to communicate to the camera.

1. Tools Menu ¿ Hardware Configuration Wizard
2. Create new Configuration ¿ Next
3. Scroll down to "GPhoto", click the arrow to open the folder, select "GPhoto — Gphoto2 Generic Camera Driver" and click "Add..."
4. Give it a nice camera name, like "Canon DSLR" and click ok. Then click "Next"
5. Click next on the next page (Step 3, default devices)
6. Click next on the next page (Step 4, delays)
7. Click next on the next page (Step 5, position labels)
8. Give the configuration file a name "e.g., SwingScope 1.0" and click Finish. It's ok to send the configuration to micro-manager
9. Quit Micro-Manager

12.3.4 Installing mac-gphoto-enabler

When you plug in a digital camera several automatic things can be triggered, e.g., iPhoto starts, that interfere with Micro-Manager. This little script disables the triggering, allowing Micro-manager to work with the camera directly.

1. Go to: `https://github.com/mejedi/mac-gphoto-enabler` (github can be trusted)
2. Click on the "zip" button to download the package
3. Open up the folder you downloaded, and hold a right-click (control-click) on "gphoto-enable.sh" and select "Open With", then select "Other..."
   (a) Scroll down to the "Utilities" folder, then select "Terminal" from within it
   (b) Note: if you ever need to undo this, you'll need to open the "Terminal" program in "Applications/Utilities" then "cd" to the directory of these scripts and type "./gphoto-disable.sh"
4. You can verify whether you installed this correctly by starting a program called "Activity Monitor" in the "Applications/Utilities" folder and searching for anything with PTP in it. While it is running, plug in the camera and turn it on. If anything shows up, the script is not properly installed.

12.3.5 Configuring the Camera Using Micro-Manager

1. Plug the camera into the computer and start micro-manager. Select the configuration file you made earlier in the splash screen.
2. You can do a quick test to see if you have a decent connection by clicking the "Snap" button after the program settles. It should take a picture with the camera and put it on the screen. Now we'll make some presets that will make our lives easier.
3. Next to the "Group" button, click "+"
   (a) Select all the camera options: Binning, BitDepth, CameraName, Exposure, ISO, KeepOriginals, PixelType, ShutterSpeed, ShutterSpeedTracksExposure, TransposeCorrection, TransposeMirrorX, TransposeMirrorY, TransposeXY
   (b) Give the group a name, like "Camera Controls"
4. Give the camera its first preset. Some guidance:
   (a) Always set "ShutterSpeedTracksExposure" to 1, so you can control everything with exposure
   (b) Higher binning lowers your resolution, but increases your sensitivity
   (c) ISO is effectively the "gain" on your camera. Higher numbers mean more noise, but also enable you to see dim images.
   (d) You should put a default value for exposure, say 100 (units are ms)
   (e) You should put the ISO value in the name of the preset - the other values (binning, exposure, etc...) you can change per shot, but the ISO is set in the preset.
5. You can add more presets by clicking on the group you made and then pressing the "+" button next to the word preset. We use the following presets (all are 16 BitDepth, and tracking exposure):
   (a) Brightfield-100ISO (1 Binning, 100 ISO, 100ms Exposure, color PixelType)
   (b) Dim-800ISO (2 Binning, 800 ISO, 500ms Exposure, color PixelType)
   (c) Very Dim-3200ISO (8 Binning, 3200 ISO, 1000ms Exposure, Grayscale PixelType)
6. When you are done click the "Save" button on the top-right of the main window

12.4 Advanced Setup and Customization of Micro-Manager

Please skip to "Arduino Board for Shutter Control" on page - if Micro-Manager is operating correctly and no new customizations/modifications of Micro-Manager are needed.

The following sections provide a more in depth explanation of some of the software run by the SwingScope in the case an additional feature/customization needs to be added, but is not necessary for the SwingScope to operate. This was included also because there has been some known communication issues between older versions of the software and some cameras such as the Canon20D when attempting to image.

12.4.1 What is source control?

As the code for an open-source project is freely available, we can download everything we need onto our own computer, and compile our own version. But let's say we do this and produce a feature that we think others would like - we'd like to share it with the world, to contribute the changes we made back to the open-source repository. The general approach to do this is:

1. Download all the code from the repository, and compile it to prove to yourself you have a working version.

2. Make changes to your copy of the code, and test it to confirm you are not introducing bugs. Comment your code like crazy.

3. Have someone knowledgeable go through your code for revision - this important step is called "code review"

4. This process can take some time, so before sharing your code redownload the code from the repository and verify your changes one more time.

5. Check your code changes against the depository using a "diff" command.

6. The next time that the repository recompiles the program (usually once a day, late at night) your changes will be in the latest version of the program online! Download, test it, and share your results.

12.4.2 Downloads Needed for Editing

1. Get Apple's Xcode

   https://developer.apple.com/xcode/

2. Start XCode and under XCode¿Preferences menu select "Downloads" tab and install Command Line Tools.

3. Install MacPorts. If you have macports update it in the terminal with: sudo port -v selfupdate (the first time you use sudo you'll need the admin password)

   http://www.macports.org/install.php

4. Use macports to install gphoto2 freeimage and zlib. From the terminal (/Applications/Utilities) type:

   (a) sudo port install gphoto2
   (b) sudo port install freeimage
   (c) sudo port install zlib
   (d) sudo port install automake
   (e) sudo port install autoconf
   (f) sudo port install pkgconfig
   (g) sudo port install pcre
   (h) sudo port install boost

5. Use "svn" (a built-in source control program) to get the micro-manager source and put it into /trunk
   (a) cd
   (b) svn co `https://valelab.ucsf.edu/svn/Micro-Manager2/trunk`
   (c) cd /trunk
   (d) svn co `https://valelab.ucsf.edu/svn/3rdpartypublic/`
6. Download the latest version of Micro-Manager from `http://valelab.ucsf.edu/~MM/nightlyBuilds/1.4/Mac/`

12.4.3 Setting up the Build Environment to Edit Camera Interface

1. Go to /trunk/DeviceAdapters/SimpleCam/gphoto and edit test.cpp
   (a) main() should be "int main()"
2. Modify the Makefile.test by adding -lfreeimageplus to the LDFLAGS, if not already done.
3. make -f Makefile.test
   (a) This compiles the "test" command line program. (optional: Hack it until it takes a picture with your camera.)
4. cd to the 'trunk' directory. Edit the file /trunk/mmUnixBuild.sh. Change all occurrences of 'libtoolize' to 'glibtoolize', if needed.
5. Run mmUnixBuild.sh.
   (a) ./mmUnixBuild.sh
   (b) If there error that comes up try sudo and run mmUnixBuild.sh again
6. We tweak the file DeviceAdapters/configure a bit, so it finds libfreeimage and libfreeimageplus in /opt/local
   (a) `FREEIMAGEINCDIR=NO, for x in /usr/local/include /usr/include ${thirdpartypublic}/FreeImage/Dist /opt/local/include`
   (b) `FREEIMAGELIBDIR=NO, for x in /usr/lib /usr/lib64 /usr/local/$ARCH/lib /opt/local/lib`
   (c) `FREEIMAGEPLUSINCDIR=NO, for x in /usr/local/include /usr/include ${thirdpartypublic}/`
   (d) `FREEIMAGEPLUSLIBDIR=NO, for x in /usr/lib /usr/lib64 /usr/local/$ARCH/lib /opt/loc`
      i. If the config is locked, make changes in a duplicate of the file named "config copy" and save it in the same place as the original config file.
7. Run configure
   (a) cd trunk

(b) LIBZLIB=/opt/local/lib/libz.a CPPFLAGS=-I/opt/local/include LDFLAGS=-L/opt/local/lib ./configure –without-core –without-imagej –prefix=/opt/local

   i. LIBZLIB=/opt/local/lib/libz.a CPPFLAGS=-I/opt/local/include LDFLAGS=-L/opt/local/lib ./configure copy –without-core –without-imagej –prefix=/opt/local

8. Check configure says

   (a) checking for GPHOTO2... yes, found
   (b) checking for libFreeImage headers... found
   (c) checking for libFreeImagePlus headers... found

9. Now we compile some code which all drivers have in common

   (a) cd /trunk/MMDevice
   (b) make

10. Now we go to the directory with SimpleCam. Edit the Makefile.

   (a) cd trunk/DeviceAdapters/SimpleCam
   (b) line 386: (in some versions)

      i. Change:

```
$(libmmgr_dal_GPhoto_la_LINK) -rpath $(pkglibdir) $(libmmgr_dal_GPhoto_la_
```

      ii. Into:

```
$(libmmgr_dal_GPhoto_la_LINK) -rpath /dummy $(libmmgr_dal_GPhoto_la_OBJECT
```

   (c) Now compile. Type "make"

11. The file you're looking for is

```
.libs/libmmgr_dal_GPhoto
```

   (a) Check that it works:

      i. check

```
otool-L.libs/libmmgr_dal_GPhoto
```

      ii. should list: a bunch of libgphoto2 items, and a libexif, at least

   (b) if it looks like that, copy this file to your Micro-Manager directory, overwriting the existing file

```
libmmgr_dal_GPhoto
```

   (c) `cp .libs/libmmgr_dal_GPhoto /Applications/Micro-Manager1.4/libmmgr_dal_GPhoto`

Note: The shortcuts taken in building the driver mean the compiled driver only will work on your mac, for a single hardware architecture and a single micro-manager version. That's good enough for us. Once the code works, we upload the code to the svn, and the next nightly build will contain the driver, compiled for all macs. We've compiled the device driver for a single architecture (32 or 64 bit Intel), so the device driver "GPhoto" will work with only one of ImageJ or ImageJ64. On my mac, if I try to start up ImageJ, I get an error message "wrong architecture". mageJ64 works fine. If on startup you get the error message "Device interface version mismatch" then the downloaded micro-manager source and the downloaded micro-manager binaries are a different version. Try downloading the latest and greatest sources and/or the latest nightly build. They ought to match.

12.4.4 Useful Micro-Manager Debugging Tools:

To see the Micro-Manager debug log:

1. start micro-manager, and in the Tools menu choose Options. Make sure "Debug Log Enabled" is checked. Close.
2. Restart micro-manager if necessary. Check a file "CoreLogXXX.txt" is in the micro-manager directory. Check the file contains lines which contain the words "Device GPhoto"

To see what shared libraries gPhoto links with:

1. cd /Applications/Micro-Manager1.4
2. `otool -L libmmgr_dal_GPhoto`

12.5 Implementing Arduino Board for Shutter Control

The Arduino board allows us to control the shutter and input/outputs.

Generally, follow instructions here http://valelab.ucsf.edu/~MM/MMwiki/index.php/Arduino

Specifically:

1. Plug in the Arduino to the computer and start the Arduino program
2. Under tools, select the first usb serial port (/dev/tty/usbmodem. . .)
3. Open the "Arduino Firmware" file from the valelab.ucsf website and "Upload" it (the arrow button in the toolbar)
4. After it finishes (output in the bottom of the window), quit the program
5. Start Micro-Manager, and start the HW Wizard
6. Add the Arduino, when the window pops up tell it to scan (it should find the arduino)
7. Add all the peripheral devices and use the default settings
8. In the "Define labels" call state #32 "LED Control" (this corresponds to pin 13 on the board)

9. When done, add a group with "Aruidno Switch-Sate" "Arduino Switch-Label" and "Arduino Shutter-OnOff"
10. Set the Switch State to "32" (for pin 13), and create presets with the On/Off set to 0 (Shutter) and 1 (always on)

13 Operating the SwingScope

13.1 Rotating the SwingScope

Caution: Please take care in rotating the SwingScope! Read this procedure in its entirety before attempting rotation for the first time.

1. Check the amount of tension in the metal cable before doing rotation. The cable should be relatively tight when it is in it's upright position.
2. Raise the objective away from the sample stage to avoid scratching them in the next steps.
3. Move the sample holder out of the way so that it will not come into contact with the objective when the microscope is rotated.
4. Remove the pin from the side of the rotating bracket, but keep one hand on microscope's metal post if this is your first time attempting rotation.
5. Turn the winch counter-clockwise to release more of the cable and bring the microscope to the desired angle. The angles are marked on the side of the rotating bracket. **Note:** If doing inverted images, be particularly careful once the microscope is past about 120 degrees as the microscope can swing during the last 60°. Keep a hand on the microscope during the process of rotating it and let it down gradually.
6. When the microscope is at the desired angle, check the tension in the cable. It should be relatively tight.
7. Once the lines on the rotating bracket are lined up for a specific angle, reinsert the pin and check the tension in the cable.
8. When bringing the microscope back to its upright position, make sure the cable always has tension so that it is properly wound on the spool. Once it is in the upright position, follow the same procedure of aligning the markings on the rotating bracket, reinserting the pin, and checking the tension in the cable.

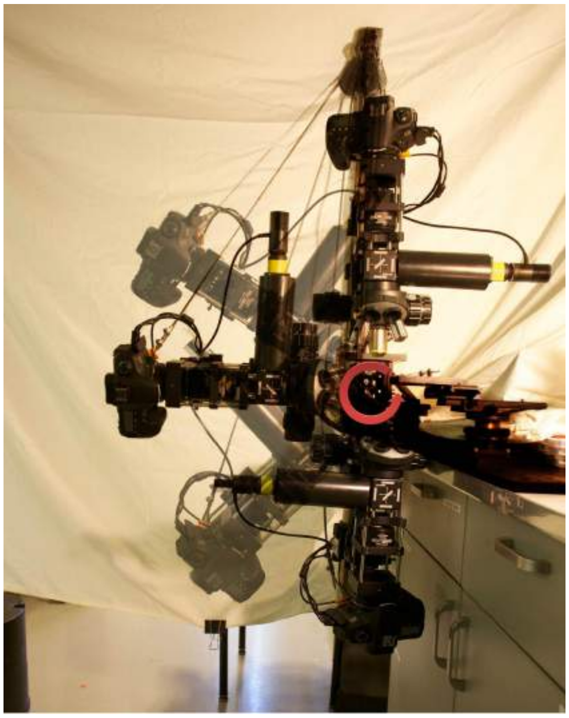

Figure 71: 180°rotation about sample

13.2 Changing Filter Cubes

1. If needed, swap out the brightfield cube for the fluorescence cube while taking care to not accidentally move the sample. To remove/insert a cube, hold the cube by its top and bottom edges and pull/push straight from the cube.

2. Label and store different cubes in a secure location to prevent dust from accumulating on the filters.

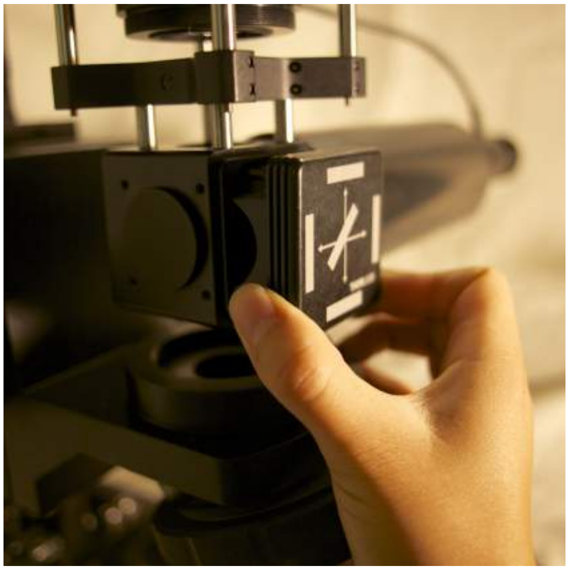

13.3 Adjusting the Focus

1. Focus using the lowest power of magnification first, then increase magnification.
2. Adjustments should be made very carefully to avoid smashing the objective into the sample. Know which way turning the focus knobs raises or lowers the stage.
3. Move the objective away from the sample holder before loading a sample to avoid accidentally scratching the objective lens.
4. Place a sample into the sample holder. The sample may be moved in the x,y, or z direction by adjusting the knobs at the end of the sample holder axes.
5. Look at the sample using the monitor and use the coarse focus knobs to find the sample. Tip: Look at the slide and lens from the side when doing this to avoid smashing the objective into the slide. When the point of light hitting the sample is at it's smallest diameter, the objective is close to it's working distance and the sample is close to being in focus. This is usually a good starting point.
6. Use the fine focus knobs to obtain a crisp, in-focus image of the sample.

13.4 Handling and Cleaning

Below are some general guidelines to keep in mind when operating the SwingScope

1. DO NOT touch the lenses of an objective with your fingers or bare hands as the oils on our hands can damage the glass.
2. In the case that lenses or objectives need to be handled for cleaning, always wear gloves and only use low pressurized air to clean off particles. Avoid using high pressure air as high velocity particles can scratch the lens.
3. Anytime a piece of the microscope is removed such as an objective or lens, it should immediately be placed in a storage container to prevent dust from collecting.

13.5 Storage after Imaging

Proper storage of the SwingScope is essential in preventing dust accumulation that can affect image quality. Below are some important guidelines that applies when the SwingScope is being assembled and after it is completely installed.

1. If the SwingScope is not in use, it is always best to cover it with a plastic cover to keep out dust. At a minimum keep the tube lens covered when not in immediate use until the final encasing/covering is installed.
2. Cover the camera adapter if the camera needs to be removed and always place the lens cap onto the camera after removal.
3. To minimize dust falling onto the camera lens, close the shutter by switching the preset setting to "Fluorescence Image" mode in Micro-manager when finished using the microscope.

14 Imaging Checks & Tests after Installation

These checks and tests should be done after the SwingScope is assembled to ensure proper setup for imaging and before any tubing is installed (i.e encasing upright cage system and Kohler illumination system to maximize light usage). **Before performing the checks and tests please read section *15 Imaging***

14.1 Testing Kohler Illumination

1. View the sample through the camera.
2. Adjust the coarse focus knobs to make sure it is in focus. Use the fine focus knobs for small adjustments.
3. Take some test shots to see if the sample is evenly illuminated. It might be easiest to observe this using a fluorescent sample such as a drop of Nile Red dye spread evenly across an area that will fill the entire field of view of the microscope.
4. Adjust the placement of the lenses in the Kohler Illumination system if needed.

14.2 Adjusting Aperture Distance

The aperture in the Kohler Illumination system may need to be adjusted. It is easiest to adjust the aperture by viewing the edges of the aperture on the image of a fluorescent sample such as a small area of fluorescent dye placed on a slide.

1. Place a sample into the sample holder.
2. Close the aperture to its smallest size.
3. Move the aperture on the rails of the Kohler system until the edges of the aperture appear crisp on the image when you open and close it.
4. Tighten the setscrews on the side of the aperture holder to lock it in place.

15 Imaging

15.1 Terminology

The camera settings can be adjusted using the Micro-Manager software (see "Microscope Software").

Note on the System: We are currently using the QSI 628 camera, which was chosen for its versatility. In the past we have also used the Canon 60D camera which has good versatility and a rotatable live screen.

1. Some useful terms are discussed in the section below are useful for photography in general but also applies to the Micro-Manager program which is able to control the camera settings.
2. **ISO** is the sensitivity of the camera to light. The lower the ISO the less sensitive the camera is to light and the higher it is the more sensitive it is. Higher ISO means more noise/grainy images, but also enable you to see dim images.

3. **Exposure** is the amount of light that is allowed to fall on the sample. This is associated with adjusting how quickly the shutter closes.If the shot is exposed too long the image will be washed out. Too short and the image will be dark.

4. **Binning** combines pixels and reduces noise to improve the signal-to-noise ratio but narrows the field of view. This can be easily changed using Micro-Manager.

15.2 Imaging Using Micro-Manager

The procedures outlined below are summarized for our lab use. Please consult the full Micro-manager guide on the original website at http://www.micro-manager.org/wiki/Micro-Manager_User%27s_Guide

15.2.1 Basic use: Taking Images and "Snap" Mode

1. Start Micro-Manager (orange icon with an image of a microscope).

2. Select the desired configuration file.

3. **Note:** It is recommended to always start out with brightfield, even if the final goal is to take images fluorescent samples. Brightfield can be used to find the region of interest and focus on the sample to ensure the a good fluorescent image. Remember to keep the light on low when using brightfield to avoid excessive photobleaching.

4. Click the "Snap" command in the top left corner of the program to take a test image. If it is properly communicating with the camera the shutter should go off and a window will pop up with the image.

5. The image can be analyzed using ImageJ or saved as a single image by clicking "Save." Save and label photos under a descriptive title with initials and sample description under a folder with today's date.

15.2.2 Live Mode

1. Use "Live" to see a live feed of the sample. How quickly the live view updates depends on how quickly the camera is able to respond and close the shutter so it won't be a constant feed. Images cannot be taken in this mode.

2. If an error message comes up saying there is not enough memory to enable live feed, in ImageJ go to Edit, Options, Memory & Threads and increase the memory available to the program. Alternatively, you can also increase the binning (cuts down the field of view, increases sensitivity by combining pixels) so the files are smaller for the live feed.

15.2.3 Album

Use the "Album" button to save a series of images.

1. The first time you click the "Album" button a new window will open up. This will be the first image in the album. Every time you click "Album" afterwards will add the current image to the Album (if you don't hit album for an image it won't saved).

2. Click "Save" to save all of the images to the album.

3. Save the current album.

15.2.4 Multi-dimensional acquisition

1. Use Multi-dimensional acquisition to get multi-image stacks and control how you want multiple images to be taken.

2. Time points: this allows you to set how many frames you want and the time interval between each frame. Set it to "0" if you want as little time between each frame as possible.

3. Multiple Positions and Z Slices: This is for microscopes that have a movable stage. Our microscope is not set up for this so skip this setting.

4. Channels: These allow you to control the settings for the various configuration preset groups all in one place.

5. Check the box that enables each image to be saved as it's taken and designate a directory. This should prevent the error message about lack of memory from coming up.

6. Select the channel group and select the exposure for each preset.

7. You can also select which frames you want to use which preset on. For example if you wanted to use the Brightfield preset on the first frame, but not the second frame, you can specify the number in "Skip Frame."

16 Ellipsometry

16.1 Terminology

- **Ellipsometry** is a technique that uses changes in the polarization of light after it is reflected off a sample to measure thicknesses of thin films.

- **Imaging ellipsometry** is a variant, that also allows imaging of the surface of a sample to allow visualization of changes in the thicknesses as time goes on. By the nature of ellipsometry, images are taken at an obscure angle meaning that only a small area of the sample will be focus at a time. To create a complete image of the sample, the area in focus is moved all the way to one side of the region of interest/sample and then progressively moved to the other side. After each incremental movement to the next spot in the sample, the focus is readjusted and an image is taken. All of the photos are then compiled into a complete, in focus image.

- **Nulling** is a procedure where the polarizer and analyzer are adjusted so that the substrate appears completely dark.

- **Delta and Psi:** There are two main values obtained experimentally from ellipsometry, Ψ **and** Δ. The values are functions of the angles of the polarizer (P) and analyzer (A) when null conditions are achieved.

$$\Psi = A_1 \tag{1}$$

$$\Delta = 2P_1 + \pi/2 \tag{2}$$

Psi and Delta can be combined with the complex reflection ratio with respect to the p and s polarization states and formally defined as:

$$\tan \Psi e^{i\Delta} = \frac{r_p}{r_s} \tag{3}$$

If the thickness of a film is known along with the refractive indexes, the Psi and Delta of that sample can be predicted. An example for how the psi and delta are affected by film thickness can be seen in the graph below

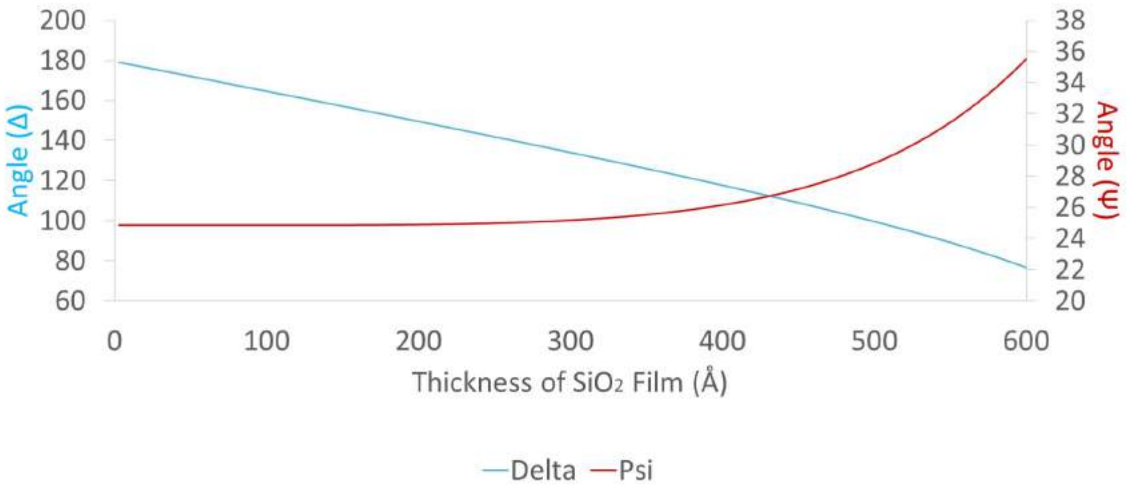

Figure 72: Relationship between Psi and Delta as a function of the thickness of a Silicon Oxide film. For this system, the angle of incidence was said to be 60° and a 532nm wavelength laser was used. The refractive indexes for the air, SiO_2 film, and silicon substrate are as follows: 1.0, 1.97, and 4.14 respectively.

16.1.1 Setting up for Ellipsometry

1. Slide the analyzer (group assembled in section 8.10.1) onto the posts above the filter cube. Tighten the set screws to hold it in place.

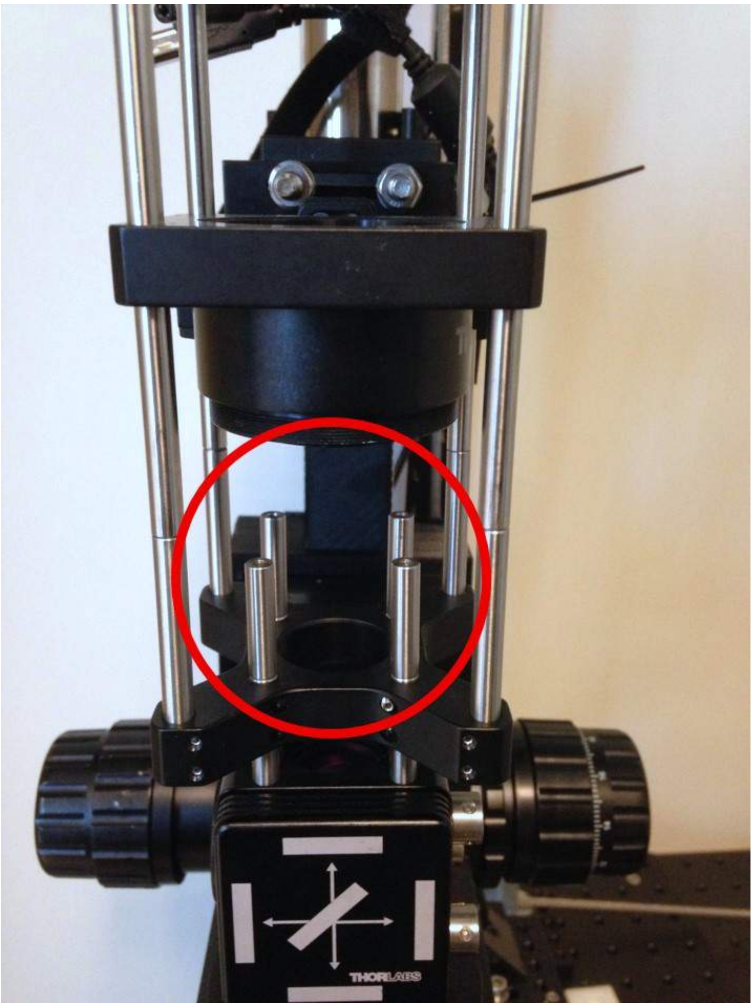

Figure 73: Posts above the filter cube where the analyzer is housed

2. Adjust the height of the ellipsometer as needed to make sure that the center of the rotation mount is directly in line with the pivot point on the main microscope.

3. Attach the laser to the laser power supply (LDS5) and turn it on.

4. Fix the compensator at a 45 °angle.

5. Choose an angle of incidence and rotate the main microscope to that angle.

6. Rotate the ellipsometer so that it is at the same angle as the microscope in step 5. Use digital levels to make sure the angles are the same for the main microscope and the ellipsometer arm.

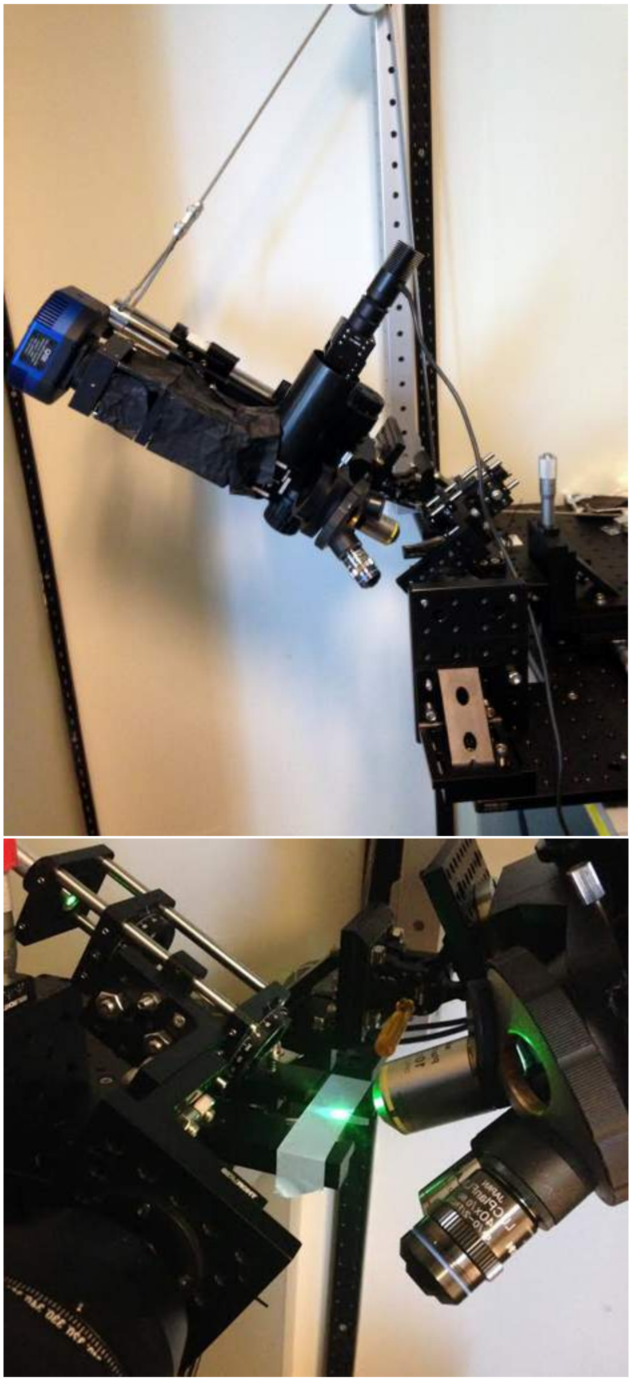

Figure 74: SwingScope ready for ellipsometry

16.2 Getting Delta and Psi Measurements

1. Rotate the polarizer and analyzer one at a time to achieve null conditions.
2. Determine the Delta and Psi values using the equations outlined in section 16.1.

16.3 Imaging Ellipsometry

1. Rotate the polarizer and analyzer one at a time to achieve null conditions.

2. Only a thin band of the region of interest is in focus at any particular time due to the nature of ellipsometry where images are taken at obscure angles. To get a compiled image take a series of images by moving incrementally across the sample, adjusting the focus, and taking an image at each spot.

3. Compile the images to get a complete in-focus image. See Figure 75 for an example.

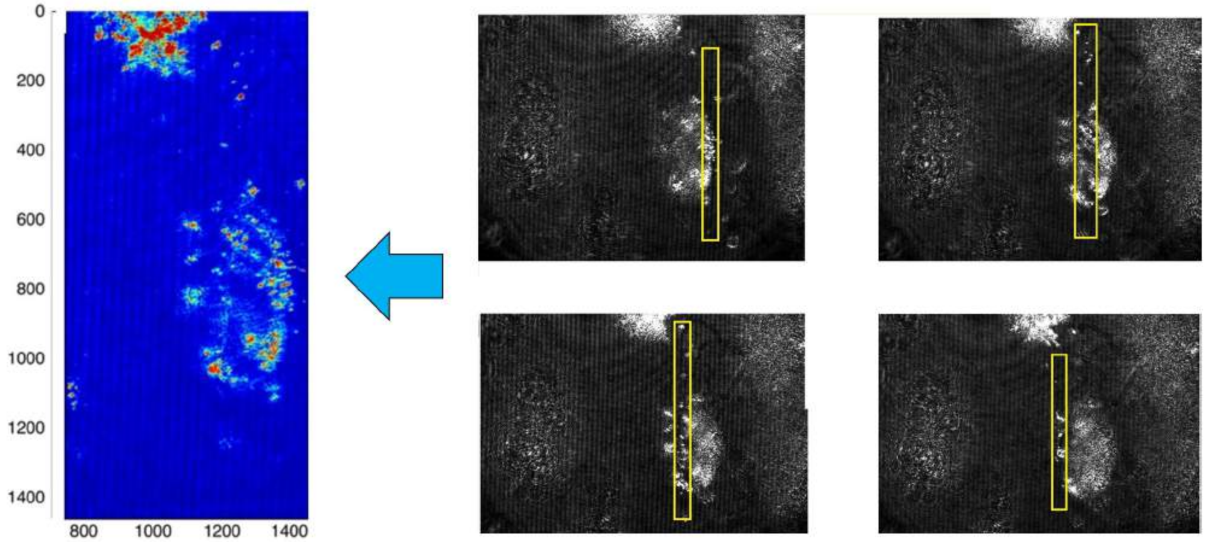

Figure 75: A compilation of a stack of images taken during an ellipsometry experiment. On the right are 4 photos taken out of the stake of images used to create the full image

17 Tubing

Do this only AFTER the quality of the illumination is satisfactory and checks/tests for the imaging quality are performed and are satisfactory.

Tubing of various sizes and materials can be used to encase parts of the microscope to minimize light leakage. This is recommended if the microscope is no longer being customized or adjusted. However, it is also possible to use blackout foil/paper while working on the microscope.

Possible Tubing sources:

- Thorlabs 2" tube cover lens (SC2L24)
- PCP piping from Home Depot

Key areas to cut out tubing for:

- Upright right cage system, particularly between lenses
- Kohler Illumination Arm

18 Troubleshooting

18.1 Dark or Blurred Edges: Alignment

If there are dark circles or blurring at the edges of the field of view, it might be an indication the LED light source or component in the upright cage system is not properly aligned. The H-shaped piece was designed to address this issue and automatically align the detection and objective. However, there may be small adjustments needed if the H-shaped piece experienced warping while 3D printing.

Try the following:

1. The alignment of the upright cage system can be checked visually by removing the camera and peering down the upright cage system. It can also be checked by imaging a sample such as a KimWipe or tissue.
2. To adjust the light source, take out the filter cube and turn on the light. The light should fall in a nice even circle on the circular backing where the filter cube used to be.

18.2 Blurred Stripes: Leveling

If there are blurred regions or "stripes" that are more clear than others, this may be an indication that one of the parts in the system is not level/tilted. Common places to check include the set of spacers in the mount-cage extension, the adapter that connects to the camera, and across the lens tube holder. Use a leveling tool to compare the surface where the sample sits and areas that may be tilted.

18.3 Light Source Communication: Arduino

If you are having trouble with controlling the light source/automatic shuttering of the light source between images, it is most likely an communication issue with the Arduino board. First, check the cable that connects the Arduino board to the light source to make sure the connection is sound.

If there is still trouble communicating with Arduino try the following:

1. Make sure the wire that connects Arduino to the light source is intact and has not become unsoldered
2. Make sure that the RED cable is inserted into pin 13 on the Arduino board
3. Make sure the GREEN wire goes into pin 14
4. Press the orange reset button on the Arduino board and restart Micro-Manager
5. Try unplugging and plugging back in

18.4 Light Pollution and Leakage

If the image seems to have some unequal illumination due to external light sources (i.e. from lights in the room or from the computer glare) use blackout paper to cover any gaps. For a more permanent solution, refer to section *17 Tubing* to encase critical components of the microscope.

Critical areas to check include:

1. Gap between the filter cube and the focus block
2. Upright cage system
3. Connection between the Kohler Illumination arm and the upright cage system
4. Areas near lenses and the LED
