# Supplementary figures and images for: An Assemblable, Multi-Angle Fluorescence and Ellipsometric Microscope
Source: PLoS One. 2016 Dec 1;11(12):e0166735. doi: 10.1371/journal.pone.0166735 (PMC5132209; doi:10.1371/journal.pone.0166735)

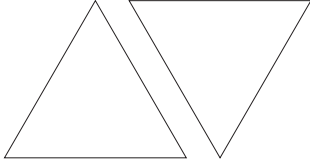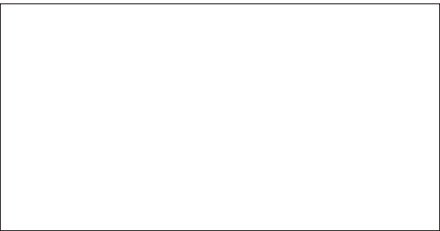

Supplement: S1 File — (ZIP) [file pone.0166735.s002.zip › cut_mk2.pdf]

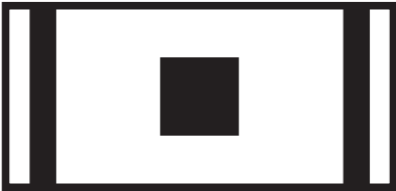

Supplement: S1 File — (ZIP) [file pone.0166735.s002.zip › engrave_mk2_updated.pdf]
