# Supplementary material for: An Assemblable, Multi-Angle Fluorescence and Ellipsometric Microscope
Source: PLoS One. 2016 Dec 1;11(12):e0166735. doi: 10.1371/journal.pone.0166735 (PMC5132209; doi:10.1371/journal.pone.0166735)
Supplement: S3 File — (PDF) [file pone.0166735.s004.pdf]

SUPPORTING INFORMATION S3 FILE FOR: AN ACCESSIBLE-TO-BUILD, MULTI-ANGLE FLUORESCENCE AND ELLIPSOMETRIC MICROSCOPE

VICTORIA NGUYEN, JOHN RIZZO, BABAK SANII

Contents

Resolution vs. variously priced tube lenses 1
Combining slices of oblique images to create a single focussed image 2
Map of P vs. A increased ellipsometric sensitivity 3

Resolution vs. variously priced tube lenses

Three tube lenses of different price ranges were selected for the analysis and are listed in Table 1. As a measure of imaging resolution we tested the effect of each tube lens on the microscope's point spread function.

**Tube lens**	**Description**	**Cost**
ITL200	Infinity corrected	$450
AC508200	Fused doublet lens	$125
LA1979	Single plano-convex lens	$27

Table 1. 200mm focal length tube-lenses analyzed, as purchased from Thorlabs

In order to determine the point spread function, we deposited a 1:100 dilution of 0.5 μm fluorescent beads onto a glass slide. 0.5 μm is smaller than the resolution of the microscope at 10X, and thus the spread of the image of the beads will be a suitable approximation of the relative point spread functions.

We then used ImageJ to determine the intensity profile across a single bead with each tube lens. The intensity profile was fitted to a Gaussian curve using non-linear least squares fitting implemented in Excel. The width of a fluorescent bead at the point where its intensity is half of the maximum intensity, called the Full Width at Half Maximum (FWHM), is then used to characterize the point-spread-function and also serves as a short-hand measure of resolution.

There was a significant difference in the resolution of the microscope between the more expensive ITL2000 infinity-corrected tube lens and the cheaper AC508200 and LA1979 tube lenses when they were used with the same Nikon 10X objective (Figure 1). .

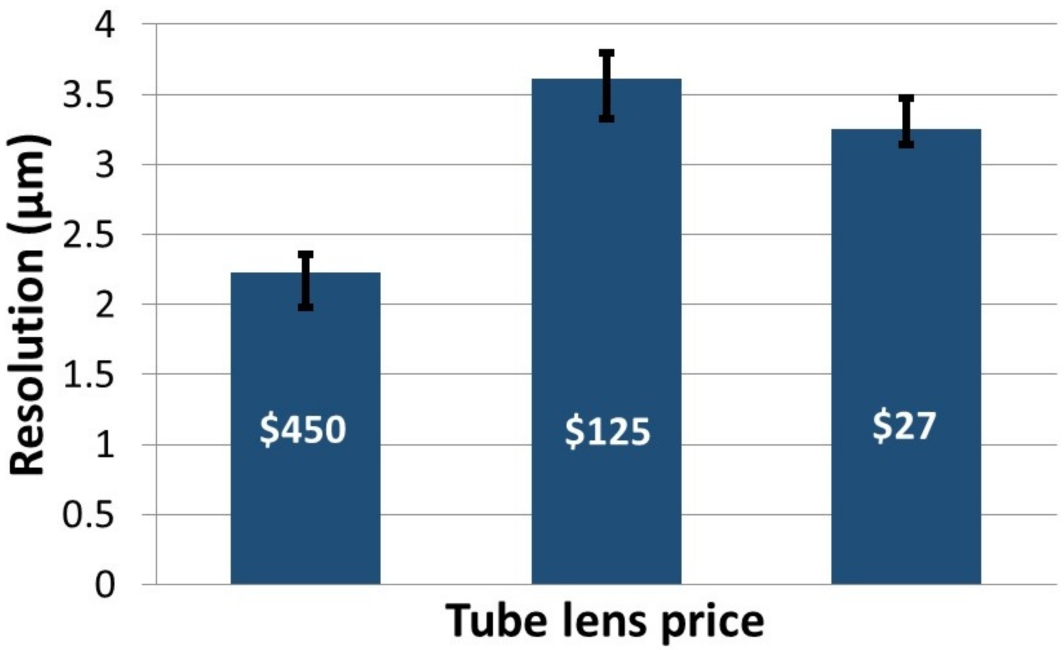

FIGURE 1. System resolution with the ITL200, AC508200 and LA1979 tube lenses, in FWHM μm. Prices of the three lenses are listed.

COMBINING SLICES OF OBLIQUE IMAGES TO CREATE A SINGLE FOCUSSED IMAGE

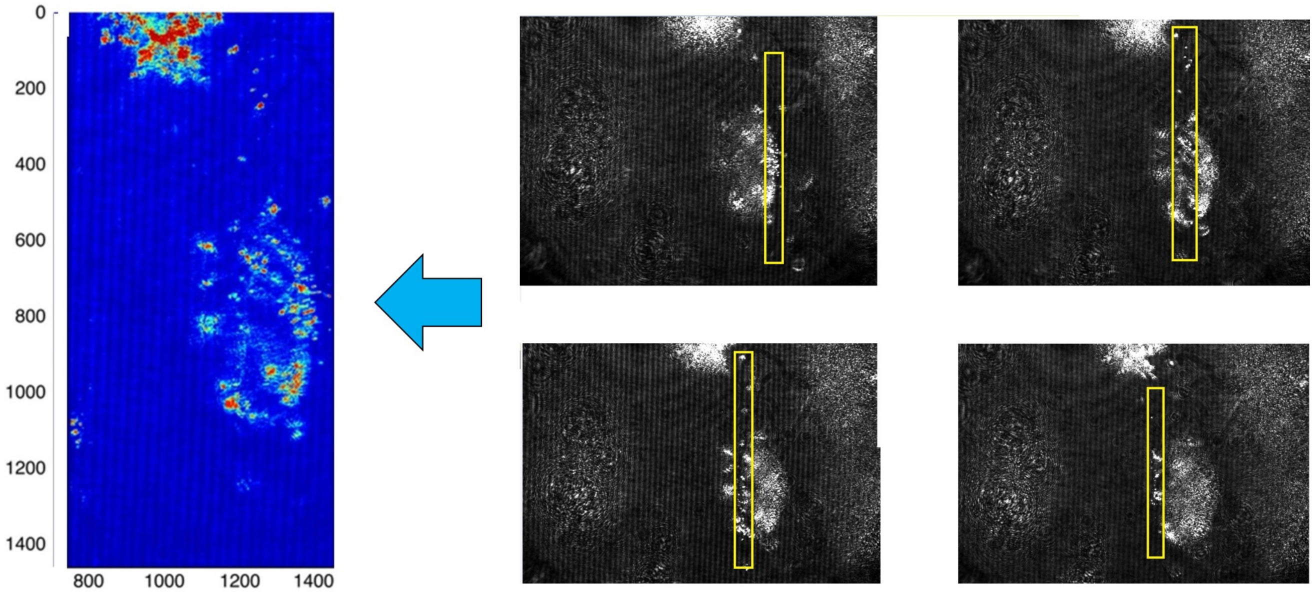

FIGURE 2. A stack of ellipsometric contrast images were taken at a focus points across the sample (dust on Si, in air). Due to oblique incidence (60 degrees) only a portion of each image is in focus (e.g., four images on right). Slices of each image were aggregated to produce a single focussed image (left).

Map of P vs. A increased ellipsometric sensitivity

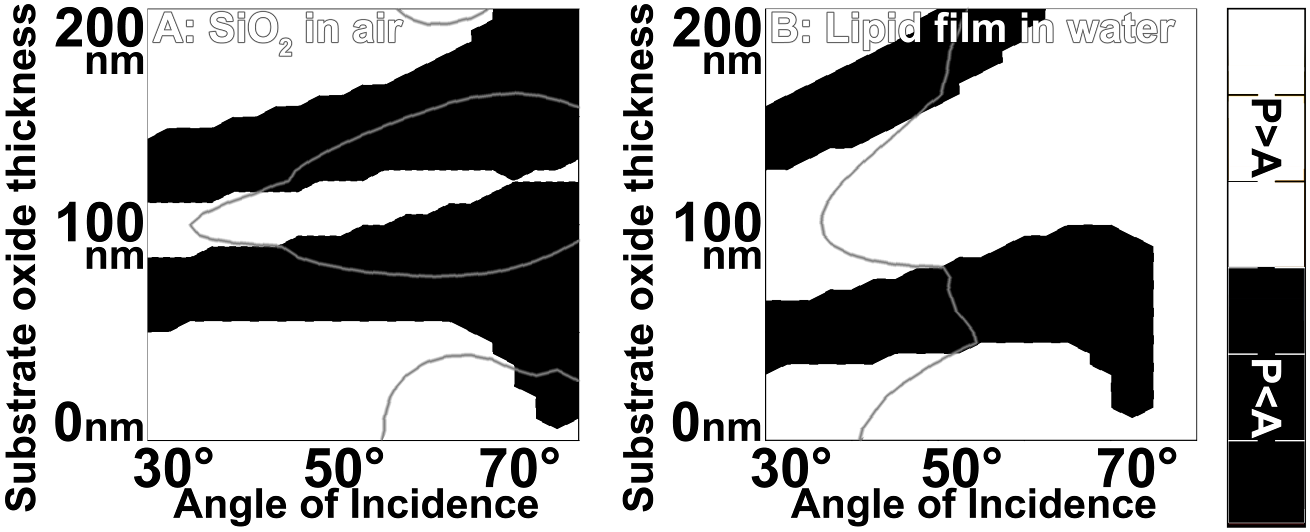

Figure 3. When optimizing ellipsometry sensitivity with angle of incidence and substrate oxide thickness (e.g., Fig. 3 of main text), in different regions P is more sensitive than A, noted here by black/white. Gray line corresponds to greater than 0.5 degrees per nm. **a:** P vs. A sensitivity advantage for 1nm of additional Silicon oxide in air **b:** P vs. A sensitivity advantage for 1nm of lipid material in water
